# Optimal intensive care outcome prediction over time using machine learning

**DOI:** 10.1371/journal.pone.0206862

**Published:** 2018-11-14

**Authors:** Christopher Meiring, Abhishek Dixit, Steve Harris, Niall S. MacCallum, David A. Brealey, Peter J. Watkinson, Andrew Jones, Simon Ashworth, Richard Beale, Stephen J. Brett, Mervyn Singer, Ari Ercole

**Affiliations:** 1 Division of Anaesthesia, University of Cambridge, Cambridge, United Kingdom; 2 Bloomsbury Institute of Intensive Care Medicine, University College London, London, United Kingdom; 3 Nuffield Department of Clinical Neurosciences, University of Oxford, John Radcliffe Hospital, Oxford, United Kingdom; 4 Department of Intensive Care, Guy’s and St. Thomas’ NHS Foundation Trust, St. Thomas’ Hospital, Westminster Bridge Road, Lambeth, London; 5 Centre for Perioperative Medicine and Critical Care Research, Imperial College Healthcare NHS Trust, Praed St., London, United Kingdom; Beth Israel Deaconess Medical Center, UNITED STATES

## Abstract

**Background:**

Prognostication is an essential tool for risk adjustment and decision making in the intensive care unit (ICU). Research into prognostication in ICU has so far been limited to data from admission or the first 24 hours. Most ICU admissions last longer than this, decisions are made throughout an admission, and some admissions are explicitly intended as time-limited prognostic trials. Despite this, temporal changes in prognostic ability during ICU admission has received little attention to date. Current predictive models, in the form of prognostic clinical tools, are typically derived from linear models and do not explicitly handle incremental information from trends. Machine learning (ML) allows predictive models to be developed which use non-linear predictors and complex interactions between variables, thus allowing incorporation of trends in measured variables over time; this has made it possible to investigate prognosis throughout an admission.

**Methods and findings:**

This study uses ML to assess the predictability of ICU mortality as a function of time. Logistic regression against physiological data alone outperformed APACHE-II and demonstrated several important interactions including between lactate & noradrenaline dose, between lactate & MAP, and between age & MAP consistent with the current sepsis definitions. ML models consistently outperformed logistic regression with Deep Learning giving the best results. Predictive power was maximal on the second day and was further improved by incorporating trend data. Using a limited range of physiological and demographic variables, the best machine learning model on the first day showed an area under the receiver-operator characteristic curve (AUC) of 0.883 (*σ* = 0.008), compared to 0.846 (*σ* = 0.010) for a logistic regression from the same predictors and 0.836 (*σ* = 0.007) for a logistic regression based on the APACHE-II score. Adding information gathered on the second day of admission improved the maximum AUC to 0.895 (*σ* = 0.008). Beyond the second day, predictive ability declined.

**Conclusion:**

This has implications for decision making in intensive care and provides a justification for time-limited trials of ICU therapy; the assessment of prognosis over more than one day may be a valuable strategy as new information on the second day helps to differentiate outcomes. New ML models based on trend data beyond the first day could greatly improve upon current risk stratification tools.

## Introduction

Accurate prognostication is central to medicine [1] and is at the heart of clinical decision making, quality and safety benchmarking, research case-mix assessment / adjustment and policy making. In the intensive care setting, the need for informed clinical decision making is particularly acute, since the burden of treatment and financial costs involved can be very high. Decisions as to whether the patient’s best interests are best served by providing life-sustaining treatment or whether it is instead more appropriate to focus on comfort and symptomatic control must sometimes be made over short timescales yet have profound consequences [2–5].

Assessment of prognosis in intensive care has previously been limited to the first hours/day of an admission. The majority of large prognostic studies have sought to develop clinical scoring systems for objective risk stratification in the early phase of an admission, using physiological measurements, medical history and demographics to predict likelihood of survival [6]. Most of these scoring systems, of which the Acute Physiology and Chronic Health Evaluation-II (APACHE-II [7]) is perhaps one of the best known, are validated for application at around 24 hours after ICU admission.

It is likely that the determinants of survival, and therefore the accuracy to which outcome can be predicted, varies over time since disease states evolve with some (ill-specified) timescale. There has been no investigation as yet into how prognostic ability changes over the course of an intensive care admission. As a result, it is possible that current predictors, being based on a particular time-point, have sub-optimal performance. This is potentially clinically important since it is not clear at what time clinician uncertainty is minimised and therefore at what time point assessment of outcomes are most reliable.

As a concrete motivating example, patients are often admitted for a trial of intensive care, with continuation of treatment dependent on response over a pre-specified time period, usually several days [8]. These time-limited trials of ICU are an attractive concept to ensure that burdensome treatments are not prolonged when they turn out to be ineffective after a period of time. However, in practice, such trials are often incompletely discussed, at least in part because of a lack of appropriate prognostic information [9]. The change of prognostic ability over time is an essential piece of information on which the rationale of time-limited trials of intensive care must be based; it both justifies the practice and informs of the optimal length of trial period. Some work on this has been performed; the optimal duration of such trials has been shown to vary between solid and haematological malignancies [10], but little is known for the general ICU population. While not all patients are explicitly on a time-limited trial, many effectively are, given the possible decision to withdraw life-sustaining treatment, and the competing demands for ICU beds. It is therefore important to determine the optimal time-point for assessment of prognosis in the general ICU population, at which point predictive accuracy will be greatest. A scoring system at a time-point where patients are relatively undifferentiated is likely to under-perform as a risk-adjustment instrument.

The development of temporally calibrated, trend-sensitive prognostic models with the best possible performance is therefore timely. Current prognostic models such as APACHE-II are not designed for repeated application and perform badly in this context [11]. Other systems have been designed for sequential use such as the Sequential Organ Function Assessment (SOFA [12]) and Multiple Organ Dysfunction (MOD [13]) and demonstrate that longitudinal assessment of patient condition is possible by assessment of the maximum deviation of these scores with time [11]. However, these approaches do not incorporate two features of the information naturally used by a human predictor—non-linearity and trend.

That ‘trend’ data may be discriminating seems intuitively reasonable (i.e. a sequential score should be able to detect whether, and how quickly, a patient is getting better). However, at least part of the reason for the absence of assessment of trend information in published systems is the multiplicity of interactions. Published models are generally constructed and calibrated using logistic regression techniques which have the advantage of simple covariate interpretability, and subsequent ease in development of a practical clinical tool. To incorporate several variables measured at several time points into a logistic model which considers both trends and interactions between variables, would require explicit inclusion of a very large number of such ‘interactions’. However, to consider and compare prognostic ability at different time points, all such pieces of trend information should be considered.

Furthermore, illness state is not a linear or even monotonic function of many covariates (for example both high and low white blood cell counts may be associated with sepsis) and variables may interact in significant ways (for example the interaction of vasopressor use and lactate elevation incorporated into the third international consensus definition of sepsis and septic shock [14]). Again, logistic models can accommodate such effects but they must be explicitly introduced.

At least partly as a result of this, the predictive power and generalisability of the current clinical prognostic models is not excellent. Comparisons of APACHE II, III [15] & IV, Simplified Acute Physiology Score SAPS II [16–18], and the Mortality Prediction Models MPM_0_-III & MPM_24_-III [19] yielded area under the receiver operating characteristic curves (AUCs) mostly in the range of 0.75-0.85 [20–22].

Machine learning (ML) classifiers may be advantageous for outcome prediction in that they can handle large numbers of covariates (and therefore temporal data) and learn non-linearities and interactions [23, 24] without the need for them to be explicitly predetermined. These non-linearities and interactions can instead be learned during the fitting of the model. Thus the model can consider all possible permutations, including or ignoring variables, interactions and non-linearities in a manner which need not be determined by the foreknowledge of the researcher [25]. This comes at the expense of needing large volumes of training data and generating a complex model. The increasing availability of electronic health records, however, is making large volumes of medical data available in machine-readable form which reduces the importance of a prognostic model being simple either to construct or implement since this can be effectively achieved in an automated way. Large training sets are now available [26, 27] making the investigation of such approaches feasible.

Some studies have begun to apply machine learning to these intensive care datasets. A series of studies have shown that a particular machine learning approach, applied to MIMIC-III (or its predecessor MIMIC-II), can be used to build prognostic models for both mortality and the development of sepsis. Traditional scoring systems were outperformed by this machine learning approach to mortality prediction in the medical ICU from eight variables measured hourly [28]. This algorithm showed an impressive AUC of 0.88, compared to 0.71-0.75 for SAPS-II, SOFA & MEWS. However, this study was focusing on very short term mortality prediction, with predictions made 12 hours before death or discharge, based on the preceding 5 hours. The same group have also applied machine learning in the early detection of sepsis in the ICU. A series of studies on both the general ICU population and those with Alcohol Use Disorder, in whom traditional scoring systems are less effective, showed significant out-performance by a machine learning classifier on the MIMIC-II & MIMIC-III datasets [29–31]. In both cases, however, the underlying algorithms are not clearly described, and both are available commercially.

MIMIC-III data have also been used to show that an artificial neural network can substantially outperform both SAPS and a logistic regression (AUCs of 0.875, 0.642 & 0.738 respectively) in predicting mortality in patients with acute kidney injury (AKI) in the ICU [32]. In the general ICU population, the potential of machine learning in outperforming traditional mortality prediction tools was suggested in a study which was looking primarily into transfer learning, where a large dataset (MIMIC-III in this case) is used to enhance a smaller target dataset [33]. Beyond mortality prediction, both MIMIC-III and local ICU data were used to build a classifier which predicted failed ICU discharge (re-admission within 48 hours) more accurately than the physicians and the purpose-built clinical scoring system. This study used ‘AdaBoost’, an adaptive boosted ensemble method, using decision tree classifiers as weak learners, and used transfer learning to enhance the local ICU dataset with the MIMIC-III database [34].

Thus the potential of machine learning in improving prognostication in intensive care has been explored. This study sought to use ML techniques not to build a new prognostic model, but as a *tool* to understand the prognostic performance of ICU physiological data as a function of time in the first days after admission. We sought to to identify what prognostic accuracy is possible using common ML techniques. We hypothesized that ML could provide optimal models and therefore be used not only to build better models, but also as a *proxy* for the prognostic information content in the data as a function of time. This information could then be used to identify an optimal time-point for prognostic accuracy and assessing the remaining residual uncertainty. We further sought to understand the information content in trend data currently not explicitly incorporated into prognostic modeling.

## Materials and methods

Calculations were carried out using R version 3.4.4 on Linux. The code was optimized to run in parallel across 16x3.3GHz Intel Xeon cores with a total of 32GB RAM. The code is available at https://github.com/ariercole/TemporalPrediction.

### Dataset

Data used in the preparation of this article were obtained using the Critical Care Health Informatics Collaborative (CCHIC) data infrastructure. The CCHIC was launched in 2013 as a collaboration between five NHS Trusts (11 ICUs). The primary goal of CCHIC has been to establish and maintain cataloged, comparable, comprehensive flows of all retrospective routinely collected patient data from each trust. The ethical approvals, tools and anonymisation of the CCHIC dataset have previously been described [27, 35] and the reader is referred to these references and references therein for a full description of the dataset. An anonymised dataset was retrieved for admissions between January 2015 and December 2016. Age, sex, admission APACHE-II score (we focus on APACHE II as other scoring systems are not included in CCHIC) as well as total daily adrenaline (epinephrine), noradrenaline (norepinephrine) and vasopressin, mechanical ventilation during the 24hour period, and daily minimum and maximum for: heart rate (HR), mean arterial pressure (MAP), ${\text{Pa}}_{{\text{O}}_{2}}$/${\text{Fi}}_{{\text{O}}_{2}}$ ratio, sodium, potassium, lactate, creatinine, CRP, and pH on each day for the first five days after ICU admission were used as the feature set. The outcome assessed was vital status at ICU discharge (survival at ICU discharge). Each ‘day’ is one of the consecutive 24 hour periods after admission; day 1, for example, begins at admission, and ends 24 hours after admission. This dataset was chosen to make our model as comparable to APACHE II as was possible given what is available within the CCHIC data catalogue. All ICU patients were included.

Each variable on each day was treated as a separate distribution for removal of outliers which were defined as values more than five standard deviations from the mean in most cases. For some variables, outliers were removed according to limits defined by clinical experience and visual inspection of the data (S1 Table). The data for ${\text{Pa}}_{{\text{O}}_{2}}$/${\text{Fi}}_{{\text{O}}_{2}}$ ratio appeared to be two overlaid distributions using different units and so was first transformed by placing an upper limit of 80kPa and dividing values above this by 7.6 to correct for measurements erroneously made in mmHg, before subsequently removing outliers. In total, 0.12% of all values were removed as outliers, with a maximum of 0.65% of values being removed from any one variable.

### Imputation of missing data

Missing data for noradrenaline, adrenaline and vasopressin was assumed to be missing-not-at-random, with most missing data points representing absence of drug administration. Missing data in these variables was therefore replaced with values of zero. Missing data for the remaining continuous variables was assumed to be missing-at-random. It was therefore multiply imputed by predictive mean matching using a parallel implementation of multivariate imputation by chained equations (MICE), using the parlMICE wrapper (https://github.com/gerkovink/parlMICE, accessed 15^th^ January 2018) for the R package ‘mice’ v2.46.0 [36]. This generated nine imputations which appeared consistently plausible on inspection of distributions (S1 Fig). The first imputation was taken forward for use in tuning of the machine learning models. All imputations were used to train and validate the tuned final version of each model, allowing for an estimation of the variability in prediction due to imputation.

### Modelling methodology

#### Model selection

Alongside logistic regression, several machine learning methods were used to train classification models. Aiming to achieve the best predictive model for each day, class representatives of several different machine learning methods were selected. Four machine learning models were implemented using the ‘caret’ package v6.0-77 [37]. ‘adaboost’ (‘AdaBoost.M1’), a boosted decision tree algorithm, was selected due to past performance with this dataset. ‘parRF’, a parallel implementation of a random forest algorithm, ‘svmRadialWeights’, a support vector machines algorithm with radial basis function kernel and class weights, and ‘avNNet’, a single layer model averaged neural network, were selected as good class representatives based on performance across multiple datasets [38]. These were implemented using the ‘caret’ package v6.0-77 [37]. Deep Learning was implemented using the ‘keras’ package v2.1.4.9000 for R to implement a six hidden-layer neural network with ‘TensorFlow’ [39, 40].

#### Predictor variables

On all days but the first, two models were built for each day, one using measurements from only that day, termed ‘simple’, and one using both measurements from that day and from all prior days, termed ‘cumulative’. The variables put into each model were all those named above, with the exception of APACHE-II score, duration of stay in ICU and vital status at discharge, the latter of which served as the outcome classifier. For comparative assessment with the APACHE-II score, a logistic regression was built for each day using the caret package ‘glm’ function with APACHE-II score as the single predictor. For each day, patients who had outcomes prior to the day of interest were removed before folding the data.

#### Cross-validation and pre-processing

Models built using caret were passed a reproducible list of pseudo-random seeds and run in parallel using the ‘doMC’ package v1.3.5. Deep learning models were passed a single set seed and run in parallel using the ‘parallel’ package v3.4.2.

Validation was performed by leave-group-out cross validation, in which the data was folded twenty times into 80% training data and 20% validation data. Models were trained on each of the twenty training sets and validated on the corresponding validation set. Prior to model training, the training sets were each normalised, first by power transformation with the Yeo-Johnson transformation [41], then centered to set the mean as zero and scaled to set the standard deviation as one. The validation sets were normalised by applying the power transformation, centering and scaling functions generated by the training sets. Folding and normalisation was all performed by the caret package and was consistent across all models; thus the 20 training and validation sets were identical for all models.

#### Hyperparameter optimisation

Machine learning models implemented using caret were first ‘tuned’ on twenty folds of one imputation, optimising hyperparameter values for maximal AUC, which was calculated using the pROC package v1.10.0 [42]. An initial iterative ranging investigation was undertaken to gain an understanding of a rough range of hyperparameter values to select, attempting several values and visually inspecting graphs of AUC against interactions of hyperparameter values, to determine the subsequent range of values to attempt. This informed a suitable initial grid across which a structured search was performed to optimise the hyperparameter values, though the incremental gains in AUC were typically small by this stage. After this initial grid search, visual inspection of graphs plotted was used to confirm reasonable coverage of the optimal level of each tuning parameter; indication of an optimal value outside of those used, or potential for gains from finer tuning within the grid, informed a second iteration of hyperparameter optimisation by structured grid search. The tuning was stopped at a maximum of two iterations of grid search in the interests of computing time, given gains in AUC were likely to be minimal.

Prior to optimisation of hyperparameters for the Deep Learning neural network, a similar ranging investigation was performed to determine a suitable depth, complexity and form of the neural network. Six layers was selected as a balance between allowing for sufficient complexity and avoiding over-fitting, with deeper neural networks tending to more readily over-fit the training data. A similar balance between complexity and over-fitting revealed a suitable first hidden layer size of around 128 nodes, with subsequent layers decreasing in size. These initial searches also revealed that the inclusion of at least one non-linear layer greatly improved performance. Approximate values for dropout rates in each layer and the number of epochs were determined in a similar process. These hyperparameter values were then optimised in the same way as the caret models, by passing a structured grid of values for each day to allow selection of a more optimal model structure. This grid altered the number of nodes in each layer (the relative size of each layer was constant), the activation of each layer (rectified linear unit, sigmoid or tanh), the dropout rate, and the number of epochs. Due to large number of adjustable parameters, potentially resulting in lengthy tuning grids, most of the hyperparameter search was limited to altering the first three layers of the network, which appeared to have a greater effect on the AUC than altering the last three layers. Two iterations of structured grid hyperparameter optimasation were performed for the Deep Learning models.

Logistic regression requires no hyperparameters but to optimize performance and reproduce some published interactions in sepsis, three potential two-way interactions were explicitly included [14]; interactions between lactate & noradrenaline, lactate & MAP and age & MAP were tested by including them in a logistic regression model with all other predictor variables on day one on the entire dataset. All possible interactions between minimum and maximum values were first included to asses which showed the largest effect. A second logistic regression was then performed including only the maximally important interactions, along with all other predictor variables for the first day (no correction for multiple testing was applied to the *p* values presented). This revealed the presence of significant interactions between lactate & noradrenaline, between lactate & MAP, and between age & MAP (see Results). These interactions were therefore included in the variables for the logistic regression models.

#### Assessment of final models for each method

The hyperparameters which gave the highest AUC were taken forward for each model for each day, simple and cumulative. These were then used to build a model for each of twenty folds of the nine imputed datasets to give isolated estimates of variability in AUC due to both folding and imputation. The variance due to folding and the variance due to imputation were added to give an overall estimate of the variability in model performance.

Discriminative ability, measured by AUC, was used as the sole measure of model performance used. As the machine learning models used were not intended for development into clinical tools, there is naturally no consideration of calibration or net reclassification index (NRI). Such considerations are only possible when the model is forced to predict particular binary outcomes for individual cases, requiring the setting of some threshold value above which a positive case is identified. The threshold value depends on the intended purpose of the prognostic tool, which informs the desired sensitivity and specificity. These considerations are beyond the scope of this study.

## Results

### Sample characteristics

**Fig 1:**
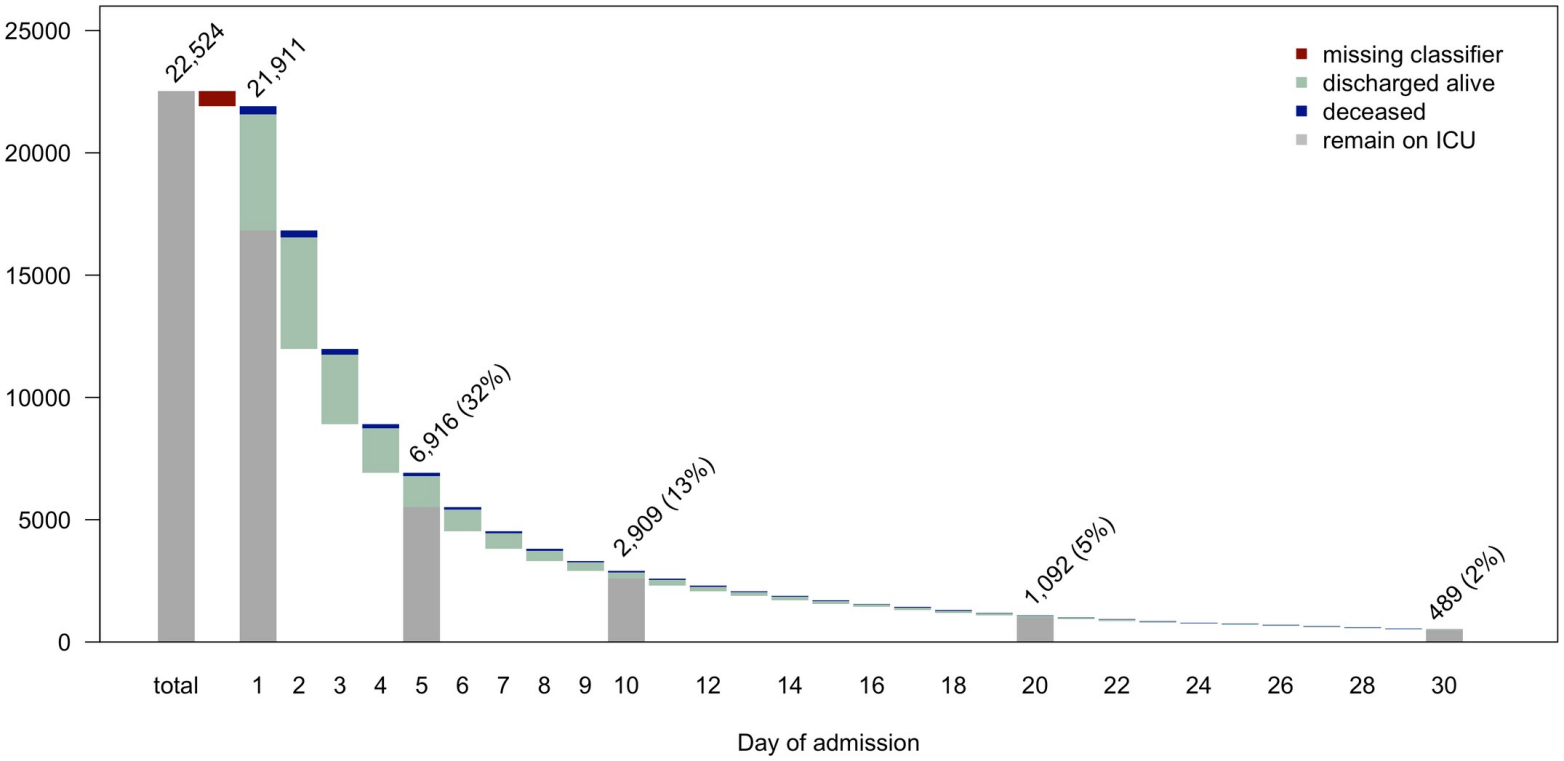
Admission duration waterfall plot. The total number of admissions in the patient database is shown, followed by the number removed due to a missing value for the classifier, leaving the number of admissions included in the analysis at day 1. On each day, bars show the number of patients discharged alive (light blue) or deceased (dark blue). The numbers above the bars represent the total number of patients remaining on intensive care at the start of the day and the percentage that this represents of all admissions included in the analysis, for days 1, 5, 10, 20 and 30. Where a total is shown, the grey bar represents those remaining on ICU at the end of the day.

The dataset retrieved from the CCHIC database contained information on 22,514 intensive care admissions. Of these, 613 lacked a value for the classifier and so were removed, leaving a sample of 21,911 admissions, of which 19,905 (90.8%) were alive at discharge. The distribution of length of admission and the proportions of patients deceased or discharged on each day can be seen in Fig 1. By day 30, 98% of admissions have finished, with the longest admission lasting 171 days. The descriptive statistics for each variable for each day are presented in Table 1. The distributions of each continuous variable for each day, split by vital status at discharge are presented in S1 Fig. Differences can be noted between those deceased on discharge and those alive in all variables, and the discriminating ability of the APACHE-II score can be seen clearly.

**Table 1:** Descriptive statistics of the ICU patient population over the first days after admission.

	Day 1	Day 2	Day 3	Day 4	Day 5
days remaining of admission	5.46 (8.60)	5.81 (9.41)	6.76 (10.54)	7.74 (11.58)	8.69 (12.49)
sex (male)	0.59	-	-	-	-
APACHE-II score	15.35 (6.39)	-	-	-	-
age /*years*	61.37 (17.27)	-	-	-	-
ventilated (yes)	0.40	0.29	0.32	0.35	0.38
min HR /*bpm*	71.2 (16.14)	74.37 (15.5)	75.44 (15.58)	75.5 (16)	75.55 (15.96)
max HR /*bpm*	101.62 (21.36)	100.45 (20.81)	101.84 (21)	101.96 (20.75)	101.84 (20.58)
min MAP /*mmHg*	64.82 (12.51)	68.25 (13.13)	69.29 (13.78)	69.87 (14.14)	70.14 (15.09)
max MAP /*mmHg*	102.41 (19.61)	100.66 (19.65)	102.55 (20.26)	104.07 (20.7)	104.52 (21.05)
min ${\text{Pa}}_{O_{2}}$/${\text{Fi}}_{O_{2}}$ /*kPa*	33.77 (14.99)	33 (13.79)	31.3 (13.33)	30.73 (13.05)	30.37 (12.84)
max ${\text{Pa}}_{O_{2}}$/${\text{Fi}}_{O_{2}}$ /*kPa*	48.24 (18.98)	43.62 (15.6)	41.83 (15.28)	41.27 (15.07)	40.9 (14.99)
min Lactate /*mmol*/*l*	1.31 (1.35)	1.22 (1.21)	1.18 (1.12)	1.16 (1.05)	1.16 (0.98)
max Lactate /*mmol*/*l*	2.80 (2.65)	2.18 (2.01)	2.00 (1.78)	1.93 (1.71)	1.91 (1.56)
min Sodium /*mmol*/*l*	136.56 (5.03)	137.15 (5.09)	137.69 (5.37)	138.33 (5.49)	138.85 (5.64)
max Sodium /*mmol*/*l*	140.49 (4.93)	140.13 (5.24)	140.52 (5.55)	141.13 (5.78)	141.65 (5.94)
min Potassium /*mmol*/*l*	3.93 (0.58)	4.01 (0.48)	4.01 (0.47)	4.02 (0.45)	4.05 (0.46)
max Potassium /*mmol*/*l*	4.85 (0.74)	4.66 (0.57)	4.63 (0.54)	4.64 (0.54)	4.67 (0.54)
min PH /*pH*	7.32 (0.09)	7.36 (0.07)	7.37 (0.07)	7.38 (0.07)	7.38 (0.07)
max PH /*pH*	7.42 (0.06)	7.43 (0.06)	7.43 (0.06)	7.44 (0.05)	7.44 (0.06)
min Creatinine /*μmol*/*l*	118.51 (109.32)	117.38 (96.47)	111.72 (88.75)	108.13 (82.51)	107.16 (80.39)
max Creatinine /*μmol*/*l*	136.05 (136.13)	121.51 (102.74)	114.55 (92.93)	110.5 (85.73)	109.53 (84.09)
min CRP /*mg*/*l*	77.34 (91.38)	118.91 (91.8)	126.79 (94.95)	116.95 (91.73)	106.19 (88.03)
max CRP /*mg*/*l*	115.21 (104.85)	155.1 (110.32)	157.29 (109.24)	144.74 (104.9)	131.32 (100.82)
noradrenaline /*μg*/*kg*/24*h*	84.0 (168.0)	81.6 (177.7)	81.8 (161.8)	77.8 (148.1)	79.2 (145.8)
adrenaline /*μg*/*kg*/24*h*	64.8 (126.0)	135.0 (288.4)	108.1 (230.9)	120.1 (131.4)	102.9 (109.3)
vasopressin /*units*/24*h*	777.0 (1327.5)	778.8 (1767.0)	818.4 (2102.4)	900.0 (1560.0)	540.0 (1899.6)

Figures represent arithmetic mean (standard deviation) for numerical variables and proportions for all binomial variables, with the exception of the drugs for which median (inter-quartile range) is shown. All values are calculated from all non-missing data, after removal of outliers, and before imputation of missing data.

### Replication of simple interactions

Testing of interactions in mortality prediction by logistic regression for all possible combinations of lactate & MAP, age & MAP, and lactate & noradrenaline in the first day revealed the strongest interactions between minLactate:noradrenaline, minLactate:minMAP, and age:maxMAP. Removing the likely co-linear interactions (e.g. age:minMAP, maxLactate:minMAP) before performing a logistic regression which included these three interactions alongside all other covariates revealed significant interactions between minLactate:noradrenaline (*p* = 2.54 × 10^−12^), minLactate:minMAP (*p* = 5.96 × 10^−5^), and age:maxMAP (*p* = 1.40 × 10^−2^). These interactions were subsequently included in the logistic regression models for all days.

### Variability due to imputation

Multiple imputation produced nine versions of the complete dataset to assess variability in imputation. The distributions of imputed data for each variable appeared plausible on inspection of density plots (S2 Fig). The variability in model performance due to imputation was small in comparison to the variability due to folding of the data; the variance of AUC across imputations ranged from 0.09% to 16% of the variance of AUC across folds, with a mean of 6.2% confirming that the imputation was stable. The two sources of variance were added for each model when calculating overall variance for model AUC.

### Performance of models on the first day

**Fig 2:**
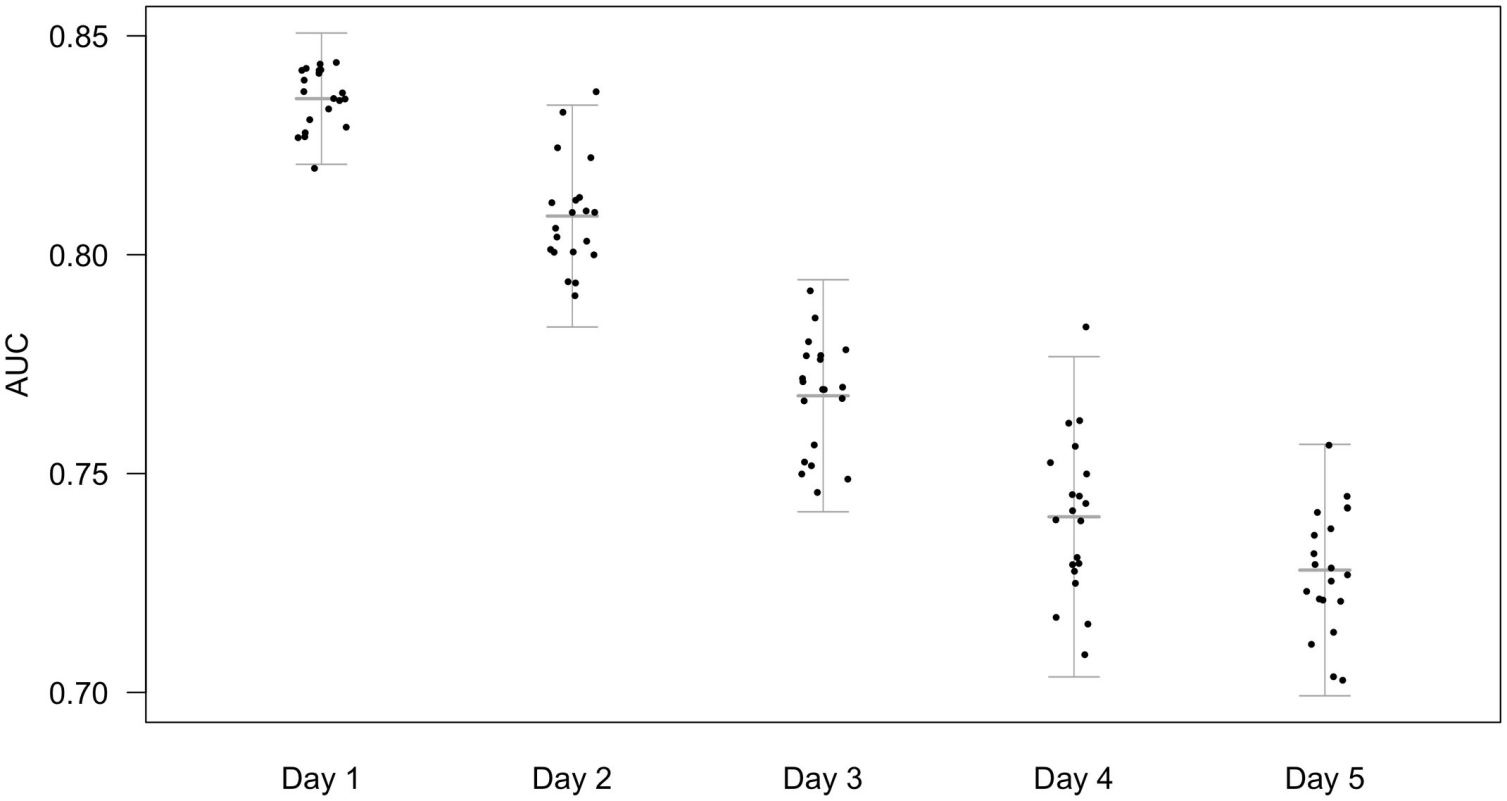
AUC of APACHE-II when applied to patients remaining in ICU on days 1-5. Area under the receiver operating characteristic curve (AUC) from logistic regression models built applying admission (24 hour) APACHE-II for only the patients remaining on each subsequent day, predicting vital status at discharge on twenty cross-folded validation sets. Points represent the mean AUC for each fold across nine imputations. Bars represent the mean of twenty folds +/- 2 standard deviations, calculated from the combined variance of folding and imputation. The predictive performance of admission APACHE-II declines when applied to predict outcome on subsequent days.

Fig 2 shows the predictive power for the APACHE-II score alone as a baseline which is a good predictor on day 1, with an AUC in this dataset of 0.836 (*σ* = 0.007). The discriminative power of the APACHE-II score to predict outcomes on subsequent days was seen to reduce considerably (Fig 2).

**Fig 3:**
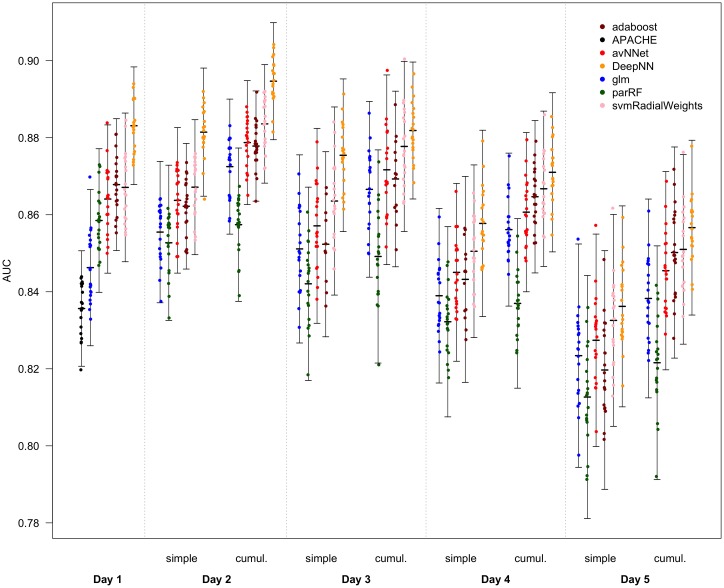
AUC of logistic regression and machine learning models for each day. Area under the receiver operating characteristic curve (AUC) for predictions of vital status at discharge in twenty cross-folded validation sets for models built on each day. On the first day, the ‘APACHE’ model is a single predictor logistic regression model built from the APACHE-II score. The first and all subsequent days show AUCs of logistic regression (‘glm’), random forest (‘parRF’), a boosted decision tree algorithm (‘adaboost’), a single layer model averaged neural network (‘avNNet’), a support vector machines algorithm with radial basis function kernel and class weights (‘svmRadialWeights’), and a six hidden-layer deep learning neural network (‘DeepNN’). ‘Simple’ models use only measurements from one day as predictors. ‘Cumulative’ (cumul.) models use measurements from the day and preceding days as predictors. Points represent the mean AUC for each fold across nine imputations. Bars represent the mean of twenty folds +/- 2 standard deviations, calculated from the combined variance of folding and imputation.

Logistic regression from the variables selected on day 1, including specified interactions, had an AUC of 0.846 (*σ* = 0.010) (Fig 3), which slightly outperforms the APACHE-II score result even though our model does not contain the chronic disease status. However the machine learning models trained on day 1 data all outperformed even this: parRF AUC 0.859 (*σ* = 0.009), avNNet AUC 0.864 (*σ* = 0.010), svmRadialWeights AUC 0.867 (*σ* = 0.010), adaboost AUC 0.868 (*σ* = 0.008), Deep Learning AUC 0.883 (*σ* = 0.008). On all days, machine learning consistently outperformed logistic regression (Fig 3; Table 2).

**Table 2:** AUC of models on each day, simple and cumulative.

	Day 1	Day 2		Day 3		Day 4		Day 5	
	Simple	Simple	Cumulative	Simple	Cumulative	Simple	Cumulative	Simple	Cumulative
APACHE	0.836 (0.007)	0.809 (0.013)		0.768 (0.013)		0.740 (0.018)		0.728 (0.014)	
glm	0.846 (0.010)	0.855 (0.009)	0.872 (0.009)	0.851 (0.012)	0.867 (0.011)	0.839 (0.011)	0.856 (0.010)	0.823 (0.014)	0.838 (0.013)
parRF	0.859 (0.009)	0.853 (0.010)	0.857 (0.010)	0.842 (0.012)	0.849 (0.014)	0.832 (0.012)	0.837 (0.011)	0.813 (0.016)	0.822 (0.015)
avNNet	0.864 (0.010)	0.864 (0.009)	0.879 (0.008)	0.857 (0.013)	0.872 (0.012)	0.845 (0.011)	0.861 (0.010)	0.827 (0.014)	0.845 (0.013)
adaboost	0.868 (0.008)	0.862 (0.008)	0.879 (0.006)	0.853 (0.012)	0.871 (0.012)	0.843 (0.013)	0.865 (0.010)	0.820 (0.015)	0.850 (0.014)
svmRadialWeights	0.867 (0.010)	0.849 (0.081)	0.884 (0.008)	0.864 (0.012)	0.878 (0.011)	0.851 (0.011)	0.867 (0.010)	0.833 (0.014)	0.851 (0.012)
DeepNN	0.883 (0.008)	0.881 (0.008)	0.895 (0.008)	0.875 (0.010)	0.882 (0.009)	0.858 (0.012)	0.871 (0.010)	0.836 (0.013)	0.857 (0.011)

Values represent mean (standard deviation) area under the receiver operating characteristic curve across nine imputations of twenty folds for each method for each day. These values correspond to Fig 3.

### Temporal determinants of prediction

When updating the predictors to include data for each day, a clear trend is seen. From the third day, predictive ability of all models deteriorates, with inclusion of trend information mitigating this decline to some extent. However, the second day is different. Using the same predictor variables as the first day, but using values from the second day (i.e. day 2 simple), results in similar AUCs to the first day for most methods. However, when including values from the first day also, and thus considering trend information, AUC is greatly improved (Fig 3. This trend is seen in both logistic regression and all machine learning methods, with a maximum AUC on day 2 of 0.895 (*σ* = 0.008) for Deep Learning.

**Fig 4:**
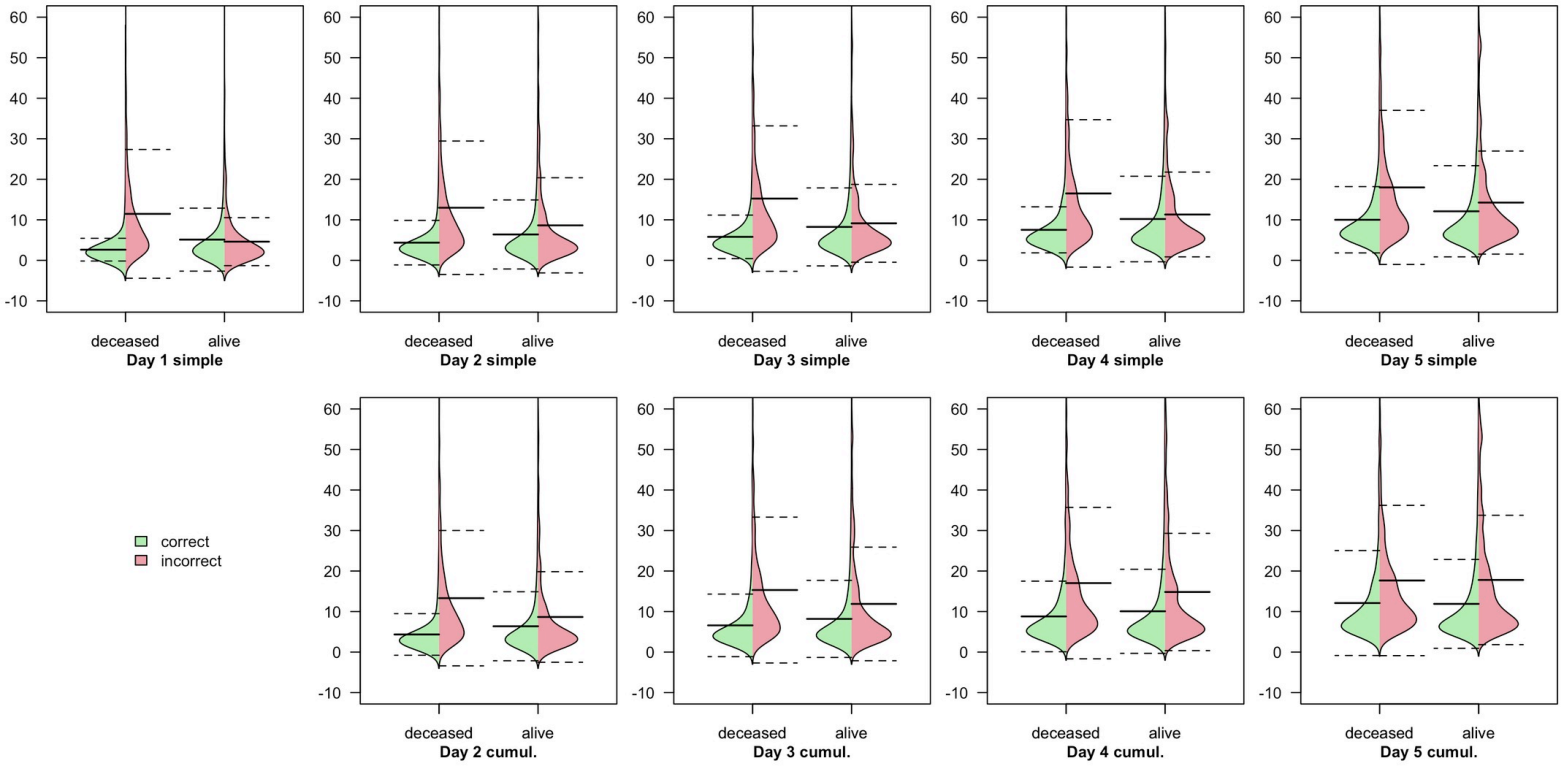
Distributions of length of admission for patients correctly and incorrectly classified as alive or deceased by the Deep Learning classifiers. Each plot represents the four smoothed distributions of correct/incorrect prediction split by actual outcome (alive/deceased) for each Deep Learning classifier on each day. Distributions of admission duration for correct predictions are shown in green, while those for incorrect predictions are shown in red. Thick lines represent arithmetic mean, with dashed lines indicating mean +/- standard deviation.

Examination of the predictions made by the Deep Learning classifiers on each day, revealed that the length of admission remaining affected the ability of the model to predict outcomes, particularly deaths (Fig 4).

## Discussion

### Temporal determinants of prediction

The ability to predict outcomes in intensive care is determined by the time at which the prediction is made. From simple demographic, physiological and treatment parameters, outcomes can be predicted more accurately on the second day of intensive care admission than on the first, but this ability then begins to deteriorate (Fig 3). This has significant implications for decision making in and after the first hours of intensive care admission. Firstly, it follows that maintaining patients in intensive care for more than one day carries a prognostic advantage. Secondly, maintaining admissions beyond a second day does not confer a prognostic advantage. Thirdly, it follows that for those patients who remain in intensive care for a second day, consideration of the trend in physiological variables is required for optimal assessment of prognosis. From the CCHIC database, roughly 75% of admissions last for more than one day. This result provides support for the practice of time-limited trials of intensive care, showing that this strategy can improve prognostic ability. There may also be a place for a new prognostic clinical tool which can update predictions based on the new information provided by the second day.

The CCHIC ICUs may not be wholly representative of intensive care provision across the UK. However, the size of the CCHIC dataset used in this study, its multi-center nature, and the inclusion of all intensive care admissions would all be expected to improve the generalisability of these results to UK practice. Models developed in one geographical region or healthcare system tend show inferior calibration when applied to other regions, but typically retain good discriminative ability [43–45]. Discrimination, measured here by AUC, is the metric of interest in these models, to ascertain the maximum predictive ability; with no intention of generating a binary outcome prognostic clinical tool, there is no concern for calibration. Furthermore, a temporal trend in predictive ability is a coarse, and presumably general feature of the critically ill which could therefore be expected to apply to any ICU worldwide. Despite this, validation of this trend in other regions would be informative.

### The advantage of machine learning

That the logistic regression (‘glm’) model constructed from our dataset outperforms APACHE-II is interesting. Unlike APACHE-II, our dataset did not include any chronic or disease variables illustrating the clinically rich information content of the physiological / laboratory data alone in an appropriate model (Fig 3).

The out-performance by machine learning methods shows that outcome in intensive care is partly a function of complex interactions and non-linearities in these physiological variables. Machine learning methods are able to exploit these in outcome classification, and show substantially better performance than linear models, with the exception in this dataset of parRF, a random forest algorithm. Deep Learning, which has the advantage of being able to uncover even more complex interactions than other machine learning algorithms, has the best performance on this dataset, despite the relatively small size of multilayer perceptron model used here (Fig 3). In fact, the Deep Learning model used here is relatively ‘shallow’, with six hidden layers, and the largest layers containing around 100-150 nodes; a larger dataset might allow deeper neural networks to uncover more complexity in the data without over-fitting.

When using all available variables, the predictive ability of Deep Learning on day 2 is excellent, particularly considering the relatively few variables included and the absence of non-physiological variables often included in more modern intensive care scoring systems (e.g. diagnosis). Due to limited time and resources, the models, particularly Deep Learning, did not undergo more than initial tuning and optimization and could improve further even on this dataset. Despite all of this, the AUCs shown by the Deep Learning models are remarkable. AUCs of 0.883 (*σ* = 0.008) and 0.895 (*σ* = 0.008) for days 1 and 2 are substantially higher than AUCs reported in the literature for most intensive care mortality prediction tools, with the exception of APACHE-IV which has shown AUCs of around 0.89 when re-calibrated to the local ICU population [20–22].

One possible explanation for improved performance over time is that the admission cohort is enriched with patients who are somehow inherently unpredictable. However this does not seem to be the case from Fig 2 as the performance of the APACHE-II classifier falls over time rather than demonstrating a similar rise on day 2/3.

### Deep Learning in the ICU

Deep Learning does not naturally lend itself to the development of practical clinical tools, due to the ‘black box’ effect; the complexity of the model means that analysis and explanation of how the covariates are utilised is limited. Techniques do exist, but a formal evaluation of the workings of the neural networks here is beyond the scope of this study. However, some information can be readily gleaned from an investigation into which patients are correctly and incorrectly classified by the model.

Fig 4 shows that the Deep Learning classifier, which as the most accurate classifier, serving as a proxy for information content in the data, is less successful at predicting outcomes which will occur further in the future, particularly deaths. This suggests that some information is missing which would help to differentiate those patients who will survive for some time before dying. The absence of any covariates for chronic health status means that it may be plausible to assume that outside of the subsequent few days, chronic health factors begin to gain more importance in mortality prediction than physiological and laboratory values.

Machine learning approaches and Deep Learning in particular are complex algorithms that are inherently less interpretable than techniques such as logistic regression. However, in this work we seek to use such approaches to see what additional information content is unexplained by traditional classifiers and to examine the time variation of this rather than understand the causative factors so this is less of a consideration for our work. The increase on day 2 from 0.872 to 0.895 may seem marginal, however an AUC is approached only asymptotically, such an apparently small increase in fact represents a very large increase in model performance at these levels. Indeed that an AUC of approaching 0.9 can be achieved is, in the authors’ opinion, remarkable in itself given the nature of the data.

Despite the difficulties in translation to a useful clinical tool, Deep Learning has shown promise in improving the quality of predictions. In this study we aim to evaluate whether there is predictive information that is not captured by conventional time-insensitive models and machine learning techniques are a natural tool for this. We do not set out to develop a new prognostic clinical tool. However our results do suggest that such a tool could be developed using machine learning, and provide evidence that it is likely to succeed beyond current models. However the development of such a classifier would require consideration of (additional) optimal features, optimal sampling as well as a demonstration of appropriate calibration performance which is beyond the scope of this work.

### Explicitly defined interactions in logistic regression

It is worth noting that the interactions shown in the logistic regression models validate previously suggested interactions. Shankar-Hari et al. showed significant interactions between blood pressure, lactate and vasopressor therapy in mortality prediction [14]. In testing interactions for logistic regression models, we have shown that these interactions are significant across the intensive care population as a whole.

### Limitations

There are some limitations to this study which have been mitigated, but not entirely avoided. While the data is of a good quality for a clinical dataset, it has drawbacks. The proportion of missing data is high for some variables (S2 Table). To preserve the sample size for the analysis, this missing data was imputed. To asses the impact of imputing this missing data on the performance of the models, several imputations were performed, subsequent distributions visualised to ensure plausibility, and the variability between performance of models trained on them measured and included in the estimates of overall prediction variability. The ${\text{Pa}}_{{\text{O}}_{2}}$/${\text{Fi}}_{{\text{O}}_{2}}$ ratio has been measured in two different units, producing overlapping distributions; the transformation applied mitigates this but the overlap means some values will be wrong, which has implications on all variables due the the effects of imputation.

The size of the dataset is, naturally, not consistent across all days, decreasing with increasing length of admission. While there are 21,911 patients on day 1, there are just 6,916 remaining on day 5. Conversely, the number of variables included in these models increases. This is seen as a small increase in variance with time in Fig 2. Consequently, the predictive models for later days have a higher risk of over-fitting the training sets, thus limiting predictive power for the validation sets. This is mitigated by passing a range of tuning parameters to the models which allows different model structures and complexities across the days. Over-fitting appears to have been avoided, given the superior performance of cumulative models on day 5 despite the greater number of predictor variables included. However, the reduction in maximum complexity of the models may be an intrinsic bias against performance in the later days. This can be seen in the reduction of advantage of the machine learning methods over logistic regression in the later days (Fig 3).

Our dataset is somewhat unbalanced due to the smaller number of deaths than survival. We have investigated re-weighting our training data but resulted in worse performance in the test set suggesting that the imbalance is not so great as to have an impact on classifier performance in any important way. It is noteworthy that the mortality is similar over the time-period studied so the imbalance is at least constant.

One motivation for understanding whether prognostication is time-sensitive is the concept of time-limited trials of intensive care. Of course, it is important to note that our dataset includes ICU all-comers and is therefore not representative of the type of patients in whom such a trial would be considered. Indeed such a dataset would be difficult to amass since such cases represent a small proportion of the ICU population. Nevertheless, the general point that prognostic power varies with time, and that there may be a benefit from prognosticating at a time-point later than day one is still valid.

Of course, mortality is not the sole outcome on which to base clinical decisions in the ICU. However, it serves as a proxy, readily available, binary outcome which makes it useful for this initial investigation into temporal determinants of prognosis. Our demonstration that a very high performance classifier can be created for this simpler outcome is reassuring since multidimensional and multi-class outcome assessment will require very complex models and possibility of modelling the very complex relationships likely to be involved using ML is very attractive and motivates future research. We do not claim that simply because information content within the specific set of parameters that we have considered is higher on day 2 or 3 has direct translation into defining an optimum duration of a time-limited trial. However at present practice varies widely [46] and there has been little attempt to study this robustly. The novelty of our work is that it demonstrates that prognostication may be improved if delayed and this is an important pre-requisite if time-limited trials are to make sense at all. A more comprehensive study of this would require an enriched dataset both in terms of patient illness severity but also in terms of other social, ethical and outcome determinants.

We have deliberately constrained our work to early prediction. It is interesting that we have achieved very impressive classification accuracy with only the physiological variables available, without including the chronic health status of the patient. This suggests that mortality at different times may depend on different features. It is highly likely, for example, that late ICU deaths may instead be dominated not by early physiology / acute disease severity but instead by chronic health status and physical reserve. We did not have the data to investigate this, but it would be an interesting area to examine in the future.

It is worth remaining mindful that our dataset is an observational one. For this reason, it will contain biases due to systematic treatment decisions which are not captured by the physiological measurements and any ML model based on such data will inherently learn such biases also. Our assumption of missing-at-random on which imputation is necessarily based is open to challenge. These are a common and fundamental issue with observational research. Furthermore, it is well known that prognostic models do not necessarily generalise externally. However CCHIC is currently the largest heterogenous / multicentre dataset available and furthermore, it seems clinically implausible that time-varying information content or the additional predictive power of including trend information is entirely due to patient selection resulting from systematic treatment decision-making behaviour in the first days after admission.

## Conclusion

ICU prognostication is important for decision making and performance evaluation, yet commonly used systems rely on admission clinical parameters. We have demonstrated for the first time that intensive care prognostic performance is not static, varying even over the first days of admission and can be augmented by taking into account the change in physiological parameters over the course of several days. A prognostic advantage can be gleaned from prognostication after the first day with optimal information content being present around day 2.

ICU admissions are highly data-dense and improvements in data collection and the advent of large datasets have opened up new opportunities for optimal prognostication. The human being is a complex biological system and prognostication of even a major binary outcome requires consideration of this complexity which may be difficult to capture with linear techniques. Machine learning algorithms enable us to build optimal classifiers and thus may be used as a tool to investigate the information content in the available data.

## Supporting information

S1 Table Limits placed on variables for removal of outliers. (PDF)

S2 Table Proportions of missing data. Proportion of missing data for each variable.
(PDF)

S1 Fig Distribution of variables for each outcome. ‘Beanplots’ showing the distribution of each variable on each day, split by outcome.
(PDF)

S2 Fig Density plots of imputed missing data compared to original data. Comparison of the distributions of the imputed data and the original data showing plausibility of imputations.
(PDF)

S1 Text R code. The R code used to perform this study.
(PDF)

## Abbreviations

APACHE: Acute Physiology And Chronic Health Evaluation. A series of clinical tools used for mortality prediction in intensive care.
AUC: Area under the receiver operating characteristic curve. A measure of the discriminative ability of a classification model.
ICU: Intensive care unit.
ML: Machine learning.
CCHIC: Critical Care Health Informatics Collaborative
