# Supplementary material for: Optimal intensive care outcome prediction over time using machine learning
Source: PLoS One. 2018 Nov 14;13(11):e0206862. doi: 10.1371/journal.pone.0206862 (PMC6241126; doi:10.1371/journal.pone.0206862)
Supplement: S1 Table — (PDF) [file pone.0206862.s001.pdf]

S1 Table: Limits placed on variables for removal of outliers.

Variable	Lower limit	Upper limit	Data removed /%
HR	mean - 5SD	mean + 5SD	0.01
MAP	mean - 5SD	mean + 5SD	0.12
Pa_{O_2}/Fi_{O_2}	mean - 5SD	mean + 5SD	0.12
Sodium	mean - 5SD	mean + 5SD	0.19
Potassium	mean - 5SD	10mmol/l	0.08
Lactate	none	20mmol/l	0.02
creatinine	none	mean + 5SD	0.65
pH	mean - 5SD	mean + 5SD	0.24

SD: standard deviation. Values below lower limit and above upper limit are removed and later filled by imputation. Variables not in this table did not have outliers removed.
