# Supplementary material for: Optimal intensive care outcome prediction over time using machine learning
Source: PLoS One. 2018 Nov 14;13(11):e0206862. doi: 10.1371/journal.pone.0206862 (PMC6241126; doi:10.1371/journal.pone.0206862)
Supplement: S2 Table — Proportion of missing data for each variable. (PDF) [file pone.0206862.s002.pdf]

**S2 Table: Proportions of missing data**

| Variable | Missing data /% |
|---|---|
| VasopressinTotal | 99.25* |
| AdrenalineTotal | 99.04* |
| NoradrenalineTotal | 77.28* |
| $Pa_{O_2}/Fi_{O_2}$ | 51.41 |
| Age | 41.03 |
| CRP | 34.68 |
| PH | 34.05 |
| Lactate | 31.96 |
| Creatinine | 31.46 |
| HR | 22.93 |
| Sodium | 18.71 |
| Potassium | 18.59 |
| MAP | 11.88 |
| APACHE-II score | 6.30 |
| Sex | 0.00 |
| Duration of admission | 0.00 |
| Ventilated | 0.00 |
| Vital staus at discharge | 0.00 |

Percentage of missing data for each variable after outliers have been removed. Values marked with * assumed to be missing-not-at-random, with missing values representing values of zero. Otherwise, missing data was assumed to be missing-at-random and subsequently imputed.
