# Supplementary material for: Optimal intensive care outcome prediction over time using machine learning
Source: PLoS One. 2018 Nov 14;13(11):e0206862. doi: 10.1371/journal.pone.0206862 (PMC6241126; doi:10.1371/journal.pone.0206862)
Supplement: S1 Fig — ‘Beanplots’ showing the distribution of each variable on each day, split by outcome. (PDF) [file pone.0206862.s003.pdf]

S1 Fig: Distribution of variables for each outcome.

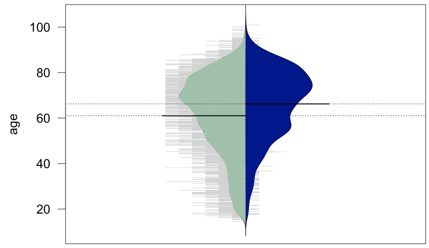

(a) age

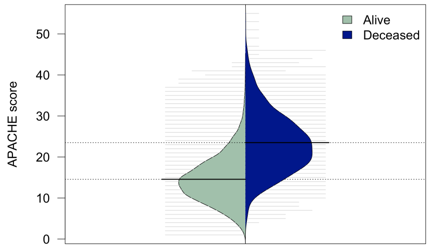

(b) APACHE-II score

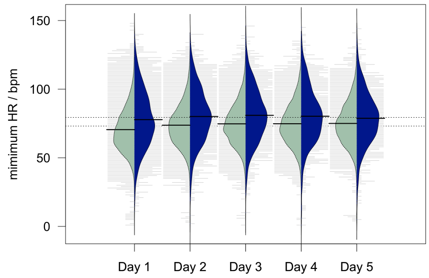

(c) Mimimum HR

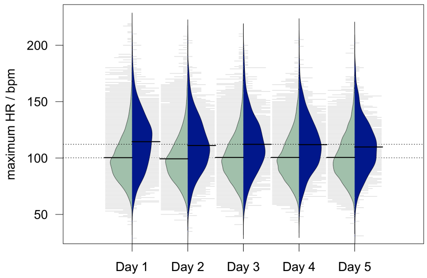

(d) Maximum HR

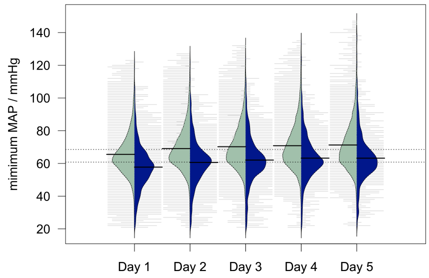

(e) Minimum MAP

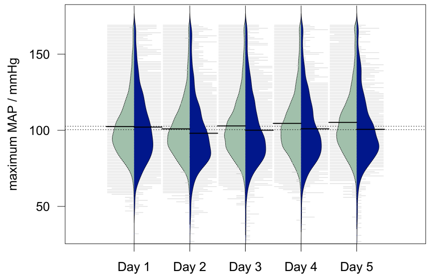

(f) Maximum MAP

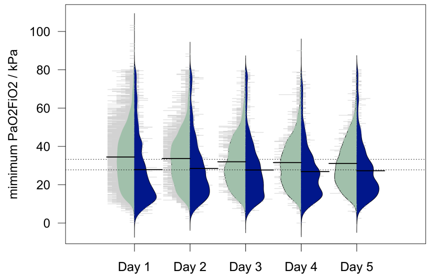

(g) Minimum $Pa_{O_2}Fi_{O_2}$

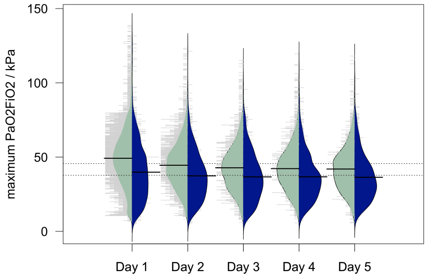

(h) Maximum $Pa_{O_2}Fi_{O_2}$

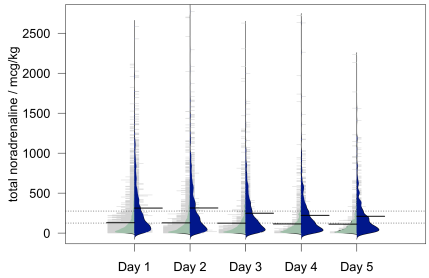

(i) Total noradrenaline

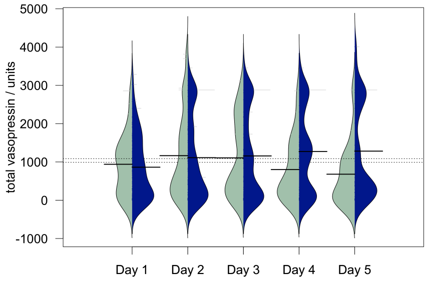

(j) Total vasopressin

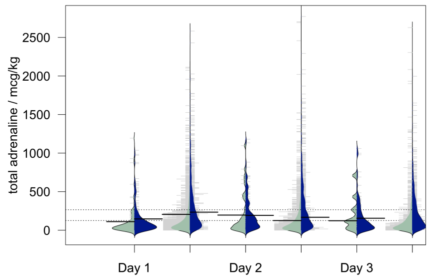

(k) Total adrenaline

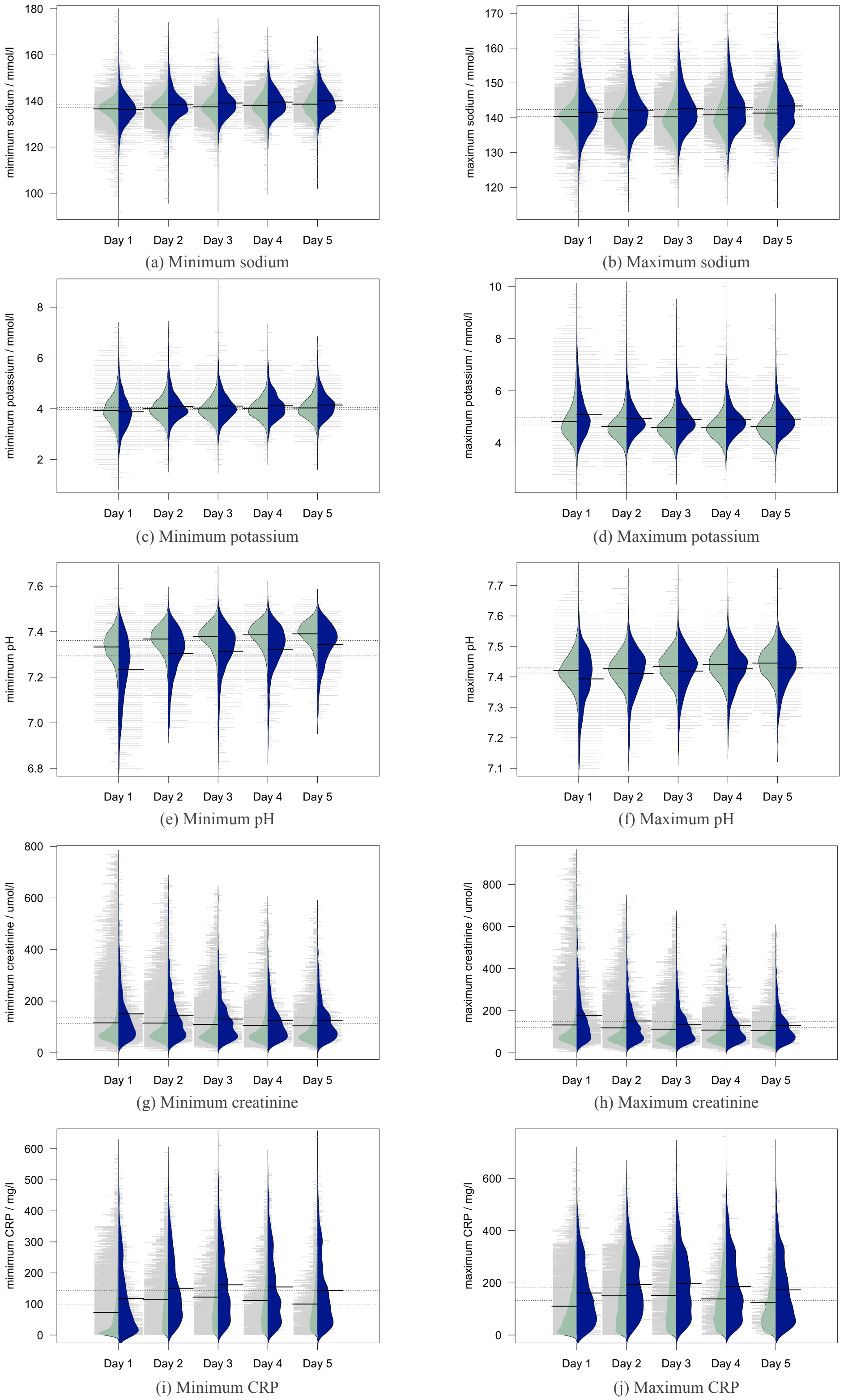

(a) Minimum sodium
(b) Maximum sodium
(c) Minimum potassium
(d) Maximum potassium
(e) Minimum pH
(f) Maximum pH
(g) Minimum creatinine
(h) Maximum creatinine
(i) Minimum CRP
(j) Maximum CRP

Baseline characteristics of ICU patient population, split by vital status at discharge, for all continuous variables. Beanplots show the distribution for each variable for each day, split into those alive at discharge (light blue) and those deceased at discharge (dark blue). Grey bars indicate individual values, thick black lines represent the means of each day and full-length horizontal lines represent the overall mean of those alive and deceased at discharge across all days.
