# Supplementary material for: Optimal intensive care outcome prediction over time using machine learning
Source: PLoS One. 2018 Nov 14;13(11):e0206862. doi: 10.1371/journal.pone.0206862 (PMC6241126; doi:10.1371/journal.pone.0206862)
Supplement: S2 Fig — Comparison of the distributions of the imputed data and the original data showing plausibility of imputations. (PDF) [file pone.0206862.s004.pdf]

S2 Fig: Density plots of imputed missing data compared to original data.

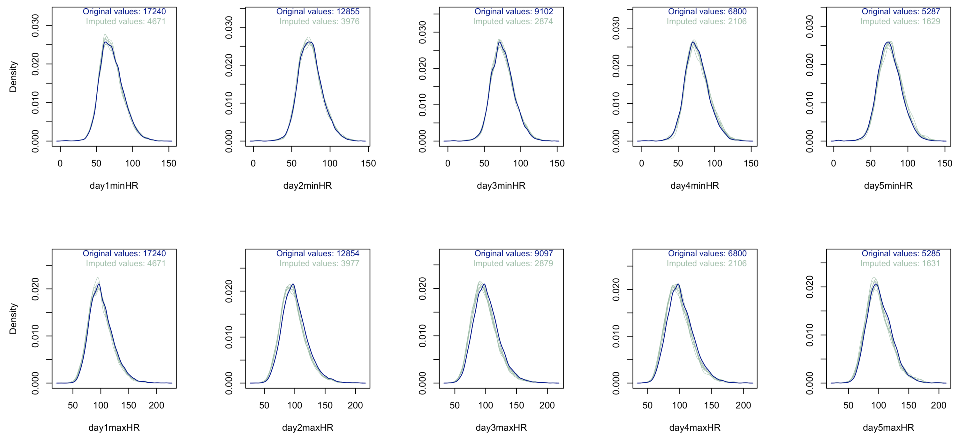

(a) HR

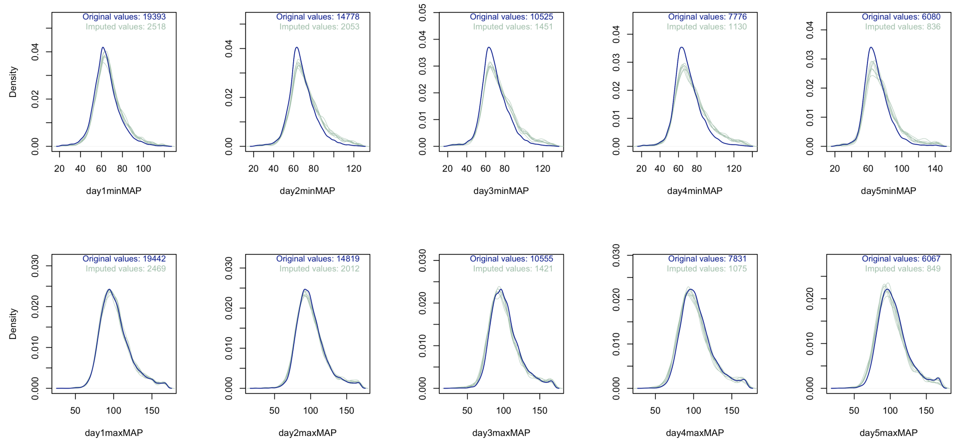

(b) MAP

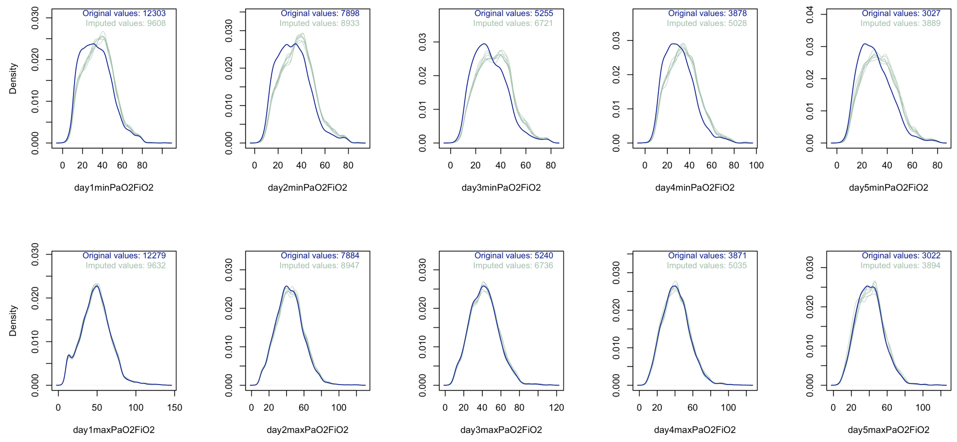

(c) $Pa_{O2}Fi_{O2}$

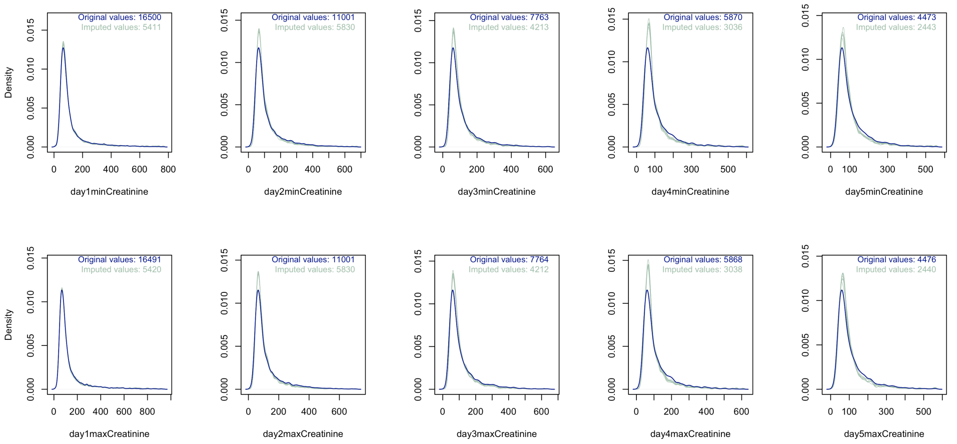

(d) Creatinine

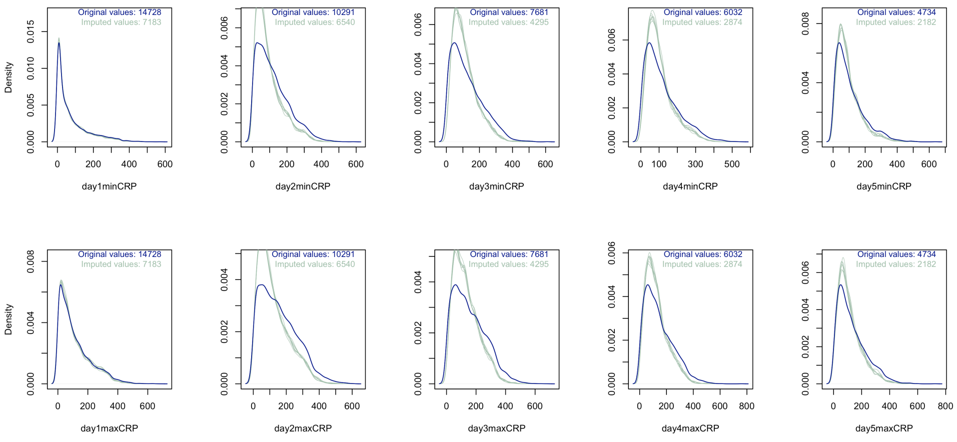

(e) CRP

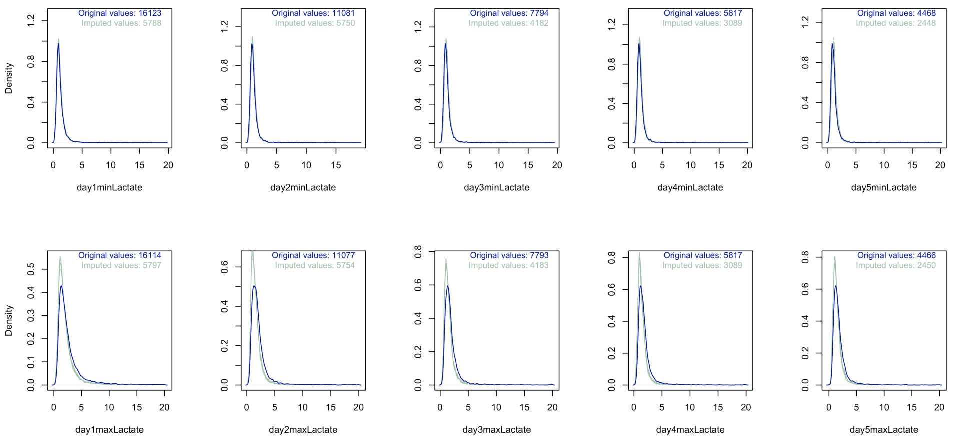

(f) Lactate

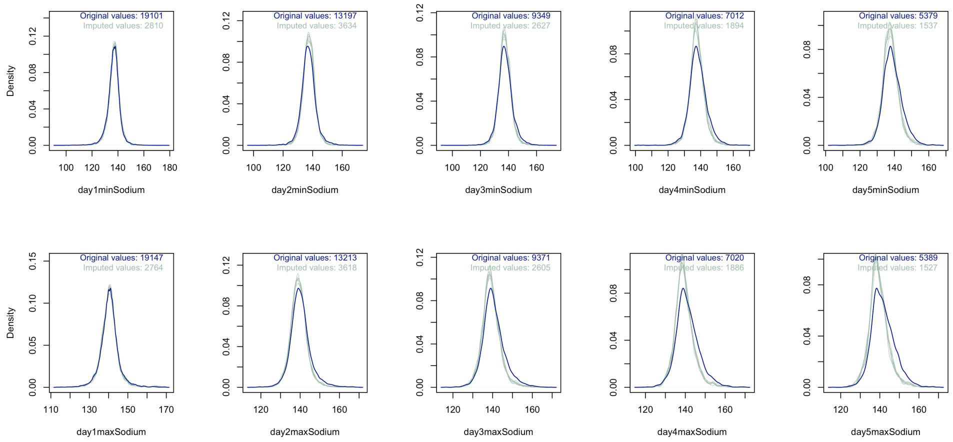

(g) Sodium

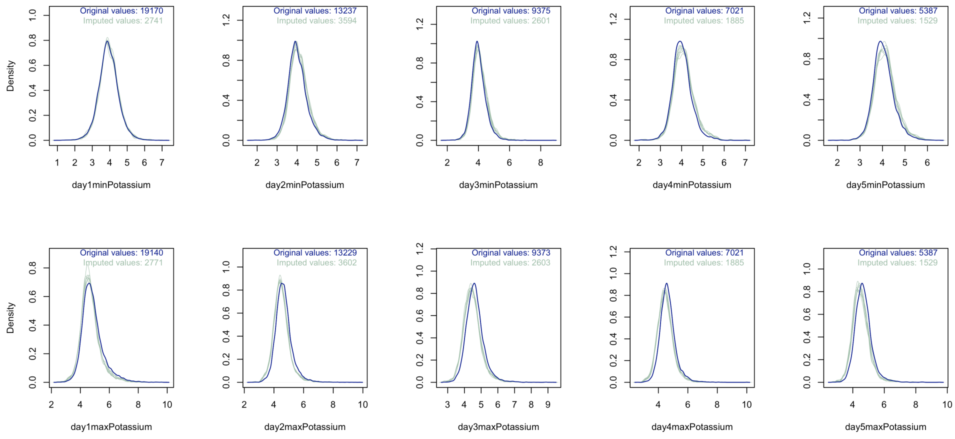

(h) Potassium

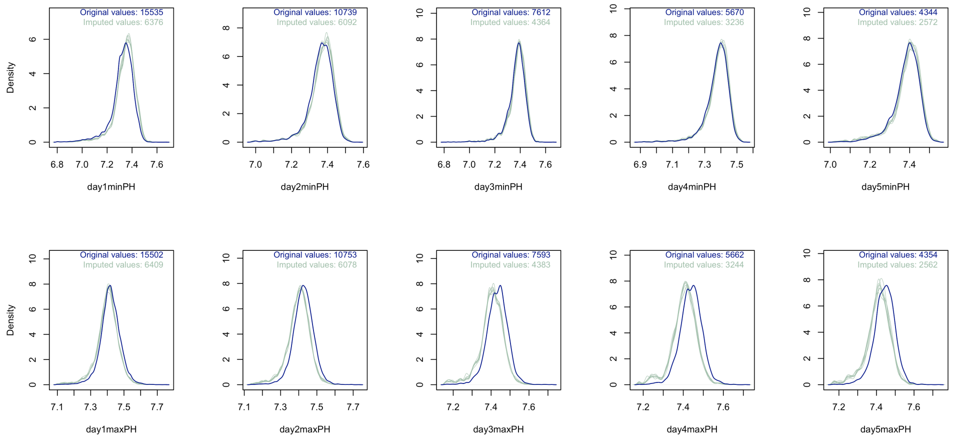

(i) pH

Density plots of the original cleaned data are in dark blue and the nine imputations of the missing data are represented in light blue.
