# Supplementary material for: Optimal intensive care outcome prediction over time using machine learning
Source: PLoS One. 2018 Nov 14;13(11):e0206862. doi: 10.1371/journal.pone.0206862 (PMC6241126; doi:10.1371/journal.pone.0206862)
Supplement: S1 Text — The R code used to perform this study. (PDF) [file pone.0206862.s005.pdf]

Optimal intensive care outcome prediction over time usingmachine learning

S1 Text. R Code in full.

C. J. Meiring[1], A. Dixit[1], S. Harris[2], N. S. MacCallum[2], D. A. Brealey[2], P. J. Watkinson[3], A. Jones[4], S. Ashworth[5], R. Beale[4], S. J. Brett[5], M. Singer[2], A. Ercole[1,‡,*]

1 Division of Anaesthesia, University of Cambridge, Cambridge, United Kingdom.
2 Bloomsbury Institute of Intensive Care Medicine, University College London, London, United Kingdom.
3 Nuffield Department of Clinical Neurosciences, University of Oxford, John Radcliffe Hospital, Oxford, United Kingdom.
4 Department of Intensive Care, Guy's and St Thomas' NHS Foundation Trust, St Thomas' Hospital, Westminster Bridge Road, Lambeth, London.
5 Centre for Perioperative Medicine and Critical Care Research, Imperial College Healthcare NHS Trust, Praed St, London, United Kingdom.
‡Senior author.
ae105@cam.ac.uk

Contents

1 Master code **2**
- 1.1 Master 1: Load and prepare dataset . . . 2
- 1.2 Master 2: Test interactions with logistic regression models . . . 3
- 1.3 Master 3: Machine learning hyper-parameter optimisation . . . 3
- 1.4 Master 4: Training of optimised models on all imputations . . . 6
- 1.5 Master 5: Exploration of predictions made by deep learning and APACHE models . 9

2 Functions and subscripts **10**
- 2.1 Load and prepare dataset . . . 10
- 2.2 Cleaning of data . . . 11
  - 2.2.1 transformVentilationData . . . 11
  - 2.2.2 removeOutliers . . . 11
- 2.3 Imputation of missing data . . . 13
  - 2.3.1 parlMICE . . . 13
  - 2.3.2 imputeCustom . . . 14
  - 2.3.3 completeCustom . . . 15
- 2.4 Logistic regression interactions formulae . . . 15
- 2.5 Machine learning hyperparameter optimisation . . . 16
  - 2.5.1 Formulae . . . 16
  - 2.5.2 saveRDSFiles . . . 24
  - 2.5.3 tuneMachineLearningModels . . . 24
  - 2.5.4 tuneDeepLearningModel . . . 26
- 2.6 Interpret results of hyperparameter optimisation . . . 30
  - 2.6.1 buildPredResDF . . . 30
  - 2.6.2 generateCustomSummary . . . 30
  - 2.6.3 generateModelDF . . . 32
  - 2.6.4 getBestTune . . . 33
  - 2.6.5 getBestTunePreds . . . 33
  - 2.6.6 getTuningParams . . . 34
- 2.7 Building final models across all imputations . . . 35
  - 2.7.1 imputationVariationModels . . . 35
  - 2.7.2 getImputationResults . . . 36
  - 2.7.3 generateFoldedImputationSummaries . . . 37
- 2.8 Explore predictions . . . 38
  - 2.8.1 addVariableToExplorePredsDF . . . 38
- 2.9 Graph plotting functions . . . 39
  - 2.9.1 generateRootDir . . . 39
  - 2.9.2 plotMiscGraph . . . 39
  - 2.9.3 Plot patient outcome waterfall . . . 39
  - 2.9.4 plotDescriptiveGraphs & plotBeanplots . . . 40
  - 2.9.5 manualDensityPlots . . . 44
  - 2.9.6 plotTuningGraphs . . . 46
  - 2.9.7 plotMLOutputGraph . . . 49
  - 2.9.8 plotMLOutputGraph_vImp . . . 52
  - 2.9.9 plotImputationResults . . . 55
  - 2.9.10 plotPredictionsBeanplotPanel . . . 57

The code is organised into five master code scripts which call subscripts and functions which are defined as named scripts.

1 Master code

1.1 Master 1: Load and prepare dataset

```
#### Master Code : Loads dataset, cleans outliers and imputes missing data  ####

set.seed(42)

# load functions
source("functions/graph_plotting/plotDescriptiveGraphs.R") # functions for plotting descriptive graphs
source("functions/graph_plotting/plotMiscGraph.R") # function to plot generic graph to png file
source("functions/outliers/removeOutliers.R") # functions for removal of outliers
source("functions/outliers/transformVentilationData.R") # function for removal of outliers
source("functions/imputation/imputeCustom.R") # function for imputation of missing data
source("functions/imputation/completeCustom.R") # function for imputation of missing data
source("functions/graph_plotting/manualDensityPlots.R") # function for imputation of missing data

#### 1. Prepare dataSet ####
source("functions/loadData.R") # loads dataset into a formatted dataframe

# cut patients who have no classifier
dataSet.all <- filter(dataSet, !is.na(alive))

# factorise the classifiers
dataSet.all$alive_dead_icu = as.factor(dataSet.all$alive_dead_icu)
levels(dataSet.all$alive_dead_icu) = c("Alive","Dead","E")
levels(dataSet.all$alive) <- c("dead","alive")

# plot raw graphs
plotDescriptiveGraphs(dataSet.all, directory = "Graphs-Raw", 4000, 2000)

#### 2. Remove outliers ####
# apply transformation to ventilation data
tempDataSet.all <- transformVentilationData(dataSet.all)

# remove outliers, treating each variable, max/min, for each day as a sepearte distribution
cleanData.all <- removeOutliers(tempDataSet.all, "singleDaySeparate")

# plot cleaned graphs
plotDescriptiveGraphs(cleanData.all,
                      directory = "Graphs-Cleaned", 4000, 2000)
plotDescriptiveGraphs(cleanData.all, directory = "Graphs-Cleaned-noTitles", 4000, 2500, titled = FALSE, legend = FALSE, ptSize = 20)


#### 3. Impute missing values ####
imputedData.all <- impute.custom(cleanData.all) ########### TODO: need to test imputation
saveRDS(imputedData.all, "imputedData.all.RDS")

# plot graphs comparing imputations and original distribution
manualDensityPlots(mids = imputedData.all, cleanData = cleanData.all)
manualDensityPlots(mids = imputedData.all, cleanData = cleanData.all, titles = FALSE, minititles = FALSE, plotDir = "densityPLots_manual_vFig")


manualDensityPlots(mids = imputedData.all, cleanData = cleanData.all, titles = FALSE, minititles = FALSE, plotDir = "densityPLots_manual_vFig_minMax", minMax = "min", width = 3200, height = 800)
manualDensityPlots(mids = imputedData.all, cleanData = cleanData.all, titles = FALSE, minititles = FALSE, plotDir = "densityPLots_manual_vFig_minMax", minMax = "max", width = 3200, height = 800)

# save an imputed dataset as an RDS file
fullData.imput1 <- completeCustom(imputedData.all, 1)
```

```
saveRDS(fullData.imput1, "fullData.imput1.RDS")
```

1.2 Master 2: Test interactions with logistic regression models

```
#### Master Code: Discovers and tests simple interactions for logistic regression between age & MAP, lactate & MAP and lactate and noradrenaline ####

library(caret)
library(doMC)

source("functions/imputation/completeCustom.R") # function for imputation of missing data
source("functions/glmInteractions/interactionsFormulae.R") # formula for interactions

registerDoMC(detectCores() - 1)

### Set seeds
set.seed(42)
seed.list <- list()
for (i in 1:20) {
  seed.list[[i]] <- sample.int(n = 10000, size = 200)
}
seed.list[[21]] <- sample.int(n = 10000, size = 1)
seed.list

train.control <- trainControl(method = "LGOCV",
                              number = 20,
                              summaryFunction = twoClassSummary,
                              classProbs = TRUE,
                              verboseIter = TRUE,
                              seeds = seed.list,
                              savePredictions = "all",
                              returnResamp = "all",
                              p = 0.8)

dataset <- complete.custom(imputedData.all, 1)

classifier.allInt <- train(formula.allInt.day1,
                           dataset,
                           method = "glm",
                           metric = "ROC",
                           trControl = train.control,
                           preProcess = c("YeoJohnson", "center", "scale"))

summary(classifier.allInt)

classifier.selectInt <- train(formula.selectInt.day1,
                           dataset,
                           method = "glm",
                           metric = "ROC",
                           trControl = train.control,
                           preProcess = c("YeoJohnson", "center", "scale"))

summary(classifier.selectInt)

rm(dataset, classifier.allInt, classifier.selectInt, train.control)
```

1.3 Master 3: Machine learning hyper-parameter optimisation

```
#### Master Code: Tuning of machine learning models by 20 fold cross-vlaidation to discover optimal values for tuning parameters ####

#### 1. Set up environment ####

# load the functions
source("functions/MLtuning/tuneDeepLearningModel.R") # functions to build deep learning models
source("functions/MLtuning/tuneMachineLearningModels.R") # functions to train logistic regressions and machine learning models
source("functions/MLtuning_output/generateModelDF.R") # defines a function to determine which models have been completed
```

```
source("functions/MLtuning_output/buildPredResDF.R") # function to compile results or predictions into one dataframe
source("functions/MLtuning_output/getBestTunePreds.R") # function to compile validation set predictions for the optimal tuning parameters
source("functions/MLtuning_output/generateCustomSummary.R") # function to summarise validation predicions into ROC, sensitivity, specificity, etc.
source("functions/graph_plotting/plotTuningGraphs.R")
source("functions/graph_plotting/plotMLOutputGraph.R")

# set directory to save the predictions and results files
path.save <- "~/RDSfiles"

# set seeds for tuning caret models
set.seed(42)
seed.list <- list()
for (i in 1:20) {
  seed.list[[i]] <- sample.int(n = 10000, size = 200)
}
seed.list[[21]] <- sample.int(n = 10000, size = 1)
seed.list

# set the number of parallel cores
no.parallel.cores <- floor(2 * detectCores() / 3)
registerDoMC(cores = no.parallel.cores)

# define the train control for all models
train.control <- trainControl(method = "LGOCV",
                              number = 20,
                              summaryFunction = twoClassSummary,
                              classProbs = TRUE,
                              verboseIter = FALSE,
                              seeds = seed.list,
                              savePredictions = "all",
                              returnResamp = "all",
                              p = 0.8)

#### 2. First tuning run ####

# define the tuning grids for the first run
tune.grid.avNNet <- expand.grid(size = c(2,3,5,10,20),
                                decay = c(0.5,1,3,4,5,10),
                                bag = c(TRUE,FALSE))
tune.grid.svmRdialWeights <- expand.grid(C = c(1,3,5,10,20),
                                         Weight = c(0.1,0.5,1,2,3,5,10),
                                         sigma = c(0.0005,0.001,0.005,0.01,0.05))
tune.grid.adaboost <- expand.grid(method = "Adaboost.M1", nIter = c(10,30,100,300,1000))
tune.grid.DeepNN <- expand.grid(complexity.multiplier = c(0.3,0.5,0.8,1,1.2),
                                activation.layer_1 = c('tanh', 'relu'),
                                activation.layer_2 = c('tanh', 'relu'),
                                activation.layer_3 = c('sigmoid', 'tanh', 'relu'),
                                activation.layer_4 = 'tanh',
                                activation.layer_5 = 'relu',
                                activation.layer_6 = 'relu',
                                rate.dropout_1 = c(0.3,0.4),
                                rate.dropout_2 = c(0.2,0.3),
                                rate.dropout_3 = 0.2,
                                rate.dropout_4 = 0.2,
                                rate.dropout_5 = 0.2,
                                rate.dropout_6 = 0.2,
                                Epochs = c(30,50))

# run the model building function
iter <- 1 # iteration 1 for caret models
deep.iter <- 1 # iteration 1 for deep learning models
ML.methods <- c("adaboost", "APACHE", "avNNet", "DeepNN", "glm", "parRF", "svmRadialWeights") # define the methods to use
day.cumul <- data.frame(day = c(1,2,2,3,3,4,4,5,5),
                        cumul = c(F,F,T,F,T,F,T,F,T)) # define the days on which to build models

tuneMachineLearningModels(Iter = iter,
                          DeepIter = deep.iter,
                          MLmethods = ML.methods,
```

```
                          seed.list = seed.list,
                          path.D = path.save,
                          dayList = day.cumul,
                          fullData.imput.in = fullData.imput1)


#### 3. Determine tuning outcomes ####

# determine which models have been run
model.df.all <- generateModelDF()

# combine the results into one dataframe for each machine learning method
# build dataframes of the results for each machine learning method
for (MLmethod in c("adaboost", "APACHE", "avNNet", "DeepNN", "glm", "parRF", "svmRadialWeights")) {
  assign(paste("ML.results.df.", MLmethod, sep = ""),
         buildPredResDF(MLmethod = MLmethod,
                        pred.res = "results",
                        model.df = model.df.all,
                        path = path.save))
}

# d. Plot graphs to inspect tuning results and determine any further tuning required
plotTuningGraphs()

#### 4. Second tuning run ####

# define the tuning grids for the second tuning run
tune.grid.adaboost <- expand.grid(method = "Adaboost.M1", nIter = c(3000,5000))
tune.grid.DeepNN <- expand.grid(complexity.multiplier = c(seq(from = 3/16, to = 20/16, by = 1/16)),
                                activation.layer_1 = 'tanh',
                                activation.layer_2 = 'tanh',
                                activation.layer_3 = 'sigmoid',
                                activation.layer_4 = 'tanh',
                                activation.layer_5 = 'relu',
                                activation.layer_6 = 'relu',
                                rate.dropout_1 = c(0.4,0.5,0.6),
                                rate.dropout_2 = c(0.3,0.4,0.5),
                                rate.dropout_3 = c(0.2,0.3),
                                rate.dropout_4 = 0.2,
                                rate.dropout_5 = 0.2,
                                rate.dropout_6 = 0.2,
                                Epochs = 30)
tune.grid.svmRadialWeights <- expand.grid(C = c(0.1,0.3,0.5,0.8),
                                          Weight = c(2,3,4,5,7.5,10),
                                          sigma = c(0.001,0.025,0.005,0.01))


# run the model building function
iter <- 2 # iteration 2 for caret models
deep.iter <- 1 + nrow(tune.grid.DeepNN) / (2 * no.parallel.cores) # calculate iteration for deep leanring models
ML.methods <- c("adaboost", "DeepNN", "svmRadialWeights")

tuneMachineLearningModels(Iter = iter,
                          DeepIter = deep.iter,
                          MLmethods = ML.methods,
                          seed.list = seed.list,
                          path.D = path.save,
                          dayList = day.cumul,
                          fullData.imput.in = fullData.imput1)

#### 5. Plot outputs ####

model.df.all <- generateModelDF()


# build dataframes of the results for each machine learning method
for (MLmethod in c("adaboost", "APACHE", "avNNet", "DeepNN", "glm", "parRF", "svmRadialWeights")) {
  assign(paste("ML.results.df.", MLmethod, sep = ""),
```

```
          buildPredResDF(MLmethod = MLmethod,
                         pred.res = "results",
                         model.df = model.df.all,
                         path = path.save))
}

# plot graphs to inspect tuning results and determine any further tuning required
plotTuningGraphs()

# Calculate summary statistics for all 20 folds for each day for each machine learning method (time consuming - may choose to operate in parallel)
for (MLmethod in c("adaboost", "APACHE", "avNNet", "DeepNN", "glm", "parRF", "svmRadialWeights")) {
  assign(paste("ML.resampleResults.df.", MLmethod, sep = ""),
         getBestTunePreds(MLmethod, path = path.save) %>% generateCustomSummary())
}

ML.resampleResults.df.ALL <- rbind(ML.resampleResults.df.adaboost,
                                   ML.resampleResults.df.avNNet,
                                   ML.resampleResults.df.parRF,
                                   ML.resampleResults.df.svmRadialWeights,
                                   ML.resampleResults.df.glm,
                                   ML.resampleResults.df.DeepNN,
                                   ML.resampleResults.df.APACHE)

# g. plot graphs to show the interim results before imputaiton variability has been assessed
plotMLOutputGraph(plotMetric = "AUROC",
                  plotName = "AUROC",
                  resampleResults = ML.resampleResults.df.ALL,
                  day.range = 5,
                  MLmethods = c("glm", "parRF", "adaboost", "avNNet", "svmRadialWeights", "DeepNN"),
                  file.name = "AUROC_allMLmethods",
                  lines.plot = FALSE)
plotMLOutputGraph(plotMetric = "AUROC",
                  plotName = "AUROC",
                  resampleResults = ML.resampleResults.df.ALL,
                  day.range = 5, MLmethods = c("glm"),
                  file.name = "AUROC_glm",
                  lines.plot = FALSE)
plotMLOutputGraph(plotMetric = "AUROC",
                  plotName = "AUROC",
                  resampleResults = ML.resampleResults.df.ALL,
                  day.range = 5, MLmethods = c("APACHE"),
                  file.name = "AUROC_APACHE",
                  lines.plot = FALSE)

# GLM vs. DeepNN
plotMLOutputGraph(plotMetric = "AUROC",
                  plotName = "AUROC",
                  resampleResults = ML.resampleResults.df.ALL,
                  day.range = 5,
                  MLmethods = c("glm", "DeepNN"),
                  file.name = "AUROC_glmDeepNN_lines",
                  lines.plot = TRUE)

# APACHE vs. DeepNN
plotMLOutputGraph(plotMetric = "AUROC",
                  plotName = "AUROC",
                  resampleResults = ML.resampleResults.df.ALL,
                  day.range = 5,
                  MLmethods = c("APACHE", "DeepNN"),
                  file.name = "AUROC_APACHEcompareDeepNN_lines",
                  lines.plot = TRUE)
```

1.4 Master 4: Training of optimised models on all imputations

```
#### Master Code: Tests tuned models on all imputations to determine imputation variability and folding variability to give final results ####

source("functions/MLtuning/tuneDeepLearningModel.R")
```

```
source("functions/finalModel/imputationVariationModels.R")
source("functions/finalModel/getImputationResults.R")
source("functions/finalModel/generateFoldedImputationSummaries.R")
source("functions/graph_plotting/plotImputationResults.R")
source("functions/graph_plotting/plotMLOutputGraph_vImp.R")


path.imputation ← "~/RDSFiles/imputationVariation"
imputedData.all ← readRDS("imputedData.all.RDS")

registerDoMC(detectCores() - 6) # set up parallel workers

#### 1. Build one 20-fold crossvalidated model per imputation per day ####
ML.methods ← c("adaboost", "APACHE", "avNNet", "DeepNN", "glm", "parRF", "svmRadialWeights")
day.cumul = data.frame(day = c(1,2,2,3,3,4,4,5,5), cumul = c(F,F,T,F,T,F,T,F,T))
imputationVariationModels(MLmethods = ML.methods,
                                                path.impVar = path.imputation,
                                                dayList = day.cumul) # uses the optimal tuning
                                                    parameters for each day to build models
                                                    for each of 9 imputations (for each method
                                                     & day)

#### 2. Aggregate, process and plot outputs of imputation variatiability assessment ####
# load the imputation results into a dataframe for each MLmethod
for (MLmethod in c("adaboost", "APACHE", "avNNet", "DeepNN", "glm", "parRF", "svmRadialWeights"))
    {
  assign(paste("imputation.results.", MLmethod, sep = ""),
         getImputationResults(MLmethod = MLmethod,
                              imputationPath = path.imputation,
                              output.type = "results",
                              invert = ifelse(MLmethod == "svmRadialWeights", TRUE, FALSE)))
}

# plot the variability for each machine learning method
for (MLmethod in c("adaboost", "APACHE", "avNNet", "DeepNN", "glm", "parRF", "svmRadialWeights"))
    {
  plotImputationResults(get(paste("imputation.results.", MLmethod, sep = "")), MLmethod)
}

# generate resampling summary statistics for each ML method (time consuming, not parallel, kept
    separate to limit memory use)
imputation.resampleResults.APACHE ← getImputationResults(MLmethod = "APACHE",
                                                         imputationPath = path.imputation,
                                                         output.type = "pred") %>%
                                    generateFoldedImputationSummaries()

imputation.resampleResults.glm  ← getImputationResults(MLmethod = "glm",
                                                       imputationPath = path.imputation,
                                                       output.type = "pred") %>%
                                  generateFoldedImputationSummaries()

imputation.resampleResults.DeepNN ← getImputationResults(MLmethod = "DeepNN",
                                                         imputationPath = path.imputation,
                                                         output.type = "pred") %>%
                                    generateFoldedImputationSummaries()

imputation.resampleResults.svmRadialWeights ← getImputationResults(MLmethod = "svmRadialWeights",
                                                                   imputationPath = path.
                                                                       imputation,
                                                                   output.type = "pred") %>%
                                              generateFoldedImputationSummaries(invert = TRUE)

imputation.resampleResults.avNNet ← getImputationResults(MLmethod = "avNNet",
                                                         imputationPath = path.imputation,
                                                         output.type = "pred") %>%
                                    generateFoldedImputationSummaries()

imputation.resampleResults.parRF  ← getImputationResults(MLmethod = "parRF",
                                                         imputationPath = path.imputation,
                                                         output.type = "pred") %>%
                                    generateFoldedImputationSummaries()

```

```
imputation.resampleResults.adaboost ← getImputationResults(MLmethod = "adaboost",
                                                           imputationPath = path.imputation,
                                                           output.type = "pred") %>%
                                      generateFoldedImputationSummaries()



# d. Aggregate to give combined dataframes for the overall results
imputation.results.ALL ← rbind(imputation.results.glm[,c(1:3,(length(imputation.results.glm)-5):
    length(imputation.results.glm))],
                               imputation.results.DeepNN[,c(1:3,(length(imputation.results.
                                   DeepNN)-5):length(imputation.results.DeepNN))],
                               imputation.results.APACHE[,c(1:3,(length(imputation.results.
                                   APACHE)-5):length(imputation.results.APACHE))],
                               imputation.results.avNNet[,c(1:3,(length(imputation.results.
                                   avNNet)-5):length(imputation.results.avNNet))],
                               imputation.results.svmRadialWeights[,c(1:3,(length(imputation.
                                   results.svmRadialWeights)-5):length(imputation.results.
                                   svmRadialWeights))],
                               imputation.results.parRF[,c(1:3,(length(imputation.results.parRF)
                                   -5):length(imputation.results.parRF))],
                               imputation.results.adaboost[,c(1:3,(length(imputation.results.
                                   adaboost)-5):length(imputation.results.adaboost))])

imputation.resampleResults.ALL ← rbind(imputation.resampleResults.glm,
                                       imputation.resampleResults.DeepNN,
                                       imputation.resampleResults.APACHE,
                                       imputation.resampleResults.svmRadialWeights,
                                       imputation.resampleResults.adaboost,
                                       imputation.resampleResults.parRF,
                                       imputation.resampleResults.avNNet)

### Pool the imputations and resamples to give one overall estimate of variance
imputation.results.ALL.pooled ← imputation.results.ALL %>%
                                 group_by(MLmethod, day, cumulative) %>%
                                 summarise(ROCmean = mean(ROC),
                                           ROCImpVar = sd(ROC)^2,
                                           ROCResampleVar = mean(ROCSD^2),
                                           ROCPooledVar = ROCImpVar + ROCResampleVar,
                                           ROCPooledSD = sqrt(ROCPooledVar),
                                           ROCul = ROCmean + 2*ROCPooledSD,
                                           ROCll = ROCmean - 2*ROCPooledSD)

plotMLOutputGraph_vImp(imputationResampleResultsALL = imputation.resampleResults.ALL,
                       MLmethods = c("glm", "parRF", "avNNet", "adaboost", "svmRadialWeights", "
                           DeepNN"),
                       plotMetric = "AUROC",
                       plotName = "AUROC_allML",
                       legendPosition = "topright",
                       title = FALSE,
                       errorBarScale = 0.4,
                       yLabel = "AUC",
                       height = 3500,
                       vLines = TRUE,
                       barColour = "black",
                       dotSize = 0.65,
                       lineThickness = 1.2)

plotMLOutputGraph_vImp(imputationResampleResultsALL = imputation.resampleResults.ALL,
                       MLmethods = c("glm", "parRF", "avNNet", "adaboost", "svmRadialWeights", "
                           DeepNN"),
                       plotMetric = "AUROC",
                       plotName = "AUROC_allML_APACHE",
                       legendPosition = "topright",
                       title = FALSE,
                       errorBarScale = 0.35,
                       addAPACHEday1 = TRUE,
                       yLabel = "AUC", height = 3500,
                       vLines = TRUE,
                       barColour = "black",
                       dotSize = 0.65,
                       lineThickness = 1.2)
```

```
131
132 plotMLOutputGraph_vImp(imputationResampleResultsALL = imputation.resampleResults.ALL,
133                        MLmethods = c("APACHE"),
134                        plotMetric = "AUROC",
135                        plotName = "APACHE",
136                        legendPosition = "topright",
137                        title = FALSE,
138                        errorBarScale = 0.1,
139                        yLabel = "AUC",
140                        height = 2000,
141                        legend = FALSE,
142                        width = 3000)
```

1.5 Master 5: Exploration of predictions made by deep learning and APACHE models

```
1  #### Master Code: Explores differences between correct and incorrect predictions for the models
       in age and length of admission ####
2
3  source("functions/finalModel/getImputationResults.R")
4  source("functions/imputation/completeCustom.R")
5  source("functions/predictionExploration/addVariableToExplorePredsDF.R")
6  source("functions/graph_plotting/plotPredictionsBeanplotPanel.R")
7  source("functions/graph_plotting/plotDescriptiveGraphs.R")
8
9  library(dplyr)
10
11 # set path of final model predictions
12 path.imputation <- "~/RDSFiles/imputationVariation"
13
14 # get all predictiions, generate summaries and aggregate into one large data frame
15 imputation.explorePreds.all <- data.frame()
16 for(MLmethod in c("adaboost", "APACHE", "avNNet", "DeepNN", "glm", "parRF", "svmRadialWeights"))
       {
17    assign(paste("imputation.allPreds.", MLmethod, sep = ""),
18           getImputationResults(MLmethod = MLmethod,
19                                imputationPath = path.imputation,
20                                output.type = "pred"))
21
22   temp.df <- transmute(get(paste("imputation.allPreds.", MLmethod, sep = "")),
23                        MLmethod = MLmethod,
24                        rowIndex = rowIndex,
25                        imputation = imputation,
26                        day = day,
27                        cumulative = cumulative,
28                        obs = obs,
29                        pred = pred,
30                        alive = alive,
31                        correct = ifelse(pred == obs, TRUE, FALSE))
32
33   imputation.explorePreds.all <- rbind(imputation.explorePreds.all, temp.df)
34 }
35 saveRDS(imputation.explorePreds.all, "imputation.explorePreds.all.RDS")
36
37 # retrieve the predictions for the deep learning models and look up the values of all
       corresponding variables
38 imputation.explorePreds.DeepNN <- filter(imputation.explorePreds.all,
39                                          MLmethod == "DeepNN")
40
41 predictionOutputs.df.DeepNN <- addAllVariablesToExplorePredsDF(imputation.explorePreds.DeepNN)
42
43 # plot the beanplots to compare distributions
44 plotPredictionsBeanplotPanel(variable = "age",
45                              inputDF = predictionOutputs.df.DeepNN,
46                              density = 2,
47                              MLmethod = "Deep Learning",
48                              title = FALSE, footer = FALSE)
49
50 plotPredictionsBeanplotPanel(variable = "icu_duration_days",
51                              inputDF = predictionOutputs.df.DeepNN,
```

```
                              density = 2,
                              MLmethod = "Deep Learning",
                              ylimits = c(-10,60),
                              title = FALSE,
                              footer = FALSE)

# generate tabular version
deepLearningTable ← predictionOutputs.df.DeepNN %>%
  group_by(day, cumulative, obs, correct) %>%
  summarise(meanAge = round(mean(age),2),
            sdAge = round(sd(age),2),
            meanLOS = round(mean(icu_duration_days),2),
            sdLOS = round(sd(icu_duration_days),2))

deepLearningTable


# Repeat for APACHE-II

# retrieve the predictions for the deep learning models and look up the values of all
    corresponding variables
imputation.explorePreds.APACHE ← filter(imputation.explorePreds.all,
                                        MLmethod == "APACHE")

predictionOutputs.df.APACHE ← addAllVariablesToExplorePredsDF(imputation.explorePreds.APACHE)

# plot the beanplots to compare distributions
plotPredictionsBeanplotPanel(variable = "age",
                              inputDF = predictionOutputs.df.APACHE,
                              density = 2,
                              MLmethod = "APACHE",
                              title = FALSE, footer = FALSE)

plotPredictionsBeanplotPanel(variable = "icu_duration_days",
                              inputDF = predictionOutputs.df.APACHE,
                              density = 2,
                              MLmethod = "APACHE",
                              ylimits = c(-10,60),
                              title = FALSE,
                              footer = FALSE)

plotPredictionsBeanplotPanel(variable = "",
                              inputDF = predictionOutputs.df.APACHE,
                              density = 2,
                              MLmethod = "APACHE",
                              ylimits = c(-10,60),
                              title = FALSE,
                              footer = FALSE)
```

2 Functions and subscripts

These scripts are mostly functions called by the master code or each other and the sections are titled according to the name of the function to be called externally from the script.

2.1 Load and prepare dataset

```
### Script to load data and set up dataSet

library(readr)
library(readxl)
library(dplyr)

rawData = read_csv("dataset/dataSet.csv")

# reconstruct data set
dataSet ← mutate(rawData,
                 alive = factor(alive_dead_icu, levels = c('D', 'A'), labels = c(0,1)),
                 sex = as.factor(sex),
```

```
                  day1Ventilated = as.factor(day1Ventilated),
                  day2Ventilated = as.factor(day2Ventilated),
                  day3Ventilated = as.factor(day3Ventilated),
                  day4Ventilated = as.factor(day4Ventilated),
                  day5Ventilated = as.factor(day5Ventilated),
                  day1AdrenalineTotal = as.numeric(day1AdrenalineTotal),
                  day2AdrenalineTotal = as.numeric(day2AdrenalineTotal),
                  day3AdrenalineTotal = as.numeric(day3AdrenalineTotal),
                  day4AdrenalineTotal = as.numeric(day4AdrenalineTotal),
                  day5AdrenalineTotal = as.numeric(day5AdrenalineTotal),
                  day1VasopressinTotal = as.numeric(day1VasopressinTotal),
                  day2VasopressinTotal = as.numeric(day2VasopressinTotal),
                  day3VasopressinTotal = as.numeric(day3VasopressinTotal),
                  day4VasopressinTotal = as.numeric(day4VasopressinTotal),
                  day5VasopressinTotal = as.numeric(day5VasopressinTotal),
                  day1minLactate = as.numeric(day1minLactate),
                  day2minLactate = as.numeric(day2minLactate),
                  day3minLactate = as.numeric(day3minLactate),
                  day4minLactate = as.numeric(day4minLactate),
                  day5minLactate = as.numeric(day5minLactate),
                  day1maxLactate = as.numeric(day1maxLactate),
                  day2maxLactate = as.numeric(day2maxLactate),
                  day3maxLactate = as.numeric(day3maxLactate),
                  day4maxLactate = as.numeric(day4maxLactate),
                  day5maxLactate = as.numeric(day5maxLactate))


### load list of variables
allVariablesDF ← read_excel("support_data/allVariableList.xlsx")
allVariablesDF$day ← as.integer(allVariablesDF$day)
```

2.2 Cleaning of data

2.2.1 transformVentilationData

```
# function to transform the ventialtion data by dividing values above 80 by 7.6

transformVentilationData ← function(dataSet) {
  tempDataSet ← dataSet
  for(i in 1:5) {
    for (j in c("min", "max")) {
      multiplier ← (6.6 * as.numeric(dataSet[[paste("day",i,j,"PaO2FiO2",sep="")]] > 80)) + 1
      tempDataSet[[paste("day",i,j,"PaO2FiO2",sep="")]] ← (dataSet[[paste("day",i,j,"PaO2FiO2",
          sep="")]] / multiplier)
      print("Transformed PaO2FiO2 data")
    }
  }
  return(tempDataSet)
}
```

2.2.2 removeOutliers

```
### function to remove outliers, calling a function to set the limites based on one of three
    strategies

generateLimits ← function(dataSet, method = "singleDaySeparate") {
  limitsDF ← data.frame(variable = allVariablesDF$variable, UL = rep(NA, nrow(allVariablesDF)),
      LL = rep(NA, nrow(allVariablesDF)))


  if (method == "singleDaySeparate") {

    ### set limits based on mena and sd of each variable on each day, separated by min and max
    for (variableNo in 1:nrow(allVariablesDF)) {
      if (allVariablesDF$class[variableNo] != "factor") {
        varVals ← unlist(select(dataSet, contains(allVariablesDF$variable[variableNo])))
        limitsDF[variableNo, "UL"] ← mean(varVals, na.rm = TRUE) + 5 * sd(varVals, na.rm = TRUE)
        limitsDF[variableNo, "LL"]  ← mean(varVals, na.rm = TRUE) - 5 * sd(varVals, na.rm = TRUE)
      }
    }
```

```
17
18
19    } else if (method == "singleDayCombined") {
20
21      ### set limits based on mean and sd of each variable on each day, regardless of min/max
22      for (variableNo in 1:nrow(allVariablesDF)) {
23        if (allVariablesDF$class[variableNo] != "factor") {
24          if (is.na(allVariablesDF$minMax[variableNo])) {
25            varVals <- unlist(select(dataSet, contains(allVariablesDF$variable[variableNo])))
26            limitsDF[variableNo, "UL"] <- mean(varVals, na.rm = TRUE) + 5 * sd(varVals, na.rm = TRUE
                  )
27            limitsDF[variableNo, "LL"]  <- mean(varVals, na.rm = TRUE) - 5 * sd(varVals, na.rm =
                  TRUE)
28          } else {
29            for(day in 1:5) {
30              varVals <- unlist(dataSet %>% select(contains(allVariablesDF$variableTitle[variableNo
                      ])) %>% select(contains(paste("day", day, sep = ""))))
31              limitsDF[variableNo, "UL"] <- mean(varVals, na.rm = TRUE) + 5 * sd(varVals, na.rm =
                      TRUE)
32              limitsDF[variableNo, "LL"]  <- mean(varVals, na.rm = TRUE) - 5 * sd(varVals, na.rm =
                      TRUE)
33            }
34          }
35        }
36      }
37
38    } else if (method == "multiDayCombined") {
39
40      ### set limits based on mean and sd of each variable, regardless of day, regardless of min/
            max
41      for (variableNo in 1:nrow(allVariablesDF)) {
42        if (allVariablesDF$class[variableNo] != "factor") {
43          if (is.na(allVariablesDF$minMax[variableNo])) {
44            varVals <- unlist(select(dataSet, contains(allVariablesDF$variableTitle[variableNo])))
45            limitsDF[variableNo, "UL"] <- mean(varVals, na.rm = TRUE) + 5 * sd(varVals, na.rm = TRUE
                  )
46            limitsDF[variableNo, "LL"]  <- mean(varVals, na.rm = TRUE) - 5 * sd(varVals, na.rm =
                  TRUE)
47          } else {
48            varVals <- unlist(dataSet %>% select(contains(allVariablesDF$variableTitle[variableNo]))
                       %>% select(contains("day1"), contains("day2"), contains("day3"), contains("day4")
                  , contains("day5")))
49            limitsDF[variableNo, "UL"] <- mean(varVals, na.rm = TRUE) + 5 * sd(varVals, na.rm = TRUE
                  )
50            limitsDF[variableNo, "LL"]  <- mean(varVals, na.rm = TRUE) - 5 * sd(varVals, na.rm =
                  TRUE)
51          }
52        }
53      }
54    }
55
56    ### set manual limits on particular variables
57    limitsDF[grepl("Potassium", limitsDF$variable), "UL"] <- 10 # potassium upper limit of 10
58    limitsDF[grepl("Lactate", limitsDF$variable), "UL"] <- 20 # lactate upper limit of 10
59    limitsDF[grepl("Lactate", limitsDF$variable), "LL"] <- 0 # no lower limit on lactate
60    limitsDF[grepl("Creatinine", limitsDF$variable), "LL"] <- 0 # no lower limit on creatinine
61    limitsDF[grepl("Adrenaline|Noradrenaline|Vasopressin|CRP|age|apache_score", limitsDF$variable),
            "UL"] <- 1000000000 # no limits on Adrenaline, Noradrenaline, Vasopressin, CRP and age
62    limitsDF[grepl("Adrenaline|Noradrenaline|Vasopressin|CRP|age|apache_score", limitsDF$variable),
            "LL"] <- 0 # no limits on Adrenaline, Noradrenaline, Vasopressin, CRP and age
63
64    return(limitsDF)
65  }
66
67  removeOutliers <- function(dataSet, method) {
68    limitsDF <- generateLimits(dataSet = dataSet, method = method)
69
70    returnDF <- dataSet
71
72    for (variable in allVariablesDF$variable[allVariablesDF$outlierRemoval == TRUE]) {
73      outliers <- dataSet[[variable]] > limitsDF[limitsDF$variable == variable, "UL"] | dataSet[[
            variable]] < limitsDF[limitsDF$variable == variable, "LL"]
```

```
    returnDF[[variable]] ← ifelse(outliers == TRUE,
                                  NA,
                                  dataSet[[variable]])
  }

  return(returnDF)
}
```

2.3 Imputation of missing data

2.3.1 parlMICE

From https://github.com/gerkovink/parlMICE, accessed 15th January 2018.

```
#'Parallel function for MICE
#'
#'This is a wrapper function for \code{\link{mice}}, using multiple cores to
#'execute \code{\link{mice}} in parallel. As a result, the imputation
#'procedure is sped up, which might be useful in general.
#'
#'This function is built upon package \code{\link{parallel}}, which is a base
#'package for R versions 2.14.0 and later. We have chosen to use parallel function
#'\code{parLapply} to allow the use of \code{parallelMICE} on Mac, Linux and Windows
#'systems. For the same reason, we use the Parallel Socket Cluster (PSOCK) type.
#'
#'On systems other than Windows, it is recommended to change the cluster type to
#'\code{FORK}, as it is better in handling the memory space. When memory issues
#'arise on a Windows system, we advise to store the multiply imputed datasets,
#'clean the memory by using \code{\link{rm}} and \code{\link{gc}} and make another
#'run using the same settings. For more tips about dealing with memory problems,
#'we refer to Max Gordon's document *How-to go parallel in R - basics + tips*.
#'
#'This wrapper function combines the output of \code{\link{parLapply}} with
#'function \code{\link{ibind}} in \code{\pkg{mice}}. A \code{mids} object is returned
#'and can be used for further analyses.
#'
#'Note that if a seed value is desired, the seed should be entered to this function
#'with argument \code{seed}. Seed values outside the wrapper function (in an
#'R-script or passed to \code{\link{mice}}) will not result to reproducible results.
#'We refer to the manual of \code{\pkg{parallel}} for an explanation on this matter.
#'
#'A vignette describing the use of 'parlMICE' can be found in the \code{\pkg{mice}}
#'package or through Github: https://github.com/gerkovink/parallelMICE/blob/master.
#'
#'@param data A data frame or matrix containing the incomplete data. Similar to
#'the first argument of \code{\link{mice}}.
#'@param n.core A scalar indicating the number of cores that should be used. Default
#'is the number of logical cores minus 1.
#'@param n.imp.core A scalar indicating the number of imputations per core. The
#'total number of imputations will be equal to n.core * n.imp.core.
#'@param seed A scalar to be used as the seed value. It is recommended to put the
#'seed value here and not outside this function, as otherwise the parallel processes
#'will be performed with separate, random seeds.
#'@param ... Named arguments that are passed down to function \code{\link{mice}} or
#'\code{\link{makeCluster}}.
#'
#'@return A mids object as defined by \code{\link{mids-class}}
#'
#'@author Rianne Schouten, Gerko Vink, 2016, with many thanks to Max Gordon.
#'@seealso \code{\pkg{parallel}}, \code{\link{parLapply}}, \code{\link{makeCluster}},
#'\code{\link{mice}}, \code{\link{mids-class}}, Vignette \emph{Wrapper function parlMICE}
#'@references
#'Gordon, M. (2015). How-to go parallel in R - basics + tips. Available through
#'[link](http://gforge.se/2015/02/how-to-go-parallel-in-r-basics-tips/)
#'
#'Van Buuren, S. (2012). \emph{Flexible imputation of missing data.}
#'Boca Raton, FL.: Chapman & Hall/CRC Press.
#'@examples
#'# 150 imputations in dataset nhanes, performed by 3 cores
#'result1 ← parlMICE(data = nhanes, n.core = 3, n.imp.core = 50)
#'# Making use of arguments in \code{mice}.
```

```
#'result2 ← parlMICE(data = nhanes, method = "norm.nob", m = 100)
#'with(result2, lm(bmi ~ hyp))
#'# On systems other than Windows, use type = "FORK"
#'result3 ← parlMICE(data = nhanes, type = "FORK", n.imp.core = 100)
#'
#'@export
parlMICE ← function(data, n.core = detectCores() - 1, n.imp.core = 2,
                    seed = NULL, m = NULL, ...){
  suppressMessages(require(parallel))
  cl ← makeCluster(n.core, ...)
  clusterExport(cl, varlist = "data", envir = environment())
  clusterEvalQ(cl, library(mice))
  if (!is.null(seed)) {
    clusterSetRNGStream(cl, seed)
  }
  if (!is.null(m)) {
    n.imp.core ← ceiling(m / n.core)
  }
  imps ← parLapply(cl = cl, X = 1:n.core, fun = function(i){
    mice(data, print = FALSE, m = n.imp.core, ...)
  })
  stopCluster(cl)
  imp ← imps[[1]]
  if (length(imps) > 1) {
    for (i in 2:length(imps)) {
      imp ← ibind(imp, imps[[i]])
    }
  }
  return(imp)
}
```

2.3.2 imputeCustom

```
# function to impute the missing data, both for the variables missing not at random and those
    missing at random

source("functions/imputation/parlMICE.R") # parallel wrapper for the mice function

imputeNorAdVas ← function(cleanData) {
  outputData ← cleanData

  norAdVas = c("AdrenalineTotal","NoradrenalineTotal","VasopressinTotal")
  for(variable in norAdVas) {
    for(day in 1:5) {
      varString ← paste("day",day,variable,sep="")
      zeroValues ← is.na(cleanData[[varString]]) & (day <= cleanData$icu_duration_days)
      outputData[zeroValues,varString] ← 0
    }
    print(paste("Imputed drugs",variable,"data to add 0's in the place of NAs where the patient
        is still on ICU", sep = " "))
  }

  return(outputData)
}

### Step 2 --> perform imputation
miceCustom ← function(partiallyImputedData) {
  return(parlMICE(partiallyImputedData,
                  m = 9,
                  maxit = 20,
                  meth = 'pmm',
                  seed = 42))
}

### Combine Steps 1 & 2 in a wrapper function
imputeCustom ← function(cleanedData, dayCutOff = 3) {
  cleanedData %>%
    imputeNorAdVas() %>%
    miceCustom()
```

```
}
```

2.3.3 completeCustom

```
# custom wrapper for the complete function to remove non-expected values

library(mice)
library(dplyr)

completeCustom ← function(mids, imput.number) {
  temp.imput ← mice::complete(mids, imput.number)

  for(colname in colnames(temp.imput)) {
    if (colname %in% filter(allVariablesDF, outlierRemoval == TRUE)$variable) {
      day ← allVariablesDF[allVariablesDF$variable == colname, ]$day
      naPos ← temp.imput$icu_duration_days < day
      temp.imput[naPos, colname] ← NA
    }
  }
  return(temp.imput)
}
```

2.4 Logistic regression interactions formulae

```
### Day 1
formula.allInt.day1 ← formula(alive ~
                                age
                              + sex
                              + day1minMAP + day1maxMAP
                              + day1minHR + day1maxHR
                              + day1maxLactate + day1minLactate
                              + day1minPotassium + day1maxPotassium
                              + day1minSodium + day1maxSodium
                              + day1minPH + day1maxPH
                              + day1Ventilated
                              + day1minCreatinine + day1maxCreatinine
                              + day1minPaO2FiO2 + day1maxPaO2FiO2
                              + day1minCRP + day1maxCRP
                              + day1NoradrenalineTotal + day1VasopressinTotal + day1
                                  AdrenalineTotal
                              + age:day1maxMAP
                              + age:day1minMAP
                              + day1minLactate:day1NoradrenalineTotal
                              + day1maxLactate:day1NoradrenalineTotal
                              + day1minLactate:day1maxMAP
                              + day1minLactate:day1minMAP
                              + day1maxLactate:day1maxMAP
                              + day1maxLactate:day1minMAP)

formula.selectInt.day1 ← formula(alive ~
                                age
                              + sex
                              + day1minMAP + day1maxMAP
                              + day1minHR + day1maxHR
                              + day1maxLactate + day1minLactate
                              + day1minPotassium + day1maxPotassium
                              + day1minSodium + day1maxSodium
                              + day1minPH + day1maxPH
                              + day1Ventilated
                              + day1minCreatinine + day1maxCreatinine
                              + day1minPaO2FiO2 + day1maxPaO2FiO2
                              + day1minCRP + day1maxCRP
                              + day1NoradrenalineTotal + day1VasopressinTotal + day1
                                  AdrenalineTotal
                              + age:day1maxMAP
                              + day1minLactate:day1NoradrenalineTotal
                              + day1minLactate:day1minMAP)
```

2.5 Machine learning hyperparameter optimisation

2.5.1 Formulae

```
# specify formulae for logistic regressions

#### 1. APACHE as single predictor
formula.APACHE ← formula(alive ~ apache_score)

#### 2. Simple formulae - each day seperate, each factor alone
formula.day1.simple ← formula(alive ~
                                age
                              + sex
                              + day1minMAP + day1maxMAP
                              + day1minHR + day1maxHR
                              + day1maxLactate + day1minLactate
                              + day1minPotassium + day1maxPotassium
                              + day1minSodium + day1maxSodium
                              + day1minPH + day1maxPH
                              + day1Ventilated
                              + day1minCreatinine + day1maxCreatinine
                              + day1minPaO2FiO2 + day1maxPaO2FiO2
                              + day1minCRP + day1maxCRP
                              + day1AdrenalineTotal + day1NoradrenalineTotal + day1
                                  VasopressinTotal)

formula.day2.simple ← formula(alive ~
                                age
                              + sex
                              + day2minMAP + day2maxMAP
                              + day2minHR + day2maxHR
                              + day2maxLactate + day2minLactate
                              + day2minPotassium + day2maxPotassium
                              + day2minSodium + day2maxSodium
                              + day2minPH + day2maxPH
                              + day2Ventilated
                              + day2minCreatinine + day2maxCreatinine
                              + day2minPaO2FiO2 + day2maxPaO2FiO2
                              + day2minCRP + day2maxCRP
                              + day2AdrenalineTotal + day2NoradrenalineTotal + day2
                                  VasopressinTotal)

formula.day3.simple ← formula(alive ~
                                age
                              + sex
                              + day3minMAP + day3maxMAP
                              + day3minHR + day3maxHR
                              + day3maxLactate + day3minLactate
                              + day3minPotassium + day3maxPotassium
                              + day3minSodium + day3maxSodium
                              + day3minPH + day3maxPH
                              + day3Ventilated
                              + day3minCreatinine + day3maxCreatinine
                              + day3minPaO2FiO2 + day3maxPaO2FiO2
                              + day3minCRP + day3maxCRP
                              + day3AdrenalineTotal + day3NoradrenalineTotal + day3
                                  VasopressinTotal)

formula.day4.simple ← formula(alive ~
                                age
                              + sex
                              + day4minMAP + day4maxMAP
                              + day4minHR + day4maxHR
                              + day4maxLactate + day4minLactate
                              + day4minPotassium + day4maxPotassium
                              + day4minSodium + day4maxSodium
                              + day4minPH + day4maxPH
                              + day4Ventilated
                              + day4minCreatinine + day4maxCreatinine
                              + day4minPaO2FiO2 + day4maxPaO2FiO2
                              + day4minCRP + day4maxCRP
```

```
                                + day4AdrenalineTotal + day4NoradrenalineTotal + day4
                                    VasopressinTotal)

formula.day5.simple ← formula(alive ~
                                  age
                                + sex
                                + day5minMAP + day5maxMAP
                                + day5minHR + day5maxHR
                                + day5maxLactate + day5minLactate
                                + day5minPotassium + day5maxPotassium
                                + day5minSodium + day5maxSodium
                                + day5minPH + day5maxPH
                                + day5Ventilated
                                + day5minCreatinine + day5maxCreatinine
                                + day5minPaO2FiO2 + day5maxPaO2FiO2
                                + day5minCRP + day5maxCRP
                                + day5AdrenalineTotal + day5NoradrenalineTotal + day5
                                    VasopressinTotal)


#### 3. Custom formulae to add interactions
# add together to a complete formula, days 1, 2 & 3
formula.day1.custom ← formula(alive ~
                                  age
                                + sex
                                + day1minMAP + day1maxMAP
                                + day1minHR + day1maxHR
                                + day1maxLactate + day1minLactate
                                + day1minPotassium + day1maxPotassium
                                + day1minSodium + day1maxSodium
                                + day1minPH + day1maxPH
                                + day1Ventilated
                                + day1minCreatinine + day1maxCreatinine
                                + day1minPaO2FiO2 + day1maxPaO2FiO2
                                + day1minCRP + day1maxCRP
                                + day1NoradrenalineTotal + day1VasopressinTotal + day1
                                    AdrenalineTotal
                                + day1minLactate:day1NoradrenalineTotal
                                + day1minLactate:day1minMAP
                                + age:day1maxMAP)

formula.day2.custom ← formula(alive ~
                                  age
                                + sex
                                + day2minMAP + day2maxMAP
                                + day2minHR + day2maxHR
                                + day2maxLactate + day2minLactate
                                + day2minPotassium + day2maxPotassium
                                + day2minSodium + day2maxSodium
                                + day2minPH + day2maxPH
                                + day2Ventilated
                                + day2minCreatinine + day2maxCreatinine
                                + day2minPaO2FiO2 + day2maxPaO2FiO2
                                + day2minCRP + day2maxCRP
                                + day2NoradrenalineTotal + day2VasopressinTotal + day2
                                    AdrenalineTotal
                                + day2minLactate:day2NoradrenalineTotal
                                + day2minLactate:day2minMAP
                                + age:day2maxMAP)

formula.day3.custom ← formula(alive ~
                                  age
                                + sex
                                + day3minMAP + day3maxMAP
                                + day3minHR + day3maxHR
                                + day3maxLactate + day3minLactate
                                + day3minPotassium + day3maxPotassium
                                + day3minSodium + day3maxSodium
                                + day3minPH + day3maxPH
                                + day3Ventilated
                                + day3minCreatinine + day3maxCreatinine
                                + day3minPaO2FiO2 + day3maxPaO2FiO2
```

```
                                + day3minCRP + day3maxCRP
                                + day3NoradrenalineTotal + day3VasopressinTotal + day3
                                    AdrenalineTotal
                                + day3minLactate:day3NoradrenalineTotal
                                + day3minLactate:day3minMAP
                                + age:day3maxMAP)

formula.day4.custom ← formula(alive ~
                                  age
                                + sex
                                + day4minMAP + day4maxMAP
                                + day4minHR + day4maxHR
                                + day4maxLactate + day4minLactate
                                + day4minPotassium + day4maxPotassium
                                + day4minSodium + day4maxSodium
                                + day4minPH + day4maxPH
                                + day4Ventilated
                                + day4minCreatinine + day4maxCreatinine
                                + day4minPaO2FiO2 + day4maxPaO2FiO2
                                + day4minCRP + day4maxCRP
                                + day4NoradrenalineTotal + day4VasopressinTotal + day4
                                    AdrenalineTotal
                                + day4minLactate:day4NoradrenalineTotal
                                + day4minLactate:day4minMAP
                                + age:day4maxMAP)

formula.day5.custom ← formula(alive ~
                                  age
                                + sex
                                + day5minMAP + day5maxMAP
                                + day5minHR + day5maxHR
                                + day5maxLactate + day5minLactate
                                + day5minPotassium + day5maxPotassium
                                + day5minSodium + day5maxSodium
                                + day5minPH + day5maxPH
                                + day5Ventilated
                                + day5minCreatinine + day5maxCreatinine
                                + day5minPaO2FiO2 + day5maxPaO2FiO2
                                + day5minCRP + day5maxCRP
                                + day5NoradrenalineTotal + day5VasopressinTotal + day5
                                    AdrenalineTotal
                                + day5minLactate:day5NoradrenalineTotal
                                + day5minLactate:day5minMAP
                                + age:day5maxMAP)

#### 4. Cumulative formulae

formula.day12.simple← formula(alive ~
                                  age
                                + sex
                                + day1minMAP + day1maxMAP
                                + day1minHR + day1maxHR
                                + day1maxLactate + day1minLactate
                                + day1minPotassium + day1maxPotassium
                                + day1minSodium + day1maxSodium
                                + day1minPH + day1maxPH
                                + day1Ventilated
                                + day1minCreatinine + day1maxCreatinine
                                + day1minPaO2FiO2 + day1maxPaO2FiO2
                                + day1minCRP + day1maxCRP
                                + day1AdrenalineTotal + day1NoradrenalineTotal + day1
                                    VasopressinTotal
                                + day2minMAP + day2maxMAP
                                + day2minHR + day2maxHR
                                + day2maxLactate + day2minLactate
                                + day2minPotassium + day2maxPotassium
                                + day2minSodium + day2maxSodium
                                + day2minPH + day2maxPH
                                + day2Ventilated
                                + day2minCreatinine + day2maxCreatinine
                                + day2minPaO2FiO2 + day2maxPaO2FiO2
                                + day2minCRP + day2maxCRP
```

```
201                                 + day2AdrenalineTotal + day2NoradrenalineTotal + day2
                                        VasopressinTotal)
202
203
204 formula.day123.simple <- formula(alive ~
205                                     age
206                                   + sex
207                                   + day1minMAP + day1maxMAP
208                                   + day1minHR + day1maxHR
209                                   + day1maxLactate + day1minLactate
210                                   + day1minPotassium + day1maxPotassium
211                                   + day1minSodium + day1maxSodium
212                                   + day1minPH + day1maxPH
213                                   + day1Ventilated
214                                   + day1minCreatinine + day1maxCreatinine
215                                   + day1minPaO2FiO2 + day1maxPaO2FiO2
216                                   + day1minCRP + day1maxCRP
217                                   + day1AdrenalineTotal + day1NoradrenalineTotal + day1
                                          VasopressinTotal
218                                   + day2minMAP + day2maxMAP
219                                   + day2minHR + day2maxHR
220                                   + day2maxLactate + day2minLactate
221                                   + day2minPotassium + day2maxPotassium
222                                   + day2minSodium + day2maxSodium
223                                   + day2minPH + day2maxPH
224                                   + day2Ventilated
225                                   + day2minCreatinine + day2maxCreatinine
226                                   + day2minPaO2FiO2 + day2maxPaO2FiO2
227                                   + day2minCRP + day2maxCRP
228                                   + day2AdrenalineTotal + day2NoradrenalineTotal + day2
                                          VasopressinTotal
229                                   + day3minMAP + day3maxMAP
230                                   + day3minHR + day3maxHR
231                                   + day3maxLactate + day3minLactate
232                                   + day3minPotassium + day3maxPotassium
233                                   + day3minSodium + day3maxSodium
234                                   + day3minPH + day3maxPH
235                                   + day3Ventilated
236                                   + day3minCreatinine + day3maxCreatinine
237                                   + day3minPaO2FiO2 + day3maxPaO2FiO2
238                                   + day3minCRP + day3maxCRP
239                                   + day3AdrenalineTotal + day3NoradrenalineTotal + day3
                                          VasopressinTotal)
240
241 formula.day1234.simple <- formula(alive ~
242                                     age
243                                   + sex
244                                   + day1minMAP + day1maxMAP
245                                   + day1minHR + day1maxHR
246                                   + day1maxLactate + day1minLactate
247                                   + day1minPotassium + day1maxPotassium
248                                   + day1minSodium + day1maxSodium
249                                   + day1minPH + day1maxPH
250                                   + day1Ventilated
251                                   + day1minCreatinine + day1maxCreatinine
252                                   + day1minPaO2FiO2 + day1maxPaO2FiO2
253                                   + day1minCRP + day1maxCRP
254                                   + day1AdrenalineTotal + day1NoradrenalineTotal + day1
                                          VasopressinTotal
255                                   + day2minMAP + day2maxMAP
256                                   + day2minHR + day2maxHR
257                                   + day2maxLactate + day2minLactate
258                                   + day2minPotassium + day2maxPotassium
259                                   + day2minSodium + day2maxSodium
260                                   + day2minPH + day2maxPH
261                                   + day2Ventilated
262                                   + day2minCreatinine + day2maxCreatinine
263                                   + day2minPaO2FiO2 + day2maxPaO2FiO2
264                                   + day2minCRP + day2maxCRP
265                                   + day2AdrenalineTotal + day2NoradrenalineTotal + day2
                                          VasopressinTotal
266                                   + day3minMAP + day3maxMAP
```

```
                                  + day3minHR + day3maxHR
                                  + day3maxLactate + day3minLactate
                                  + day3minPotassium + day3maxPotassium
                                  + day3minSodium + day3maxSodium
                                  + day3minPH + day3maxPH
                                  + day3Ventilated
                                  + day3minCreatinine + day3maxCreatinine
                                  + day3minPaO2FiO2 + day3maxPaO2FiO2
                                  + day3minCRP + day3maxCRP
                                  + day3AdrenalineTotal + day3NoradrenalineTotal + day3
                                      VasopressinTotal
                                  + day4minMAP + day4maxMAP
                                  + day4minHR + day4maxHR
                                  + day4maxLactate + day4minLactate
                                  + day4minPotassium + day4maxPotassium
                                  + day4minSodium + day4maxSodium
                                  + day4minPH + day4maxPH
                                  + day4Ventilated
                                  + day4minCreatinine + day4maxCreatinine
                                  + day4minPaO2FiO2 + day4maxPaO2FiO2
                                  + day4minCRP + day4maxCRP
                                  + day4NoradrenalineTotal + day4VasopressinTotal + day4
                                      AdrenalineTotal)

formula.day12345.simple<- formula(alive ~
                                    age
                                  + sex
                                  + day1minMAP + day1maxMAP
                                  + day1minHR + day1maxHR
                                  + day1maxLactate + day1minLactate
                                  + day1minPotassium + day1maxPotassium
                                  + day1minSodium + day1maxSodium
                                  + day1minPH + day1maxPH
                                  + day1Ventilated
                                  + day1minCreatinine + day1maxCreatinine
                                  + day1minPaO2FiO2 + day1maxPaO2FiO2
                                  + day1minCRP + day1maxCRP
                                  + day1AdrenalineTotal + day1NoradrenalineTotal + day1
                                      VasopressinTotal
                                  + day2minMAP + day2maxMAP
                                  + day2minHR + day2maxHR
                                  + day2maxLactate + day2minLactate
                                  + day2minPotassium + day2maxPotassium
                                  + day2minSodium + day2maxSodium
                                  + day2minPH + day2maxPH
                                  + day2Ventilated
                                  + day2minCreatinine + day2maxCreatinine
                                  + day2minPaO2FiO2 + day2maxPaO2FiO2
                                  + day2minCRP + day2maxCRP
                                  + day2AdrenalineTotal + day2NoradrenalineTotal + day2
                                      VasopressinTotal
                                  + day3minMAP + day3maxMAP
                                  + day3minHR + day3maxHR
                                  + day3maxLactate + day3minLactate
                                  + day3minPotassium + day3maxPotassium
                                  + day3minSodium + day3maxSodium
                                  + day3minPH + day3maxPH
                                  + day3Ventilated
                                  + day3minCreatinine + day3maxCreatinine
                                  + day3minPaO2FiO2 + day3maxPaO2FiO2
                                  + day3minCRP + day3maxCRP
                                  + day3AdrenalineTotal + day3NoradrenalineTotal + day3
                                      VasopressinTotal
                                  + day4minMAP + day4maxMAP
                                  + day4minHR + day4maxHR
                                  + day4maxLactate + day4minLactate
                                  + day4minPotassium + day4maxPotassium
                                  + day4minSodium + day4maxSodium
                                  + day4minPH + day4maxPH
                                  + day4Ventilated
                                  + day4minCreatinine + day4maxCreatinine
                                  + day4minPaO2FiO2 + day4maxPaO2FiO2
```

```
                                  + day4minCRP + day4maxCRP
                                  + day4NoradrenalineTotal + day4VasopressinTotal + day4
                                      AdrenalineTotal
                                  + day5minMAP + day5maxMAP
                                  + day5minHR + day5maxHR
                                  + day5maxLactate + day5minLactate
                                  + day5minPotassium + day5maxPotassium
                                  + day5minSodium + day5maxSodium
                                  + day5minPH + day5maxPH
                                  + day5Ventilated
                                  + day5minCreatinine + day5maxCreatinine
                                  + day5minPaO2FiO2 + day5maxPaO2FiO2
                                  + day5minCRP + day5maxCRP
                                  + day5NoradrenalineTotal + day5VasopressinTotal + day5
                                      AdrenalineTotal)

formula.day12.custom <- formula(alive ~
                                  age
                                + sex
                                + day1minMAP + day1maxMAP
                                + day1minHR + day1maxHR
                                + day1maxLactate + day1minLactate
                                + day1minPotassium + day1maxPotassium
                                + day1minSodium + day1maxSodium
                                + day1minPH + day1maxPH
                                + day1Ventilated
                                + day1minCreatinine + day1maxCreatinine
                                + day1minPaO2FiO2 + day1maxPaO2FiO2
                                + day1minCRP + day1maxCRP
                                + day1AdrenalineTotal + day1NoradrenalineTotal + day1
                                    VasopressinTotal
                                + day1minLactate:day1NoradrenalineTotal
                                + day1minLactate:day1minMAP
                                + age:day1maxMAP
                                + day2minMAP + day2maxMAP
                                + day2minHR + day2maxHR
                                + day2maxLactate + day2minLactate
                                + day2minPotassium + day2maxPotassium
                                + day2minSodium + day2maxSodium
                                + day2minPH + day2maxPH
                                + day2Ventilated
                                + day2minCreatinine + day2maxCreatinine
                                + day2minPaO2FiO2 + day2maxPaO2FiO2
                                + day2minCRP + day2maxCRP
                                + day2AdrenalineTotal + day2NoradrenalineTotal + day2
                                    VasopressinTotal
                                + day2minLactate:day2NoradrenalineTotal
                                + day2minLactate:day2minMAP
                                + age:day2maxMAP)

formula.day123.custom <- formula(alive ~
                                   age
                                 + sex
                                 + day1minMAP + day1maxMAP
                                 + day1minHR + day1maxHR
                                 + day1maxLactate + day1minLactate
                                 + day1minPotassium + day1maxPotassium
                                 + day1minSodium + day1maxSodium
                                 + day1minPH + day1maxPH
                                 + day1Ventilated
                                 + day1minCreatinine + day1maxCreatinine
                                 + day1minPaO2FiO2 + day1maxPaO2FiO2
                                 + day1minCRP + day1maxCRP
                                 + day1AdrenalineTotal + day1NoradrenalineTotal + day1
                                     VasopressinTotal
                                 + day1minLactate:day1NoradrenalineTotal
                                 + day1minLactate:day1minMAP
                                 + age:day1maxMAP
                                 + day2minMAP + day2maxMAP
                                 + day2minHR + day2maxHR
                                 + day2maxLactate + day2minLactate
                                 + day2minPotassium + day2maxPotassium
```

```
                                  + day2minSodium + day2maxSodium
                                  + day2minPH + day2maxPH
                                  + day2Ventilated
                                  + day2minCreatinine + day2maxCreatinine
                                  + day2minPaO2FiO2 + day2maxPaO2FiO2
                                  + day2minCRP + day2maxCRP
                                  + day2AdrenalineTotal + day2NoradrenalineTotal + day2
                                      VasopressinTotal
                                  + day2minLactate:day2NoradrenalineTotal
                                  + day2minLactate:day2minMAP
                                  + age:day2maxMAP
                                  + day3minMAP + day3maxMAP
                                  + day3minHR + day3maxHR
                                  + day3maxLactate + day3minLactate
                                  + day3minPotassium + day3maxPotassium
                                  + day3minSodium + day3maxSodium
                                  + day3minPH + day3maxPH
                                  + day3Ventilated
                                  + day3minCreatinine + day3maxCreatinine
                                  + day3minPaO2FiO2 + day3maxPaO2FiO2
                                  + day3minCRP + day3maxCRP
                                  + day3AdrenalineTotal + day3NoradrenalineTotal + day3
                                      VasopressinTotal
                                  + day3minLactate:day3NoradrenalineTotal
                                  + day3minLactate:day3minMAP
                                  + age:day3maxMAP)


formula.day1234.custom<- formula(alive ~
                                   age
                                 + sex
                                 + day1minMAP + day1maxMAP
                                 + day1minHR + day1maxHR
                                 + day1maxLactate + day1minLactate
                                 + day1minPotassium + day1maxPotassium
                                 + day1minSodium + day1maxSodium
                                 + day1minPH + day1maxPH
                                 + day1Ventilated
                                 + day1minCreatinine + day1maxCreatinine
                                 + day1minPaO2FiO2 + day1maxPaO2FiO2
                                 + day1minCRP + day1maxCRP
                                 + day1AdrenalineTotal + day1NoradrenalineTotal + day1
                                     VasopressinTotal
                                 + day1minLactate:day1NoradrenalineTotal
                                 + day1minLactate:day1minMAP
                                 + age:day1maxMAP
                                 + day2minMAP + day2maxMAP
                                 + day2minHR + day2maxHR
                                 + day2maxLactate + day2minLactate
                                 + day2minPotassium + day2maxPotassium
                                 + day2minSodium + day2maxSodium
                                 + day2minPH + day2maxPH
                                 + day2Ventilated
                                 + day2minCreatinine + day2maxCreatinine
                                 + day2minPaO2FiO2 + day2maxPaO2FiO2
                                 + day2minCRP + day2maxCRP
                                 + day2AdrenalineTotal + day2NoradrenalineTotal + day2
                                     VasopressinTotal
                                 + day2minLactate:day2NoradrenalineTotal
                                 + day2minLactate:day2minMAP
                                 + age:day2maxMAP
                                 + day3minMAP + day3maxMAP
                                 + day3minHR + day3maxHR
                                 + day3maxLactate + day3minLactate
                                 + day3minPotassium + day3maxPotassium
                                 + day3minSodium + day3maxSodium
                                 + day3minPH + day3maxPH
                                 + day3Ventilated
                                 + day3minCreatinine + day3maxCreatinine
                                 + day3minPaO2FiO2 + day3maxPaO2FiO2
                                 + day3minCRP + day3maxCRP
                                 + day3AdrenalineTotal + day3NoradrenalineTotal + day3
```

```
                                    VasopressinTotal
                                  + day3minLactate:day3NoradrenalineTotal
                                  + day3minLactate:day3minMAP
                                  + age:day3maxMAP
                                  + day4minMAP + day4maxMAP
                                  + day4minHR + day4maxHR
                                  + day4maxLactate + day4minLactate
                                  + day4minPotassium + day4maxPotassium
                                  + day4minSodium + day4maxSodium
                                  + day4minPH + day4maxPH
                                  + day4Ventilated
                                  + day4minCreatinine + day4maxCreatinine
                                  + day4minPaO2FiO2 + day4maxPaO2FiO2
                                  + day4minCRP + day4maxCRP
                                  + day4NoradrenalineTotal + day4VasopressinTotal + day4
                                      AdrenalineTotal
                                  + day4minLactate:day4NoradrenalineTotal
                                  + day4minLactate:day4minMAP
                                  + age:day4maxMAP)


formula.day12345.custom<- formula(alive ~
                                    age
                                  + sex
                                  + day1minMAP + day1maxMAP
                                  + day1minHR + day1maxHR
                                  + day1maxLactate + day1minLactate
                                  + day1minPotassium + day1maxPotassium
                                  + day1minSodium + day1maxSodium
                                  + day1minPH + day1maxPH
                                  + day1Ventilated
                                  + day1minCreatinine + day1maxCreatinine
                                  + day1minPaO2FiO2 + day1maxPaO2FiO2
                                  + day1minCRP + day1maxCRP
                                  + day1AdrenalineTotal + day1NoradrenalineTotal + day1
                                      VasopressinTotal
                                  + day1minLactate:day1NoradrenalineTotal
                                  + day1minLactate:day1minMAP
                                  + age:day1maxMAP
                                  + day2minMAP + day2maxMAP
                                  + day2minHR + day2maxHR
                                  + day2maxLactate + day2minLactate
                                  + day2minPotassium + day2maxPotassium
                                  + day2minSodium + day2maxSodium
                                  + day2minPH + day2maxPH
                                  + day2Ventilated
                                  + day2minCreatinine + day2maxCreatinine
                                  + day2minPaO2FiO2 + day2maxPaO2FiO2
                                  + day2minCRP + day2maxCRP
                                  + day2AdrenalineTotal + day2NoradrenalineTotal + day2
                                      VasopressinTotal
                                  + day2minLactate:day2NoradrenalineTotal
                                  + day2minLactate:day2minMAP
                                  + age:day2maxMAP
                                  + day3minMAP + day3maxMAP
                                  + day3minHR + day3maxHR
                                  + day3maxLactate + day3minLactate
                                  + day3minPotassium + day3maxPotassium
                                  + day3minSodium + day3maxSodium
                                  + day3minPH + day3maxPH
                                  + day3Ventilated
                                  + day3minCreatinine + day3maxCreatinine
                                  + day3minPaO2FiO2 + day3maxPaO2FiO2
                                  + day3minCRP + day3maxCRP
                                  + day3AdrenalineTotal + day3NoradrenalineTotal + day3
                                      VasopressinTotal
                                  + day3minLactate:day3NoradrenalineTotal
                                  + day3minLactate:day3minMAP
                                  + age:day3maxMAP
                                  + day4minMAP + day4maxMAP
                                  + day4minHR + day4maxHR
                                  + day4maxLactate + day4minLactate
```

```
                                    + day4minPotassium + day4maxPotassium
                                    + day4minSodium + day4maxSodium
                                    + day4minPH + day4maxPH
                                    + day4Ventilated
                                    + day4minCreatinine + day4maxCreatinine
                                    + day4minPaO2FiO2 + day4maxPaO2FiO2
                                    + day4minCRP + day4maxCRP
                                    + day4NoradrenalineTotal + day4VasopressinTotal + day4
                                       AdrenalineTotal
                                    + day4minLactate:day4NoradrenalineTotal
                                    + day4minLactate:day4minMAP
                                    + age:day4maxMAP
                                    + day5minMAP + day5maxMAP
                                    + day5minHR + day5maxHR
                                    + day5maxLactate + day5minLactate
                                    + day5minPotassium + day5maxPotassium
                                    + day5minSodium + day5maxSodium
                                    + day5minPH + day5maxPH
                                    + day5Ventilated
                                    + day5minCreatinine + day5maxCreatinine
                                    + day5minPaO2FiO2 + day5maxPaO2FiO2
                                    + day5minCRP + day5maxCRP
                                    + day5NoradrenalineTotal + day5VasopressinTotal + day5
                                       AdrenalineTotal
                                    + day5minLactate:day5NoradrenalineTotal
                                    + day5minLactate:day5minMAP
                                    + age:day5maxMAP)
```

2.5.2 saveRDSFiles

```
# function to save the model, results and predictions for the resamples for caret and deep
    leanring models

saveRDSFiles ← function(classifier.x, MLmethod, day, iter, cumulative, path, saveClassifier =
    TRUE) {
  if (cumulative == TRUE) {
    cumul ← "cumulative"
  } else {
    cumul ← ""
  }

  for (folder in c("pred", "tuning", "results")) {
    if(!dir.exists(paste(path, folder, MLmethod, sep = "/"))) {
      dir.create(paste(path, folder, MLmethod, sep = "/"), recursive = TRUE)
    }
  }

  if(saveClassifier == TRUE) saveRDS(classifier.x, paste(path,"/tuning/",MLmethod,"/ROC.tuningML.
      ",MLmethod,".day",day,cumul,".iter",iter,".RDS", sep = ""))
  saveRDS(classifier.x$pred, paste(path,"/pred/",MLmethod,"/ROC.tuningML.",MLmethod,".day",day,
      cumul,".iter",iter,".pred.RDS", sep = ""))
  saveRDS(classifier.x$results, paste(path,"/results/",MLmethod,"/ROC.tuningML.",MLmethod,".day",
      day,cumul,".iter",iter,".results.RDS", sep = ""))
}
```

2.5.3 tuneMachineLearningModels

```
# function for building and testing machine learning models with caret and deep learning

library(caret)
library(doMC)
library(mice)
library(dplyr)
source("functions/MLtuning/saveRDSFiles.R") # function to save files
source("functions/MLtuning/formulae.R") # define the required formulae
source("functions/MLtuning/tuneDeepLearningModel.R") # function to mimic caret in tuning a deep
    learning model


```

```
tuneMachineLearningModels ← function(Iter, DeepIter, MLmethods, path.D, seed.list, dayList = data.frame(day = c(1,2,2,3,3,4,4,5,5), cumul = c(F,F,T,F,T,F,T,F,T)), fullData.imput.in, augmentDeaths = FALSE) {

  for (MLmethod in MLmethods) {

    if (MLmethod != "APACHE" & MLmethod != "glm") {
      tune.grid ← get(paste("tune.grid.", MLmethod, sep = ""))
    }

    for(i in 1:nrow(dayList)) {
      day ← dayList$day[i]
      cumul ← dayList$cumul[i]

      if(cumul == T) {
        formula.custom ← get(paste(c("formula.day",1:day,".custom"), collapse = ""))
        formula.simple ← get(paste(c("formula.day",1:day,".simple"), collapse = ""))
      } else {
        formula.custom ← get(paste("formula.day",day,".custom",sep=""))
        formula.simple ← get(paste("formula.day",day,".simple",sep=""))
      }

      trainingData ← filter(fullData.imput.in, icu_duration_days >= day)

      set.seed(42)
      if(MLmethod == "glm") {
        classifier ← train(formula.custom,
                           trainingData,
                           method = MLmethod,
                           metric = "ROC",
                           trControl = train.control,
                           preProcess = c("YeoJohnson", "center", "scale"))

      } else if (MLmethod == "APACHE") {
        classifier ← train(formula.APACHE,
                           trainingData,
                           method = "glm",
                           metric = "ROC",
                           trControl = train.control,
                           preProcess = c("YeoJohnson", "center", "scale"))

      } else if (MLmethod == "DeepNN") {
        tuneDeepLearningModel(tune.grid = tune.grid,
                              dataset_input = trainingData,
                              day = day,
                              cumulative = cumul,
                              seed.list = seed.list,
                              file.path = path.D,
                              iter = DeepIter,
                              verbose = TRUE,
                              no.parallel.cores = no.parallel.cores,
                              tranches.per.core = 2,
                              imputVarTest = FALSE,
                              augmentDeaths = augmentDeaths)

      } else {
        classifier ← train(formula.simple,
                           trainingData,
                           method = MLmethod,
                           metric = "ROC",
                           trControl = train.control,
                           preProcess = c("YeoJohnson", "center", "scale"),
                           tuneGrid = tune.grid)
      }

      if(MLmethod != "DeepNN") {
        saveRDSFiles(classifier.x = classifier, MLmethod = MLmethod, day = day, iter = Iter, cumulative = cumul, path = path.D)
        rm(classifier)
      }
      gc()
    }
```

```
  }
}
```

2.5.4 tuneDeepLearningModel

```
# function to run 20-fold cross-validation of a keras deep learning model when fed a tuning grid

tuneDeepLearningModel ← function(tune.grid, dataset_input, day, cumulative, seed.list, file.path,
     iter, imputVarTest = FALSE, verbose = FALSE, no.parallel.cores, tranches.per.core = 2,
    augmentDeaths = FALSE) {
  set.seed(42)

  # define function to save predictions and results to RDS files
  saveRDSFiles.DeepNN ← function(preds, results, day, iter, cumulative, path) {
    if (cumulative == TRUE) {
      cumul ← "cumulative"
    } else {
      cumul ← ""
    }

    for (folder in c("pred", "tuning", "results")) {
      if(!dir.exists(paste(path, folder, "DeepNN", sep = "/"))) {
        dir.create(paste(path, folder, "DeepNN", sep = "/"), recursive = TRUE)
      }
    }

    saveRDS(preds, paste(path,"/pred/DeepNN/ROC.tuningML.DeepNN.day",day,cumul,".iter",iter,".
        pred.RDS", sep = ""))
    saveRDS(results, paste(path,"/results/DeepNN/ROC.tuningML.DeepNN.day",day,cumul,".iter",iter,
        ".results.RDS", sep = ""))
  }

  # load required packages
  library(devtools)
  library(keras)
  library(caTools)
  library(ggplot2)
  library(caret)
  library(doMC)
  library(pROC)
  library(dplyr)

  # gather appropriate variables
  if(cumulative == FALSE) {
    dataset ← select(dataset_input,
                     alive, icu_duration_days, age, sex,
                     contains(paste("day", day, sep = "")))
  } else {
    dataset ← select(dataset_input,
                     alive, icu_duration_days, age, sex,
                     contains("day1"))
    for(day_add in 2:day) {
      dataset ← cbind(dataset, select(dataset_input,
                                      contains(paste("day", day_add, sep = ""))))
    }
  }

  # filter out values not expected to be present for all days (i.e. patients who leave before day
      of interest)
  dataset ← filter(dataset, icu_duration_days >= day)

  # save a copy of the outcome-labelled dataset and then convert outcome to binary 1/0
  dataset.1 ← dataset
  dataset$alive ← as.factor(ifelse(dataset$alive == "alive", 1, 0))

  ### run a logistic classifier to generate the indices for each fold according to the defined
      seed list (i.e. to match the folds for the models built through caret)
  train.control ← trainControl(method = "LGOCV",
                               number = 20,
                               classProbs = TRUE,
                               verboseIter = FALSE,
```

```
                                seeds = seed.list,
                                savePredictions = "all",
                                summaryFunction = twoClassSummary,
                                returnResamp = "all",
                                p = 0.8)

logistic.classifier <- train(alive ~ age,
                             dataset.1,
                             method = "glm",
                             metric = "ROC",
                             trControl = train.control)

# define function to be parallelised which perfoms 20 fold cross-validation of the deep learning model to produce an output equivalent to caret output
runTuneGridRow <- function(tuneRow, tune.grid, day, cumulative, iter, dataset, logistic.classifier, verbose) {
  library(devtools)
  library(keras)
  library(caTools)
  library(ggplot2)
  library(caret)
  library(pROC)
  library(dplyr)

  # define output dataframes for the tune row
  pred.tuneRow <- data.frame()
  results.tuneRow <- data.frame()

  ### extract tuning parameters from the tune grid
  complexity.multiplier <- tune.grid[tuneRow, "complexity.multiplier"]
  activation.layer_1 <- as.character(tune.grid[tuneRow, "activation.layer_1"])
  activation.layer_2 <- as.character(tune.grid[tuneRow, "activation.layer_2"])
  activation.layer_3 <- as.character(tune.grid[tuneRow, "activation.layer_3"])
  activation.layer_4 <- as.character(tune.grid[tuneRow, "activation.layer_4"])
  activation.layer_5 <- as.character(tune.grid[tuneRow, "activation.layer_5"])
  activation.layer_6 <- as.character(tune.grid[tuneRow, "activation.layer_6"])
  rate.dropout_1 <- tune.grid[tuneRow, "rate.dropout_1"]
  rate.dropout_2 <- tune.grid[tuneRow, "rate.dropout_2"]
  rate.dropout_3 <- tune.grid[tuneRow, "rate.dropout_3"]
  rate.dropout_4 <- tune.grid[tuneRow, "rate.dropout_4"]
  rate.dropout_5 <- tune.grid[tuneRow, "rate.dropout_5"]
  rate.dropout_6 <- tune.grid[tuneRow, "rate.dropout_6"]
  Epochs <- tune.grid[tuneRow, "Epochs"]

  # define a holidng dataframe for the results which will then be used for summary statistics for the overall "results" output
  results.raw <- data.frame()

  # build and test the deep learning model for each fold of 20 folds
  for (fold in 1:20) {
    # name the resample by caret nomenclature
    resample.name <- paste(ifelse(fold <= 9, "Resample0", "Resample"), fold, sep = "")

    # define the row indexes of the traiing and test data
    indexIn <- logistic.classifier$control$index[[fold]]
    indexOut <- logistic.classifier$control$indexOut[[fold]]

    # split into training / test set according to the fold generated by the caret model
    training.df.raw <- dataset[indexIn, ]
    test.df.raw <- dataset[indexOut, ]

    # augment the training data if specified
    if (augmentDeaths == TRUE) {
      training.df.raw <- training.df.raw[c(1:nrow(training.df.raw),
                                           rep(grep(0, training.df.raw$alive), 5)),]
    }

    # preprocess the data to match the caret pre-processing
    suppressWarnings(
    training.df.preProc <- preProcess(training.df.raw, method = c("center", "scale", "YeoJohnson"))
    )
```

```
129
130         training.df ← predict(training.df.preProc, training.df.raw)
131         test.df ← predict(training.df.preProc, test.df.raw)
132
133         # split the dataset into "x" and "y" and prepare the "y" data for training
134         xtrain ← data.matrix(select(training.df, -alive))
135         xtest ← data.matrix(select(test.df, -alive))
136         ytrain ← data.matrix(select(training.df, alive))
137         ytest.raw ← data.matrix(select(test.df, alive))
138
139         ytrain ← to_categorical(ytrain, 3)
140         ytest ← to_categorical(ytest.raw, 3)
141
142         # define the model
143         set.seed(42)
144         model ← keras_model_sequential()
145         model %>%
146           layer_dense(units = 128 * complexity.multiplier, activation = activation.layer_1, input_
                shape = c(ncol(xtrain)), trainable = TRUE) %>%
147           layer_dropout(rate = rate.dropout_1) %>%
148           layer_dense(units = 96 * complexity.multiplier, activation = activation.layer_2) %>%
149           layer_dropout(rate = rate.dropout_2) %>%
150           layer_dense(units = 64 * complexity.multiplier, activation = activation.layer_3) %>%
151           layer_dropout(rate = rate.dropout_3) %>%
152           layer_dense(units = 48 * complexity.multiplier, activation = activation.layer_4) %>%
153           layer_dropout(rate = rate.dropout_4) %>%
154           layer_dense(units = 32 * complexity.multiplier, activation = activation.layer_5) %>%
155           layer_dropout(rate = rate.dropout_5) %>%
156           layer_dense(units = 16 * complexity.multiplier, activation = activation.layer_6) %>%
157           layer_dropout(rate = rate.dropout_6) %>%
158           layer_dense(units = 3, activation = 'softmax')
159
160         # print a summary of the model
161         # summary(model)
162
163         # compile the model
164         model %>% compile(
165           loss = 'binary_crossentropy',
166           optimizer = "Adam",
167           metrics = c('accuracy')
168         )
169
170         # fit the model
171         history ← model %>% fit(
172           xtrain, ytrain,
173           epochs = Epochs, batch_size = 128,
174           validation_split = 0.2,
175           verbose = F
176         )
177
178         # predict the probabilities of the binary outcome on the validation dataset
179         y_pred_prob ← model %>% predict(xtest)
180         roc.y ← pROC::roc(as.vector(ytest.raw), as.vector(y_pred_prob[,3]))
181
182         # predict the binary outcome class (alive/dead) on the validation dataset
183         y_pred ← model %>% predict_classes(xtest)
184         conf.mat ← confusionMatrix(factor(y_pred - 1), factor(ytest[,3]))
185
186         # build the predictions data frame to store all the predictions for the current fold
187         suppressWarnings(
188           pred.fold ← cbind(data.frame(pred = ifelse(y_pred == 2, "alive", "dead"),
189                                        obs = ifelse(as.vector(ytest.raw) == 2, "alive", "dead"),
190                                        dead = as.vector(y_pred_prob[,2]),
191                                        alive = as.vector(y_pred_prob[,3]),
192                                        rowIndex = indexOut),
193                             tune.grid[tuneRow, ],
194                             Resample = resample.name)
195         )
196
197         # generate the results of the current fold
198         results.fold ← data.frame(ROC = pROC::auc(roc.y),
199                                   Sens = conf.mat$byClass["Sensitivity"],
```

```
                                Spec = conf.mat$byClass["Specificity"])

    # add the results and preds of this fold to the list of all folds for this tuning row
    pred.tuneRow ← rbind(pred.tuneRow, pred.fold)
    results.raw ← rbind(results.raw, results.fold)

    # print confirmation of fold completion
    if (verbose == TRUE) print(paste("Completed: Tune row ", tuneRow, " fold ", fold, sep = "")
        )
  }

  # coompile the results of each fold into a single row DF of summarised results for this tune
  results.tuneRow ← cbind(tune.grid[tuneRow, ],
                          ROC = mean(results.raw$ROC),
                          Sens = mean(results.raw$Sens),
                          Spec = mean(results.raw$Spec),
                          ROCSD = sd(results.raw$ROC),
                          SensSD = sd(results.raw$Sens),
                          SpecSD = sd(results.raw$Spec))

  if (verbose == TRUE) print(paste("Day ", day, ifelse(cumulative == TRUE, " cumulative,", ",")
      , " iter ", iter, ": Completed tuning grid row ", tuneRow, " at ", Sys.time(), sep = "")
      )

  # return a list containing two dataframes: 1. The predictions made for each fold on this tune
       row. 2. A one row DF of the results (i.e. ROC/sens/spec) for this tune
  return(list(pred.tuneRow, results.tuneRow))
}

# determine number of models in each tranche and thus number of trances reuired for the given
    tuning grid
tranche.size ← no.parallel.cores * tranches.per.core
no.tranches ← ceiling(nrow(tune.grid)/tranche.size)

for(tranche in 1:no.tranches) {
  # select the list tuning parameters for the current tranche
  tune.grid.subset ← tune.grid[(1 + ((tranche - 1) * tranche.size)) : min(c((tranche * tranche.
      size),nrow(tune.grid))), ]

  # set up parallel cluster
  cl ← makeCluster(no.parallel.cores, outfile = "")

  # run all the tune rows in current tranche in parallel
  outputList ← parLapply(cl = cl,
                         X = 1:nrow(tune.grid.subset),
                         fun = runTuneGridRow,
                         tune.grid = tune.grid.subset,
                         day = day,
                         cumulative = cumulative,
                         iter = iter,
                         dataset = dataset,
                         logistic.classifier = logistic.classifier,
                         verbose = verbose)
  #stop the cluster
  stopCluster(cl)

  # define output dataframes
  pred.out ← data.frame()
  results.out ← data.frame()

  # fill output dataframes
  for(i in 1:length(outputList)) {
    pred.out ← rbind(pred.out, outputList[[i]][[1]])
    results.out ← rbind(results.out, outputList[[i]][[2]])

  }

  # save output dataframes
  saveRDSFiles.DeepNN(preds = pred.out, results = results.out, day = day, iter = ifelse(
      imputVarTest == TRUE, iter, iter + tranche - 1), cumulative = cumulative, path = file.
      path)

```

```
    # print tranche completion confirmation
    print(paste("Day ", day, ifelse(cumulative == TRUE, " cumulative,", ","), ": Completed tuning
          grid tranche ", tranche, " out of ", no.tranches, " at ", Sys.time(), sep = ""))

    # free memory ready for next tranche
    rm(outputList)
    rm(pred.out)
    rm(results.out)
    gc()
  }
}
```

2.6 Interpret results of hyperparameter optimisation

2.6.1 buildPredResDF

```
# function to load and combine results or predicitons into one data frame

library(dplyr)
library(pROC)
library(parallel)
library(tcltk)

buildPredResDF <- function(MLmethod, pred.res, model.df, path) {

  # select models according to input method
  model.df.temp <- filter(model.df, method == MLmethod)

  # for each model, add predictions to the grand prediction output df, with labelling
  for(i in 1:nrow(model.df.temp)) {
    model <- model.df.temp[i,]

    model.pred <- readRDS(model[[paste(pred.res, "Path", sep = "")]])

    model.pred <- mutate(model.pred, ML.method = model$method, day = model$day, iter = model$iter,
          cumulative = model$cumulative)

    if (i == 1) {
      model.predictions.df <- model.pred
    } else {
      model.predictions.df <- rbind(model.predictions.df, model.pred)
    }
  }

  if (pred.res == "results") {
    model.predictions.df$ROC <- 0.5 + abs(0.5 - model.predictions.df$ROC)
  }

  if (!dir.exists(paste(path, "/output/", MLmethod, sep = ""))) dir.create(paste(path, "/output/"
      , MLmethod, sep = ""), recursive = TRUE)
  saveRDS(model.predictions.df, paste(path, "/output/", MLmethod, "/", pred.res, ".", MLmethod, "
      .RDS", sep = ""))
  return(model.predictions.df)
}
```

2.6.2 generateCustomSummary

```
# functions to produce summary statistics for the resampled validation set predictions

library(dplyr)
library(pROC)
library(parallel)
library(tcltk)

# a function to produce a summary of the performance of one fold of one model (i.e. auc,
    sensitivity, specificity etc.)
customSummary <- function(data.obs, data.pred, data.alive, invert = FALSE, switch = FALSE,
    regularise = TRUE) {
  if (regularise == TRUE) {
    # regularise levels
```

```
12      data.obs ← as.character(data.obs)
13      data.obs ← factor(data.obs, levels = c("alive", "dead"))
14
15      data.pred ← as.character(data.pred)
16      data.pred ← factor(data.pred, levels = c("alive", "dead"))
17    }
18
19    # swap predictions if they are the wrong way round
20    if (invert == TRUE) {
21      data.alive ← 1 - data.alive
22    }
23
24    confMat.maxAcc ← caret::confusionMatrix(data = data.pred, reference = data.obs)
25
26    # some resamples fail to converge and so the summary wil be unable to be generated
27    # test for this and only run the summary if it will not throw an error
28    # generate ROC
29    roc.x ← try(pROC::roc(response = ifelse(data.obs == "alive", 0, 1), predictor = data.alive))
30
31    if (class(roc.x) == "try-error") {
32      rocAUC ← NA
33      sens.out.balAcc ← NA
34      spec.out.balAcc ← NA
35      PPV.out.balAcc ← NA
36      NPV.out.balAcc ← NA
37      maxAcc.balAcc ← NA
38      kappa.balAcc ← NA
39      balAcc.balAcc ← NA
40    } else {
41      # get AUC
42      rocAUC ← pROC::auc(roc.x)
43
44      # get coordinates for optimum balanced accuracy
45      coords.x ← pROC::coords(roc.x, "best")
46
47
48      pred.x ← factor(ifelse(data.alive > coords.x[1], "alive", "dead"), levels = c("alive","dead")
            )
49
50      confMat.balanced ← caret::confusionMatrix(data = pred.x, reference = data.obs)
51
52      sens.out.balAcc ← unname(confMat.balanced$byClass["Sensitivity"])
53      spec.out.balAcc ← unname(confMat.balanced$byClass["Specificity"])
54      PPV.out.balAcc ← unname(confMat.balanced$byClass["Pos Pred Value"])
55      NPV.out.balAcc ← unname(confMat.balanced$byClass["Neg Pred Value"])
56      maxAcc.balAcc ← unname(confMat.balanced$overall["Accuracy"])
57      kappa.balAcc ← unname(confMat.balanced$overall["Kappa"])
58      balAcc.balAcc ← unname(confMat.balanced$byClass["Balanced Accuracy"])
59    }
60
61    sens.out.maxAcc ← unname(confMat.maxAcc$byClass["Sensitivity"])
62    spec.out.maxAcc ← unname(confMat.maxAcc$byClass["Specificity"])
63    PPV.out.maxAcc ← unname(confMat.maxAcc$byClass["Pos Pred Value"])
64    NPV.out.maxAcc ← unname(confMat.maxAcc$byClass["Neg Pred Value"])
65    maxAcc.maxAcc ← unname(confMat.maxAcc$overall["Accuracy"])
66    kappa.maxAcc ← unname(confMat.maxAcc$overall["Kappa"])
67    balAcc.maxAcc ← unname(confMat.maxAcc$byClass["Balanced Accuracy"])
68
69    output ← data.frame(AUROC = rocAUC,
70                        Accuracy.maxAccuracy = maxAcc.maxAcc,
71                        Kappa.maxAccuracy = kappa.maxAcc,
72                        Sensitivity.maxAccuracy = sens.out.maxAcc,
73                        Specificity.maxAccuracy = spec.out.maxAcc,
74                        PosPredValue.maxAccuracy = PPV.out.maxAcc,
75                        NegPredValue.maxAccuracy = NPV.out.maxAcc,
76                        BalancedAccuracy.maxAccuracy = maxAcc.balAcc,
77                        Accuracy.balanced = maxAcc.balAcc,
78                        Kappa.balanced = kappa.balAcc,
79                        Sensitivity.balanced = sens.out.balAcc,
80                        Specificity.balanced = spec.out.balAcc,
81                        PosPredValue.balanced = PPV.out.balAcc,
82                        NegPredValue.balanced = NPV.out.balAcc,
```

```
                        BalancedAccuracy.balanced = balAcc.balAcc,
                        stringsAsFactors = FALSE)
  return(output)
}

# wrapper for the above function to apply it to the resampled data generated below
generateCustomSummary ← function(bestPred.df) {
  MLmethod ← bestPred.df$ML.method[1] # get ML method

  resamples.df ← unique(select(bestPred.df, day, bt.Str, cumulative, Resample))

  for (i in 1:nrow(resamples.df)) {
    resample ← resamples.df[i,]
    data ← filter(bestPred.df,
                  day == resample$day,
                  bt.Str == resample$bt.Str,
                  Resample == resample$Resample,
                  cumulative == resample$cumulative)

    invert ← ifelse(MLmethod == "svmRadialWeights", TRUE, FALSE)

    output ← cbind(MLmethod = MLmethod,
                   day = resample$day,
                   cumulative = resample$cumulative,
                   bt.Str = as.character(resample$bt.Str),
                   Resample = resample$Resample,
                   customSummary(data.obs = data$obs,
                                 data.pred = data$pred,
                                 data.alive = data$alive,
                                 invert = invert,
                                 regularise = TRUE))

    if (i == 1) {
      ML.resampleSummary.out ← output
    } else {
      ML.resampleSummary.out ← rbind(ML.resampleSummary.out, output)
    }
  }
  return(ML.resampleSummary.out)
}
```

2.6.3 generateModelDF

```
# function to retrieve the predictions and results from machine learning model output files

library(caret)
library(dplyr)

generateModelDF ← function(path.D = "~/RDSfiles") {
  # generate list of possible classifiers
  combinations ← expand.grid(MLmethod = c("adaboost", "avNNet", "parRF", "svmRadialWeights", "glm
      ", "DeepNN", "APACHE"),
                             days = 0:5,
                             iter = 1:100,
                             cumulative = c("cumulative", ""))

  # output model df is currently empty
  out.model.df.length ← 0
  out.model.df ← data.frame()

  # try each classifier and if it exists, add it to a list
  for (i in 1:nrow(combinations)) {
    # select classifier to try from combinations
    combTry ← combinations[i,]

    #get the right path
    path ← path.D

    # try to load the results (smallest of the three options --> fastest)
    fileExists ← file.exists(paste(path, "/results/", combTry$MLmethod, "/ROC.tuningML.", combTry
        $MLmethod,".day", combTry$day, combTry$cumulative, ".iter", combTry$iter, ".results.RDS"
```

```
          , sep = ""))

    out.model.df.row <- data.frame(method = combTry$MLmethod,
                                   day = combTry$day,
                                   iter = combTry$iter,
                                   classifierPath = paste(path, "/tuning/", combTry$MLmethod, "/ROC.tuningML.", combTry$MLmethod,".day", combTry$day, combTry$cumulative, ".iter", combTry$iter, ".RDS", sep = ""),
                                   predPath = paste(path, "/pred/", combTry$MLmethod, "/ROC.tuningML.", combTry$MLmethod,".day", combTry$day, combTry$cumulative, ".iter", combTry$iter, ".pred.RDS", sep = ""),
                                   resultsPath = paste(path, "/results/", combTry$MLmethod, "/ROC.tuningML.", combTry$MLmethod,".day", combTry$day, combTry$cumulative, ".iter", combTry$iter, ".results.RDS", sep = ""),
                                   stringsAsFactors = FALSE,
                                   cumulative = ifelse(combTry$cumulative == "", FALSE, TRUE))

    if (fileExists) {
      if (out.model.df.length == 0) {
        out.model.df <- out.model.df.row
        out.model.df.length <- out.model.df.length + 1
      } else {
        out.model.df <- rbind(out.model.df, out.model.df.row)
        out.model.df.length <- out.model.df.length + 1
      }
    }
  }

  return(out.model.df)
}
```

2.6.4 getBestTune

```
# function to find the best performing tuning parameters for each day for a machine learning method

library(dplyr)
source("functions/MLtuning_output/getTuningParams.R")

getBestTune <- function(MLmethod) {
  results.df <- get(paste("ML.results.df.", MLmethod, sep = ""))

  tuning.params <- getTuningParams(results.df, "results")

  bestPreds <- results.df %>% group_by(day, cumulative) %>% summarise(ROC.best = max(ROC))

  bestTune <- as.data.frame(results.df[results.df$ROC %in% bestPreds$ROC.best, c("day", tuning.params,"ROC", "iter", "cumulative")])

  return(bestTune)
}
```

2.6.5 getBestTunePreds

```
# function to retrieve the predictions made for the validation dataset from the best performing tuning parameters for a given machine learning method

library(dplyr)
library(parallel)
library(tcltk)

source("functions/MLtuning_output/getBestTune.R")

getBestTunePreds <- function(MLmethod, path) {
  # get the tuning parameters for the best tune for each day
  bestTune <- getBestTune(MLmethod = MLmethod)

```

```
13    # find the number of tuning parameters
14    tune.no ← ncol(bestTune) - 4
15
16    # find the names of the tuning parameters
17    tuners ← colnames(bestTune)[2 : (1 + tune.no)]
18
19    # correct labelling of complexity in DeepNN to standard number of digits
20    if(MLmethod == "DeepNN") bestTune$complexity.multiplier ← as.character(format(as.numeric(
          bestTune$complexity.multiplier), nsmall = 4))
21
22    # collapse the tuning parameters for the best tunes to lookup
23    if (tune.no > 1) {
24      bestTune$bt.Str ← apply(bestTune[ , tuners] , 1 , paste , collapse = "-" )
25    } else {
26      bestTune$bt.Str ← bestTune[ , tuners]
27    }
28
29    pb ← tkProgressBar(title = "Progress", label = MLmethod,
30                       min = 0, max = nrow(bestTune), initial = 0, width = 300)
31
32    # load the predictions for the best tunes only and add the resampled predictions to an output
          data frame
33    out.df ← data.frame()
34    for (i in 1:nrow(bestTune)) {
35      tune ← bestTune[i,]
36      load.df ← readRDS(paste(path, "/pred/", MLmethod, "/ROC.tuningML.", MLmethod, ".day", tune$
            day, ifelse(tune$cumulative, "cumulative", ""), ".iter", tune$iter, ".pred.RDS", sep = "
            "))
37      newPred.df ← mutate(load.df, ML.method = MLmethod, day = tune$day, iter = tune$iter,
            cumulative = tune$cumulative)
38
39      # correct labelling of complexity in DeepNN to standard number of digits
40      if(MLmethod == "DeepNN") newPred.df$complexity.multiplier ← as.character(format(as.numeric(
            newPred.df$complexity.multiplier), nsmall = 4))
41
42      if (tune.no > 1) {
43        if(nrow(newPred.df) > 3000000) {
44          cl ← makeCluster(detectCores() - 1)
45          newPred.df$bt.Str ← parApply(cl, newPred.df[ , tuners] , 1 , paste , collapse = "-" )
46          stopCluster(cl)
47        } else {
48          newPred.df$bt.Str ← apply(newPred.df[ , tuners] , 1 , paste , collapse = "-" )
49        }
50      } else {
51        newPred.df$bt.Str ← newPred.df[ , tuners]
52      }
53
54      out.df ← rbind(out.df, filter(newPred.df,
55                                    bt.Str == tune$bt.Str,
56                                    day == bestTune[i,"day"],
57                                    iter == bestTune[i,"iter"],
58                                    cumulative == bestTune[i,"cumulative"]))
59
60      setTkProgressBar(pb, i)
61    }
62    close(pb)
63    return(out.df)
```

2.6.6 getTuningParams

```
1  # determine the tuning functions for the machine learning method in question
2
3  getTuningParams ← function(input.df, type = "results") {
4    if (type == "results") {
5      return(colnames(input.df)[1:grep("ROC", colnames(input.df))[1]-1])
6    } else if (type == "pred") {
7      return(colnames(input.df)[(grep("rowIndex", colnames(input.df))[1] + 1) : (grep("Resample",
           colnames(input.df))[1] - 1)])
8    }
9  }
```

2.7 Building final models across all imputations

2.7.1 imputationVariationModels

```
# function to build the final model for each of nine imputations for each method and day

library(caret)
library(doMC)
library(mice)
library(dplyr)
source("functions/MLtuning/saveRDSFiles.R") # function to save files
source("functions/MLtuning/formulae.R") # define the required formulae
source("functions/MLtuning_output/getBestTune.R")
source("functions/MLtuning/tuneDeepLearningModel.R")

imputationVariationModels <- function(MLmethods, path.impVar, dayList = data.frame(day = c(1,2,2,3,3,4,4,5,5), cumul = c(F,F,T,F,T,F,T,F,T)), augmentDeaths = FALSE) {
  iter.base <- "IMPVAR"

  for (MLmethod in MLmethods) {

    best.tunes <- getBestTune(MLmethod)

    tuning.params <- colnames(best.tunes)[(grep("day", colnames(best.tunes)) + 1):(grep("ROC", colnames(best.tunes)) - 1)]

    for(i in 1:nrow(dayList)) {
      day <- dayList$day[i]
      cumul <- dayList$cumul[i]
      tune.grid <- best.tunes %>% filter(day == dayList$day[i], cumulative == cumul) %>% select(one_of(tuning.params))

      if(cumul == T) {
        formula.custom <- get(paste(c("formula.day",1:day,".custom"), collapse = ""))
      } else {
        formula.custom <- get(paste("formula.day",day,".custom",sep=""))
      }


      # run model for best tune on each day
      for(imput.number in 1:9) {
        iter <- paste(iter.base, imput.number, sep = "")
        imputedSet <- mice::complete(imputedData.all, imput.number)

        trainingData <- filter(imputedSet, icu_duration_days >= day)

        set.seed(42)
        if(MLmethod == "glm") {
          classifier <- train(formula.custom,
                              trainingData,
                              method = MLmethod,
                              metric = "ROC",
                              trControl = train.control,
                              preProcess = c("YeoJohnson", "center", "scale"))

        } else if (MLmethod == "APACHE") {
          classifier <- train(formula.APACHE,
                              trainingData,
                              method = "glm",
                              metric = "ROC",
                              trControl = train.control,
                              preProcess = c("YeoJohnson", "center", "scale"))

        } else if (MLmethod == "DeepNN") {
          tuneDeepLearningModel(tune.grid = tune.grid,
                                dataset_input = trainingData,
                                day = day,
                                cumulative = cumul,
                                seed.list = seed.list,
                                file.path = path.impVar,
                                iter = iter,
```

```
                                verbose = TRUE,
                                no.parallel.cores = 1,
                                tranches.per.core = 1,
                                imputVarTest = TRUE,
                                augmentDeaths = augmentDeaths)

        } else {
          classifier ← train(formula.custom,
                             trainingData,
                             method = MLmethod,
                             metric = "ROC",
                             trControl = train.control,
                             preProcess = c("YeoJohnson", "center", "scale"),
                             tuneGrid = tune.grid)
        }

        if(MLmethod != "DeepNN") {
          saveRDSFiles(classifier.x = classifier, MLmethod = MLmethod, day = day, iter = iter,
               cumulative = cumul, path = path.impVar, saveClassifier = FALSE)
        }
      }
    }
  }
}
```

2.7.2 getImputationResults

```
# function to load the results or predictions of the models for each imputation

library(tcltk)

getImputationResults ← function(MLmethod, imputationPath, output.type = "results", invert = FALSE
    ) {
  # generate list of possible classifiers
  combinations ← expand.grid(day = 1:5,
                             iter = 1:9,
                             cumulative = c("cumulative", ""))

  # output model df is currently empty
  out.model.df.length ← 0
  out.model.df ← data.frame()

  # set up progress bar
  pb ← tkProgressBar(title = MLmethod, label = "Reading RDS files...",
                     min = 0, max = nrow(combinations), initial = 0, width = 300)

  # try each classifier and if it exists, add it to a list
  for (i in 1:nrow(combinations)) {
    # select classifier to try from combinations
    combTry ← combinations[i,]

    #get the right path
    path ← imputationPath

    # generate path for this model
    model.path ← paste(path, "/",output.type,"/", MLmethod, "/ROC.tuningML.", MLmethod,".day",
         combTry$day, combTry$cumulative, ".iterIMPVAR", combTry$iter, ".", output.type, ".RDS",
         sep = "")

    # try to load the results (smallest of the three options --> fastest)
    if(file.exists(model.path)) {
      model.results ← readRDS(model.path)

      out.model.df.row ← data.frame(MLmethod = MLmethod,
                                    day = combTry$day,
                                    cumulative = ifelse(combTry$cumulative == "cumulative", TRUE
                                         , FALSE),
                                    imputation = combTry$iter,
                                    model.results)

      if (out.model.df.length == 0) {
```

```
          out.model.df ← out.model.df.row
          out.model.df.length ← out.model.df.length + 1
        } else {
          out.model.df ← rbind(out.model.df, out.model.df.row)
          out.model.df.length ← out.model.df.length + 1
        }
      }

      setTkProgressBar(pb, i)
    }
    close(con = pb)

    if (invert == TRUE) out.model.df$ROC ← 1 - out.model.df$ROC

    return(out.model.df)
  }
```

2.7.3 generateFoldedImputationSummaries

```
  # function to produce summary statistics from a dataframe of resample results across all
      imputations

  library(tcltk)
  library(dplyr)
  library(rlang)

  source("functions/MLtuning_output/generateCustomSummary.R")

  generateFoldedImputationSummaries ← function(imputationPredsDF, invert = FALSE, switch = FALSE) {
    # make list of all resamples
    individual.resamples.list ← unique(select(imputationPredsDF, MLmethod, day, cumulative,
        imputation, Resample))

    MLmethod ← as.character(imputationPredsDF[1,"MLmethod"])

    pb ← tkProgressBar(title = MLmethod, label = "Generating resampling statistics...",
                       min = 0, max = nrow(individual.resamples.list), initial = 0, width = 300)

    resample.list ← data.frame()
    for(i in 1:nrow(individual.resamples.list)) {
      IRL.element ← individual.resamples.list[i,]
      preds.df ← filter(imputationPredsDF,
                        day == IRL.element$day,
                        cumulative == IRL.element$cumulative,
                        imputation == IRL.element$imputation,
                        Resample ==  IRL.element$Resample)

      resampleResults.row ← cbind(MLmethod = IRL.element$MLmethod,
                                  day = IRL.element$day,
                                  cumulative = IRL.element$cumulative,
                                  imputation = IRL.element$imputation,
                                  Resample =  IRL.element$Resample,
                                  customSummary(data.obs = preds.df$obs,
                                                data.pred = preds.df$pred,
                                                data.alive = preds.df$alive,
                                                invert = invert,
                                                switch = switch,
                                                regularise = TRUE))



      resample.list ← rbind(resample.list, resampleResults.row)

      setTkProgressBar(pb, i)

    }
    close(con = pb)

    return(resample.list)
  }
```

2.8 Explore predictions

2.8.1 addVariableToExplorePredsDF

```
# looks up the values of the input variable, or all variables, for the correct imputation, for the correct admission index and returns the input dataframe with the new variable attached

library(dplyr)
library(tcltk)

addVariableToExplorePredsDF ← function(explorePreds.df, variableName) {
  out.df ← data.frame()

  # set up progress bar
  pb ← tkProgressBar(title = "Add variable", label = "Adding variables...",
                     min = 0, max = 9 * 5, initial = 0, width = 300)

  for (imput in 1:9) {
    imputedSet ← completeCustom(imputedData.all, imput)

    imput.df ← data.frame()
    for (day.in in 1:5) {
      dataSet.filtered ← filter(imputedSet, icu_duration_days >= day.in)

      filtered.df ← filter(explorePreds.df, imputation == imput, day == day.in)

      imput.df ← rbind(imput.df,
                       cbind(filtered.df,
                             dataSet.filtered[filtered.df$rowIndex,variableName]))
      setTkProgressBar(pb, (imput * 5) - 5 + day.in)
    }
    out.df ← rbind(out.df, imput.df)
  }

  close(con = pb)

  colnames(out.df)[(length(out.df) - (length(variableName) - 1)): length(out.df)] ← variableName

  return(out.df)
}


addAllVariablesToExplorePredsDF ← function(explorePreds.df) {
  out.df ← data.frame()

  # set up progress bar
  pb ← tkProgressBar(title = "Add variable", label = "Adding variables...",
                     min = 0, max = 9 * 5, initial = 0, width = 300)

  for (imput in 1:9) {
    imputedSet ← completeCustom(imputedData.all, imput)

    imput.df ← data.frame()
    for (day.in in 1:5) {
      dataSet.filtered ← filter(imputedSet, icu_duration_days >= day.in)

      filtered.df ← filter(explorePreds.df, imputation == imput, day == day.in)

      imput.df ← rbind(imput.df,
                       cbind(filtered.df,
                             dataSet.filtered[filtered.df$rowIndex,]))
      setTkProgressBar(pb, (imput * 5) - 5 + day.in)
    }
    out.df ← rbind(out.df, imput.df)
  }

  close(con = pb)

  return(out.df)
}
```

2.9 Graph plotting functions

2.9.1 generateRootDir

```
# Generates standardised directory for graph plotting which updates based on the date

generateRootDir ← function(directory) {
  rootDir ← file.path("./Graphs",Sys.Date())
  dir.create(rootDir, showWarnings = FALSE, recursive = TRUE)
  rootDir ← file.path("./Graphs",Sys.Date(),directory)
  dir.create(rootDir, showWarnings = FALSE, recursive = TRUE)
  return(rootDir)
}
```

2.9.2 plotMiscGraph

```
# set up to plot a generic graph

plotMiscGraph ← function(dirName, graphName, width = 6000, height = 3000, ptSize = 14) {
  rootDir ← generateRootDir(directory = dirName)
  fileName ← paste(rootDir,
                   "/",graphName,
                   "_",
                   Sys.Date(),
                   ".png",
                   sep = "")

  png(file = fileName,
      width = width,
      height = height,
      res = 300,
      pointsize = ptSize)
}
```

2.9.3 Plot patient outcome waterfall

```
# Script to plot waterfall graph of admission length

library(dplyr)

plot.summary ← cleanData.all %>% group_by(icu_duration_days) %>% summarise(tot = n(), noAlive =
    sum(alive == "alive"), noDead = sum(alive != "alive"))

plot.summary$totLeft ← NA
plot.summary$missingClassifier ← NA


for (i in 1:nrow(plot.summary)) {
  plot.summary$totLeft[i] ← nrow(cleanData.all) - sum(plot.summary$tot[1:i])
}
plot.summary ← rbind(list(icu_duration_days = c(-2,-1,0),
                          tot = c(0,0,0),
                          noAlive = c(0,0,0),
                          noDead = c(0,0,0),
                          totLeft = c(nrow(dataSet),nrow(cleanData.all),nrow(cleanData.all)),
                          missingClassifier = c(NA, nrow(dataSet) - nrow(cleanData.all),0)),
                     plot.summary)


plot.summary.mut ← transmute(plot.summary, day = icu_duration_days, remaining = totLeft, alive =
    noAlive, dead = noDead, issingClassifier = missingClassifier)
plot.df.full ← t.data.frame(plot.summary.mut)
colnames(plot.df.full) ← plot.df.full[1,]
colnames(plot.df.full)[1:3] ← c("total","","")
plot.df ← plot.df.full[c(2:5),1:33]
plot.df ← rbind(plot.df, holdRow = 0)
plot.df ← plot.df[c(5,1,2,3,4),]

plot.df ← plot.df[,c(1:2, 4:ncol(plot.df))]
```

```
32
33  for(i in c(1,3,7, seq(12, 102, 10))) {
34    if(ncol(plot.df) >= i) {
35      plot.df[1,i] ← plot.df[2,i]
36      plot.df[2,i] ← 0
37    }
38  }
39
40  plotMiscGraph("Descriptive", "patientDecay_30", 4000, 2200)
41  barplot(plot.df, xlab="Day of admission", col=c("darkgrey","white","#A3C1AD","darkblue", "darkred
        "), border = NA, las = 1, ylim = c(0,26000), width = 0.9, space = 1/9, xlim = c(0,ncol(plot.
        df)+1))
42  legend("topright", inset = 0.03, legend = c("missing classifier","discharged alive", "deceased",
        "remain on ICU"), col = c("darkred", "#A3C1AD","darkblue","grey"), bty = "n", pch = 15)
43  box()
44
45  text(x = 1.15, y = 23300, labels = "22,524", pos = 3, adj = 0, srt = 45)
46  text(x = 3.15, y = 22700, labels = "21,911", pos = 3, adj = 0, srt = 45)
47  text(x = 7.6, y = 8550, labels = "6,916 (32%)", pos = 3, adj = 0, srt = 45)
48  text(x = 12.6, y = 4750, labels = "2,909 (13%)", pos = 3, adj = 0, srt = 45)
49  text(x = 22.5, y = 2500, labels = "1,092 (5%)", pos = 3, adj = 0, srt = 45)
50  text(x = 32.2, y = 1600, labels = "489 (2%)", pos = 3, adj = 0, srt = 45)
51  dev.off()
```

2.9.4 plotDescriptiveGraphs & plotBeanplots

```
1   # Plots histograms and beanplots to visually descibe the input dataset
2
3   library(beanplot)
4   library(readxl)
5   library(tidyr)
6   library(dplyr)
7
8   source("functions/graph_plotting/generateRootDir.R")
9
10  plotDescriptiveGraphs ← function(inputData, directory, exclusions = NULL, height = 6000, width =
        3000, titled = TRUE, legend = TRUE, ptSize = 14) {
11
12    ### Load the list of variables to be plotted
13    continuousVariableDF ← filter(allVariablesDF, variableType %in% c("continuous", "integer"))
14
15    if (!is.null(exclusions)) {
16      continuousVariableDF ← continuousVariableDF[!continuousVariableDF$variableSubtitle %in%
            exclusions,]
17    }
18
19
20    ### Create Graph directory
21    rootDir ← generateRootDir(directory)
22
23    ###  histogram of age
24    fileName ← paste(rootDir,"/age_Hist_",
25                     Sys.Date(),
26                     ".png",
27                     sep = "")
28    png(file = fileName,
29        width = width,
30        height = height,
31        res = 300,
32        pointsize = ptSize)
33    par(mfrow = c(1,1))
34
35    hist(inputData$age,
36         col = c("darkblue"),
37         xlab = "Age",
38         main = "Histogram of Age",
39         xlim = c(0,110),
40         xaxt = 'n')
41
42
43    axis(1, at = seq(0, 120, by = 5), las=2)
```

```
44
45    dev.off()
46
47    ###  histogram of icu_duration
48    fileName ← paste(rootDir,"/icuDuration_Hist_",
49                     Sys.Date(),
50                     ".png",
51                     sep = "")
52    png(file = fileName,
53        width = width,
54        height = height,
55        res = 300,
56        pointsize = ptSize)
57    par(mfrow = c(1,1))
58
59    barplot(table(inputData$icu_duration_days),
60         col = c("darkblue"),
61         ylim = c(0,6000),
62         ylab = "Frequency",
63         xlab = "ICU duration (days)",
64         main = "Histogram of ICU duration")
65
66    dev.off()
67
68
69    ###  histogram of alive or dead
70    fileName ← paste(rootDir,"/aliveDead_Hist_",
71                     Sys.Date(),
72                     ".png",
73                     sep = "")
74    png(file = fileName,
75        width = width,
76        height = height,
77        res = 300,
78        pointsize = ptSize)
79    par(mfrow = c(1,1))
80
81    plot(inputData$alive,
82         col = c("#A3C1AD", "darkblue"),
83         ylim = c(0,21000),
84         ylab = "Frequency",
85         xlab = "Classifier",
86         main = "Histogram of outcome classifier")
87
88    dev.off()
89
90    ###  Plot the Beanplots
91
92    plotList ← unique(select(continuousVariableDF, variableSubtitle, graphTitle, type, units))
93
94    for(i in 1:nrow(plotList)) {
95      fileName ← paste(rootDir,"/",
96                       plotList$variableSubtitle[i],
97                       "_Beanplot_",
98                       Sys.Date(),
99                       ".png",
100                      sep = "")
101     png(file = fileName,
102         width = width,
103         height = height,
104         res = 300,
105         pointsize = ptSize)
106     par(mfrow = c(1,1))
107
108     plotBeanplots(variable = plotList$variableSubtitle[i],
109                   plotName = plotList$graphTitle[i],
110                   type = plotList$type[i],
111                   plotUnits = plotList$units[i],
112                   inputData = inputData,
113                   legend = legend,
114                   titled = titled)
115
```

```
116       dev.off()
117     }
118
119
120     ### Plot ventilated data
121     aliveID ← dataSet$alive_dead_icu == "A"
122     deadID ← dataSet$alive_dead_icu != "A"
123
124     #pull out ventilated data into a data frame based on the classifier
125     generateVentTable ← function(classifierList) {
126       #pull out ventilated data for the classifier
127       tempVentData ← data.frame(summary(inputData[classifierList,grepl("Ventilated",colnames(
              inputData))]))
128
129       #reformat dataframe
130       tempVentData$Type ← c("No","Yes","NA")
131       tempVentData$Freq ← lapply(tempVentData$Freq, gsub, pattern = "Yes|No|:|NA's", replacement =
              "")
132       tempVentData$Freq ← as.integer(lapply(tempVentData$Freq, trimws, which = "both"))
133       tempVentData ← tempVentData[,2:length(tempVentData)]
134       colnames(tempVentData) ← c("Day","Freq","Type")
135
136       #return vetilation table
137       return(tempVentData)
138     }
139
140     #function that returns the percentages of ventilated patients based on the classified data
          frame
141     getClassifierPercs ← function(tempVentData) {
142       #return % of ventilated for each day
143       return(tempVentData$Freq[tempVentData$Type == "Yes"] / (tempVentData$Freq[tempVentData$Type
              == "Yes"] + tempVentData$Freq[tempVentData$Type == "No"]))
144     }
145
146     getVentilatedPercs ← function(tempVentDataAlive, tempVentDataDead, ventilated) {
147       tempVentDataAlive$Freq[tempVentDataDead$Type == ventilated] / (tempVentDataDead$Freq[
              tempVentDataAlive$Type == ventilated] + tempVentDataAlive$Freq[tempVentDataDead$Type ==
              ventilated])
148     }
149
150     ventData1 = data.frame(list(c(getVentilatedPercs(generateVentTable(aliveID),generateVentTable(!
          aliveID),"Yes"), getVentilatedPercs(generateVentTable(aliveID),generateVentTable(!aliveID)
          ,"No")),
151                                 c(replicate(5,"Ventilated"),replicate(5,"Not Ventilated")),
152                                 c("Day 1", "Day 2", "Day 3", "Day 4", "Day 5")))
153
154     colnames(ventData1) = c("Percent_Alive", "Ventilated", "Day")
155
156     ventData2 = data.frame(list(c(getClassifierPercs(generateVentTable(aliveID)),
          getClassifierPercs(generateVentTable(!aliveID))),
157                                 c(replicate(5,"Alive"),replicate(5,"Dead")),
158                                 c("Day 1", "Day 2", "Day 3", "Day 4", "Day 5")))
159
160     colnames(ventData2) = c("Percent_Ventilated", "Classifier", "Day")
161
162     #draw the graphs
163     library(lattice)
164
165     fileName ← paste(rootDir,
166                      "/Ventilated",
167                      "_Barplot_V1_",
168                      Sys.Date(),
169                      ".png",
170                      sep = "")
171     png(file = fileName,
172         width = width,
173         height = height,
174         res = 300,
175         pointsize = 12)
176     par(mfrow = c(1,1))
177
178     barchart(Percent_Alive ~ Day,
```

```
179             groups=Ventilated,
180             ventData1,
181             auto.key = list(columns = 3),
182             par.settings = list(
183               superpose.polygon=list(col=c("darkgrey","darkred"), border="black"),
184               strip.background=list(col="red"),
185               strip.border=list(col="black")),
186             ylim = c(0,1),
187             main="Percentage dead based on ventilation status")
188
189   dev.off()
190
191   fileName ← paste(rootDir,
192                    "/Ventilated",
193                    "_Barplot_V2_",
194                    Sys.Date(),
195                    ".png",
196                    sep = "")
197   png(file = fileName,
198       width = width,
199       height = height,
200       res = 300,
201       pointsize = 12)
202   par(mfrow = c(1,1))
203
204   barchart(Percent_Ventilated ~ Day,
205             groups=Classifier,
206             ventData2,
207             auto.key = list(columns = 3),
208             par.settings = list(
209               superpose.polygon=list(col=c("#A3C1AD","darkblue"), border="black"),
210               strip.background=list(col="red"),
211               strip.border=list(col="black")),
212             ylim = c(0,1),
213             main="Percentage ventilated for each classifier")
214
215   dev.off()
216 }
217
218 plotBeanplots ← function(variable, plotName, plotUnits, type, inputData, legend = TRUE, titled =
      TRUE, splitName = "alive", splitLevel1 = "alive", splitLevel2 = "dead") {
219
220   #pull out variable i into a long dataframe
221   tempDataFrame = gather(select(inputData, contains(variable)))
222   tempDataFrame$splitter ← inputData[[splitName]]
223
224   #remove variable from names of days and reconstruct naming
225   tempDataFrame$key = gsub(variable, "", tempDataFrame$key)
226   tempDataFrame$key = gsub("day", "Day ", tempDataFrame$key)
227
228   level.1.DF ← filter(tempDataFrame, splitter == splitLevel1)
229   level.2.DF ← filter(tempDataFrame, splitter == splitLevel2)
230
231   #set up beanplot
232   if (type == "time") {
233     xlimits ← c(0,6)
234   } else {
235     xlimits ← c(0,2)
236   }
237
238   ylimits ← c(min(tempDataFrame$value, na.rm = TRUE), max(tempDataFrame$value, na.rm = TRUE))
239
240   #set plotting units
241   plotUnits ← ifelse(!is.na(plotUnits), paste(" / ", plotUnits, sep = ""), "")
242
243   #plot alive/level1
244   beanplot(value~key, level.1.DF,
245             side = "first",
246             col=c("#A3C1AD","#A3C1AD", "lightgrey"),
247             xlim = xlimits,
248             ylim = ylimits,
249             main = ifelse(titled == TRUE,
```

```
                          paste("Beanplot showing distribution of",
                                plotName,
                                "by vital status at discharge"), NA),
            ylab = paste(plotName, plotUnits, sep = ""),
            log = "",
            what = c(1,1,0,1),
            las = 1)

  #plot deceased/level2
  beanplot(value~key, level.2.DF,
            side = "second",
            col=c("darkblue","darkblue", "lightgrey"),
            add = TRUE,
            what = c(1,1,0,1))

  #add arithmetic mean lines
  if (type == "time") {
    for (day in 1:5) {
      meanVal <- mean(level.1.DF$value[level.1.DF$key == paste("Day ",day,sep="")], na.rm = TRUE)
      lines(x = c(day, day - 0.5),
            y = c(meanVal,meanVal),
            col = "black",
            lwd = 2)

      meanVal <- mean(level.2.DF$value[level.2.DF$key == paste("Day ",day,sep="")], na.rm = TRUE)
      lines(x = c(day, day + 0.5),
            y = c(meanVal,meanVal),
            col = "black",
            lwd = 2)
    }
  } else {
    meanVal <- mean(level.1.DF$value, na.rm = TRUE)
    lines(x = c(1, 1 - 0.5),
          y = c(meanVal,meanVal),
          col = "black",
          lwd = 2)

    meanVal <- mean(level.2.DF$value, na.rm = TRUE)
    lines(x = c(1, 1 + 0.5),
          y = c(meanVal,meanVal),
          col = "black",
          lwd = 2)
  }

  #add a legend
  if(legend == TRUE) legend('topright', fill=c('#A3C1AD','darkblue'), legend= c(splitLevel1,
      ifelse(splitLevel2 == "dead", "deceased", splitLevel2)), bty ="n")
}
```

2.9.5 manualDensityPlots

```
# function for plotting the density plots showing original values vs. imputations

library(mice)
library(parallel)
library(dplyr)

manualDensityPlots <- function(mids, cleanData, titles = TRUE, minititles = TRUE, plotDir = "
    densityPLots_manual", width = 4000, height = 2000, minMax = FALSE) {
  for(title in unique(filter(allVariablesDF, outlierRemoval == TRUE, day > 0, !(variableTitle %in
      % c("NoradrenalineTotal", "AdrenalineTotal", "VasopressinTotal")))$variableTitle)) {
    varDF <- allVariablesDF %>% filter(variableTitle == title) %>% arrange(variableSubtitle)

    maxVars <- varDF[1:(nrow(varDF)/2),]$variable
    minVars <- varDF[(1 + nrow(varDF)/2):nrow(varDF),]$variable

    plotMiscGraph(plotDir,
                  paste("density_manual_", title, ifelse(minMax == FALSE, "", paste("_", minMax,
                      sep = "")), sep = ""),
                  width = width,
                  height = height)
```

```
18
19      # set plotting grid according to whether min/max of both are being plotted
20      if (minMax == FALSE) {
21        par(mfrow = c(2,5), oma = c(0, 0, ifelse(titles == TRUE, 3, 0), 0))
22      } else {
23        par(mfrow = c(1,5), oma = c(0, 0, ifelse(titles == TRUE, 3, 0), 0))
24      }
25
26      min.max <- ifelse(minMax == FALSE,c("min", "max"), minMax)
27
28      for(minMax in min.max) {
29        temp.vars <- get(paste(minMax,"Vars", sep = ""))
30        for (day in 1:(nrow(varDF)/2)) {
31
32
33          for (i in 1:7) {
34            compl.full <- mice::complete(mids,i)
35
36            # find values which shouldn't exist
37            naPos <- compl.full$icu_duration_days < day
38            wherePos <- as.data.frame(mids$where)[[temp.vars[day]]]
39
40            imputedPoints <- wherePos & !naPos
41
42            # find density values without extroneous values
43            d.x <- density(compl.full[imputedPoints, temp.vars[day]])
44
45            # find maximum density and add to list of peak values
46            if (i == 1) {
47              peak.list <- max(d.x$y)
48            } else {
49              peak.list <- c(peak.list, max(d.x$y))
50            }
51
52            assign(paste("d", i, sep = "."), d.x)
53          }
54
55
56          # calculate densities for original distribution
57          d <- density(cleanData[[temp.vars[day]]][!is.na(cleanData[[temp.vars[day]]])])
58
59          # find highest peak to determine plotting size
60          peak.list <- c(peak.list, max(d$y))
61          peak <- max(peak.list)
62
63          # plot the density plot for the originaldata on an appropriately sized plot
64          if (minititles == FALSE) {
65            par(mgp = c(2,0.75,0), mar = c(3,3,0.75,0.5))
66            axis.cex <- 0.75
67          } else {
68            axis.cex <- 1
69          }
70
71          plot(d,
72               col = "darkblue",
73               xlab = paste("Day", day, sep = " "),
74               ylab = ifelse(day == 1, "Density", ""),
75               main = ifelse(minititles == TRUE, temp.vars[day], NA),
76               ylim = c(0, peak * 1.3),
77               font.lab = 2,
78               cex.lab = ifelse(minMax == FALSE, 1.3, 1.2),
79               cex.axis = axis.cex)
80
81          # plot the imputed density plots
82          for(i in 1:7) {
83            lines(get(paste("d", i, sep = ".")), col = "#A3C1AD", lwd = 0.5)
84          }
85
86          # add the original plot line again over the top of the imputed ones
87          lines(d, col = "darkblue")
88
89          # add text to show the quantities of original and imputed data
```

```
90          text(y = 1.275 * peak, x = max(d$x), pos = 2, labels = paste("Original: ", format(sum(!
                wherePos), big.mark = ",", scientific = FALSE), sep = ""), col = "darkblue", cex = 1
                .15)
91          text(y = 1.15 * peak, x = max(d$x), pos = 2, labels = paste("Imputed: ", format(sum(
                imputedPoints), big.mark = ",", scientific = FALSE), sep = ""), col = "#A3C1AD", cex
                 = 1.15)
92        }
93      }
94
95      # add a title if requested
96      if (titles == TRUE) mtext(paste("Density plot of existing (dark blue) vs imputed (light blue)
              data for ", title, sep = ""), outer = TRUE, cex = 1.5)
97
98      dev.off()
99    }
100 }
```

2.9.6 plotTuningGraphs

```
1   # Function to plot tuning results
2
3   plotTuningGraphs ← function() {
4     library(dplyr)
5
6     line.cols ← c("darkred", "darkblue", "darkgreen", "pink", "orange", "blue", "green", "purple",
          "red", "black", "grey", "yellow", "lilac", "violet", "brown")
7     pch.list ← c(1,2,3,4,5,6,7,8,9,10,11,12,13,14,15,16,17,18)
8
9     ### SVM Radial
10
11    tuning.plot.list ← unique(select(ML.results.df.svmRadialWeights, day, cumulative))
12
13    for (i in 1:nrow(tuning.plot.list)) {
14      day.x ← tuning.plot.list[i,]$day
15      cumul ← tuning.plot.list[i,]$cumulative
16
17      plotMiscGraph("MLtuning", paste("svmRadial_tuning_day", day.x, ifelse(cumul == TRUE, "_
            cumulative", ""), sep = ""), 4000, 2000)
18
19      # filter for the correct range of days, 3 or 5
20      ML.results.df.svmRadial.filtered ← filter(ML.results.df.svmRadialWeights, cumulative == cumul
            , day == day.x)
21
22
23      nGraphs ← length(unique(ML.results.df.svmRadial.filtered$sigma)) + 1
24      width ← ceiling(sqrt(nGraphs))
25      height ← ceiling(nGraphs / width)
26      par(mfrow = c(height, width), oma = c(0, 0, 3, 0))
27
28      #set the colours for all weights
29      line.cols.df ← data.frame(Weight = unique(ML.results.df.svmRadial.filtered$Weight), col =
            line.cols[1:length(unique(ML.results.df.svmRadial.filtered$Weight))])
30      line.cols.df ← arrange(line.cols.df, Weight)
31
32      # set y axis limits
33      y.limits ← c(0.6,0.9)
34
35      for(sig in unique(ML.results.df.svmRadial.filtered$sigma)) {
36        tempDF ← filter(ML.results.df.svmRadial.filtered, sigma == sig)
37
38        #find the right colour for the weights displayed
39        line.cols.list ← line.cols.df$col[match(unlist(unique(tempDF$Weight)), line.cols.df$Weight)
              ]
40
41        interaction.plot(x.factor = tempDF$C, trace.factor = tempDF$Weight, response = tempDF$ROC,
              col = line.cols.list, lty = 1, legend = F, xlab = "Cost", ylab = "ROC", ylim = y.
              limits, main = paste("Sigma = ", sig, sep = ""))
42        abline(h = list(0.5,0.6,0.7,0.8,0.9), lty = 2, lwd = 0.3, col = "grey")
43        abline(h = list(0.55,0.65,0.75,0.85), lty = 3, lwd = 0.3, col = "lightgrey")
44      }
45      mtext(paste("SVM Radial Tuning: Day", day.x, ifelse(cumul == TRUE, "_cumulative", "only"),
```

```
          sep = " "), outer = TRUE, cex = 1.5)
46      plot.new()
47
48      # determine the necessary weights for the legend and plot the legend
49      legend.df ← line.cols.df[match(unlist(unique(ML.results.df.svmRadial.filtered$Weight)), line.
            cols.df$Weight),]
50      legend("center", legend = unique(line.cols.df$Weight), lty = 1, lwd = 2, col = line.cols.df$
            col, bty = "n", title = "Weight")
51      dev.off()
52    }
53
54    ### avNNet
55
56    tuning.plot.list ← unique(select(ML.results.df.avNNet, day, cumulative))
57
58    for (i in 1:nrow(tuning.plot.list)) {
59      day.x ← tuning.plot.list[i,]$day
60      cumul ← tuning.plot.list[i,]$cumulative
61
62      plotMiscGraph("MLtuning", paste("avNNet_tuning_day", day.x, ifelse(cumul == TRUE, "_
            cumulative", ""), sep = ""), 4000, 2000)
63
64      # filter for the correct range of days, 3 or 5
65      ML.results.df.avNNet.filtered ← filter(ML.results.df.avNNet, cumulative == cumul, day == day.
            x)
66
67      #set the colours and pch for all weights
68      line.cols.df ← data.frame(size = unique(ML.results.df.avNNet$size), col = line.cols[1:length(
            unique(ML.results.df.avNNet$size))], pch = pch.list[1:length(unique(ML.results.df.avNNet
            $size))])
69      line.cols.df ← arrange(line.cols.df, size)
70
71      # set up the graph layout
72      layout(cbind(1,1,1,2,2,2,3))
73      par(oma = c(0, 0, 3, 0))
74
75      # set y limits for all graphs
76      y.limits ← c(max(min(ML.results.df.avNNet.filtered$ROC),
77                       max(ML.results.df.avNNet.filtered$ROC)-0.1),
78                   max(ML.results.df.avNNet.filtered$ROC) + 0.005)
79
80        for(bag.x in c(TRUE,FALSE)) {
81        tempDF ← filter(ML.results.df.avNNet.filtered, bag == bag.x)
82
83        #find the right colour for the weights displayed
84        line.cols.list ← line.cols.df$col[match(unlist(unique(tempDF$size)), line.cols.df$size)]
85        line.pch.list ← line.cols.df$pch[match(unlist(unique(tempDF$size)), line.cols.df$size)]
86
87        interaction.plot(x.factor = tempDF$decay, trace.factor = tempDF$size, response = tempDF$ROC
              , col = line.cols.list, lty = 1, legend = F, xlab = "Decay", ylab = "ROC", ylim = y.
              limits, main = paste("Bag = ", bag.x, sep = ""), type = "b", pch = line.pch.list, cex
              = 1)
88        abline(h = seq(0.6,0.9,by = 0.01), lty = 2, lwd = 1, col = "lightgrey")
89        abline(h = seq(0.605,0.905,by = 0.01), lty = 3, lwd = 0.8, col = "lightgrey")
90      }
91
92      mtext(paste("avNNet Tuning: Day", day.x, ifelse(cumul == TRUE, "_cumulative", "only"), sep =
            " "), outer = TRUE, cex = 1.5)
93      plot.new()
94
95      legend.df ← line.cols.df[match(unlist(unique(ML.results.df.avNNet.filtered$size)), line.cols.
            df$size),]
96      legend("center", legend = legend.df$size, lty = 1, lwd = 1, col = legend.df$col, bty = "n",
            title = "Size", pch = legend.df$pch)
97      dev.off()
98    }
99
100
101   ### adaboost
102   # determine plotting colours
103   temp.DF ← mutate(ML.results.df.adaboost, legend = paste(day,cumulative))
104   legend.df ← data.frame(code = c("Adaboost.M1", "Real Adaboost", "", sort(unique(temp.DF$legend)
```

```
    )),
105                                     cols = c("black", "black", "black", "grey", "blue", "darkblue
                                            ", "pink", "darkred", "orange", "darkorange", "green", "
                                            darkgreen")[1:(3+length(unique(temp.DF$legend)))],
106                                     labs = c("Adaboost.M1", "Real Adaboost", "", "Day 1", "Day 2"
                                            , "Day 2 cumul", "Day 3", "Day 3 cumul", "Day 4", "Day 4
                                             cumul", "Day 5", "Day 5 cumul")[1:(3+length(unique(temp
                                            .DF$legend)))],
107                                     stringsAsFactors = FALSE)
108
109
110 ML.results.df.adaboost.plot <- mutate(ML.results.df.adaboost, trace = paste(method,day,
        cumulative), legend = paste(day,cumulative))
111
112 nplots <- length(unlist(unique(ML.results.df.adaboost.plot$trace)))
113 nplots.M1 <- length(unlist(unique(filter(ML.results.df.adaboost.plot, method == "Adaboost.M1")$
        trace)))
114 nplots.real <- nplots - nplots.M1
115
116
117 line.cols.plot <- legend.df$col[sort(match(unlist(unique(ML.results.df.adaboost.plot$legend)),
        legend.df$code))]
118 legend.cols <- legend.df$col[sort(c(1,2,3,match(unlist(unique(ML.results.df.adaboost.plot$legend
        )), legend.df$code)))]
119 legend.labs <- legend.df$labs[sort(c(1,2,3,match(unlist(unique(ML.results.df.adaboost.plot$
        legend)), legend.df$code)))]
120
121
122 plotMiscGraph("MLtuning", "adaboost_tuning", 4000, 2000)
123 interaction.plot(x.factor = ML.results.df.adaboost.plot$nIter,
124                  trace.factor = ML.results.df.adaboost.plot$trace,
125                  response = ML.results.df.adaboost.plot$ROC,
126                  type = "c",
127                  trace.label = "Method & Day",
128                  ylab = "AUROC",
129                  xlab = "No. trees",
130                  main = "Adaboost tuning",
131                  col = c(line.cols.plot[1:nplots.M1], line.cols.plot[1:nplots.real]),
132                  lty = c(rep(1, nplots.M1), rep(2, nplots.real)),
133                  legend = FALSE)
134
135 legend("right",
136        lty = c(1,2,0,rep(1, nplots)),
137        col = legend.cols,
138        legend = legend.labs,
139        bty = "n",
140        inset = 0.05)
141 dev.off()
142
143
144
145 ### parRF
146 # determine plotting colours
147 temp.DF <- mutate(ML.results.df.parRF, legend = paste(day,cumulative))
148 legend.df <- legend.df <- data.frame(code = c(sort(unique(temp.DF$legend))),
149                                      cols = c("grey", "blue", "darkblue", "pink", "darkred", "
                                            orange", "darkorange", "green", "darkgreen"),
150                                      labs = c("Day 1", "Day 2", "Day 2 cumul", "Day 3", "Day 3
                                            cumul", "Day 4", "Day 4 cumul", "Day 5", "Day 5 cumul
                                            "),
151                                      ltys = c(1,rep(c(1,2),4)),
152                                      stringsAsFactors = FALSE)
153
154 ML.results.df.parRF.plot <- mutate(ML.results.df.parRF, trace = paste(day,cumulative), legend =
        paste(day,cumulative))
155
156 nplots <- length(unlist(unique(ML.results.df.parRF.plot$trace)))
157
158 line.cols.plot <- legend.df$col[sort(match(unlist(unique(ML.results.df.parRF.plot$legend)),
        legend.df$code))]
159 legend.cols <- legend.df$col[sort(match(unlist(unique(ML.results.df.parRF.plot$legend)), legend.
        df$code))]
```

```
160     legend.labs ← legend.df$labs[sort(match(unlist(unique(ML.results.df.parRF.plot$legend)), legend
            .df$code))]
161     legend.ltys ← legend.df$ltys[sort(match(unlist(unique(ML.results.df.parRF.plot$legend)), legend
            .df$code))]
162
163     plotMiscGraph("MLtuning", "parRF_tuning", 4000, 2000)
164     layout(cbind(1,1,1,1,1,1,2))
165     interaction.plot(x.factor = ML.results.df.parRF.plot$mtry,
166                      trace.factor = ML.results.df.parRF.plot$trace,
167                      response = ML.results.df.parRF.plot$ROC,
168                      type = "c",
169                      trace.label = "Method & Day",
170                      ylab = "AUROC",
171                      xlab = "mtry",
172                      main = "parRF tuning",
173                      col = legend.cols,
174                      lty = legend.ltys,
175                      legend = FALSE,
176                      ylim = c(min(ML.results.df.parRF.plot$ROC), max(ML.results.df.parRF.plot$ROC))
                             )
177     plot.new()
178     legend("center",
179            lty = legend.ltys,
180            col = legend.cols,
181            legend = legend.labs,
182            bty = "n",
183            inset = 0.05)
184     dev.off()
185
186
187
188     #### DeepNN (done in seperate iterations to maintain tuning balance between parameters)
189     plotDeepNNtuningGraphs ← function(iters.in, results.df, iteration) {
190       legend.df ← data.frame(code = c("1", "2", "2 cumul", "3", "3 cumul", "4", "4 cumul", "5", "5
              cumul"),
191                              cols = c("grey", "blue", "darkblue", "pink", "darkred", "orange", "
                                   darkorange", "green", "darkgreen"),
192                              labs = c("Day 1", "Day 2", "Day 2 cumul", "Day 3", "Day 3 cumul", "
                                   Day 4", "Day 4 cumul", "Day 5", "Day 5 cumul"),
193                              ltys = c(1,rep(c(1,2),4)),
194                              stringsAsFactors = FALSE)
195
196       iter.df ← filter(results.df, iter %in% iters.in)
197
198       # iterate through tuning parameter plotting an aggregate graph
199       for (col in colnames(iter.df)[1:14]) {
200         if(length(unique(iter.df[[col]])) > 1) {
201           plotMiscGraph("MLtuning", paste("DeepNN_iteration", iteration, "_", col, sep = ""), 4000,
                  2000)
202           plot.df ← iter.df %>% group_by_(col, "day", "cumulative") %>% summarise(ROC = mean(ROC),
                  dayCumul = paste(day[1],ifelse(cumulative[1] == TRUE, "cumul", ""))) %>% arrange(
                  dayCumul)
203           interaction.plot(x.factor = plot.df[[col]],trace.factor = plot.df$dayCumul, response =
                  plot.df$ROC, pch = 16, col = legend.df$cols, trace.label = "Day", main = paste("
                  Tuning paramater: ", col, sep = ""), lty = legend.df$ltys)
204           dev.off()
205         }
206       }
207     }
208
209     # iter 1
210     plotDeepNNtuningGraphs(iters.in = 1:30, ML.results.df.DeepNN, 1)
211
212     # iter 2
213     plotDeepNNtuningGraphs(iters.in = 31:50, ML.results.df.DeepNN, 2)
214   }
```

2.9.7 plotMLOutputGraph

```
1   # flexible graph plotting function for output of ML tuning
2
```

```
3  source("functions/graph_plotting/plotMiscGraph.R")
4
5  plotMLOutputGraph <- function(plotMetric, plotName, resampleResults, MLmethods, file.name, day.
       range = 5, lines.plot = FALSE, legend.position = "bottomleft") {
6    colour.df <- data.frame(MLmethod = c("adaboost", "avNNet", "parRF", "svmRadialWeights", "glm", "
         DeepNN", "APACHE"),
7                            col = c("darkred", "darkblue", "green", "pink", "blue", "orange", "grey
                                 "),
8                            stringsAsFactors = FALSE)
9
10   plotMiscGraph("Grand_ML_output_graphs", file.name, 4000, 2000)
11
12   plot.df <- filter(resampleResults, day <= day.range)
13
14   apache.present <- 0 %in% plot.df$day
15   if (apache.present) {
16     loc.start <- 4
17     APACHE.df <- filter(plot.df, day == 0)
18     plot.df <- filter(plot.df, day > 0)
19   } else {
20     loc.start <- 1
21   }
22
23
24   labs.all <- c("Day 1", "Day 1\nCumul.", "Day 2", "Day 2\nCumul.", "Day 3", "Day 3\nCumul.", "Day
         4", "Day 4\nCumul.", "Day 5", "Day 5\nCumul.")
25   labs <- labs.all[1:(day.range * 2)]
26
27   # generate list of plotted factors
28   plottingFactors <- unique(plot.df %>% filter(day > 0) %>% select(MLmethod, day, cumulative))
29
30   # determine how wide each day plot should be based on how many ML methods are used plus a
         visual buffer
31   no.x.plots <- length(MLmethods) + 2
32
33   # determine the "base" points from which to reference the plots
34   loc.base <- c(loc.start, (1:(day.range + day.range - 1) * no.x.plots - (no.x.plots - loc.start))
         ) #determine start of each day, including space for cumulative plots
35
36   # generate df for calculating appropriate y axis
37   ylims.df <- filter(resampleResults, MLmethod %in% MLmethods)
38
39   # set limits on the axes
40   x.limits <- c(0.5, max(loc.base) - 1.5 + no.x.plots) # set x axis limits
41   y.limits <- c(min(ylims.df[[plotMetric]]), max(ylims.df[[plotMetric]]))
42
43   # define the plotting formula
44   formula <- formula(paste(plotMetric,"~ cumulative + day", sep = ""))
45
46   # plot day 1 cumulative outside the plot range as it never exists
47   loc.base[2] <- 1000
48
49   for(i in 1:length(MLmethods)) {
50     addBool <- ifelse(i == 1, FALSE, TRUE) # is this the first graph?
51
52     filtered.plot.df <- filter(plot.df, MLmethod == MLmethods[i])
53
54     loc.strip.all <- loc.base + i - 1
55
56     filtered.plot.df.2 <- filtered.plot.df
57     filtered.plot.df.2$day <- factor(filtered.plot.df.2$day, levels = 1:day.range)
58     filtered.plot.df.2$cumulative <- factor(filtered.plot.df.2$cumulative, levels = c(FALSE,TRUE))
59
60     stripchart(formula,
61                filtered.plot.df.2,
62                vertical = TRUE,
63                at = loc.strip.all,
64                xlim = x.limits,
65                ylim = y.limits,
66                pch = 16,
67                cex = 0.5,
68                method = "jitter",
```

```
69                col = as.character(colour.df$col[colour.df$MLmethod == MLmethods[i]]),
70                las = 2,
71                xaxt = "n",
72                ylab = plotName,
73                main = paste("Comparison of ML methods tuned to AUROC: difference in ", plotName,
                       ".\nMean +/- 1.96SEM", sep = ""),
74                add = addBool)
75
76      # add means and error bars dynamically
77      mutated.plot.df <- mutate_(filtered.plot.df, outVar = plotMetric)
78      summary.df <- mutated.plot.df %>%
79        group_by(day, cumulative) %>%
80        summarise(mean = mean(outVar),
81                  sd = sd(outVar),
82                  sem = sd / sqrt(n()),
83                  ul = mean + 1.96 * sem,
84                  ll = mean - 1.96 * sem)
85
86      total.df <- expand.grid(day = 1:day.range, cumulative = c(TRUE,FALSE))
87      total.df <- suppressMessages(left_join(total.df, summary.df))
88
89      for(col in colnames(total.df)) {
90        total.df[[col]] <- ifelse(is.na(total.df[[col]]), 0, total.df[[col]])
91      }
92
93      total.df <- arrange(total.df, day, cumulative)
94
95      segments(x0 = c(loc.strip.all - 0.3, loc.strip.all - 0.3, loc.strip.all - 0.3, loc.strip.all)
             ,
96               x1 = c(loc.strip.all + 0.3, loc.strip.all + 0.3, loc.strip.all + 0.3, loc.strip.all)
                    ,
97               y0 = c(total.df$ul, total.df$ll, total.df$mean, total.df$ul),
98               y1 = c(total.df$ul, total.df$ll, total.df$mean, total.df$ll),
99               lwd = rep(c(1,1,2,0.8), each = length(loc.strip.all)))
100
101     ### add lines to indicate trend
102     if (lines.plot == TRUE) {
103       lines(filter(total.df, cumulative == FALSE)$mean, lwd = 0.5, lty = 2, x = loc.strip.all[c(1
              ,3,5,7,9)], col = as.character(colour.df$col[colour.df$MLmethod == MLmethods[i]]))
104       lines(c(filter(total.df, day == 1, cumulative == FALSE)$mean,filter(total.df, cumulative ==
               TRUE, day > 1)$mean), lwd = 0.5, lty = 1, x = loc.strip.all[c(1,4,6,8,10)], col = as.
             character(colour.df$col[colour.df$MLmethod == MLmethods[i]]))
105     }
106
107     # add APACHE if appropriate
108     if(MLmethods[i] == "glm" & apache.present) {
109       stripchart(formula,
110                  APACHE.df,
111                  vertical = TRUE,
112                  at = 1,
113                  pch = 16,
114                  cex = 0.5,
115                  method = "jitter",
116                  col = as.character(colour.df$col[colour.df$MLmethod == "glm"]),
117                  las = 2,
118                  add = TRUE)
119
120       mean.APACHE <- mean(APACHE.df[[plotMetric]])
121       sem.APACHE <- 1.96 * (sd(APACHE.df[[plotMetric]]) / sqrt(nrow(APACHE.df)))
122       ul <- mean.APACHE + sem.APACHE
123       ll <- mean.APACHE - sem.APACHE
124
125       segments(x0 = c(0.7,0.7,0.7,1),
126                x1 = c(1.3,1.3,1.3,1),
127                y0 = c(ul, ll, mean.APACHE, ul),
128                y1 = c(ul, ll, mean.APACHE, ll),
129                lwd = c(1,1,2,0.8))
130
131       axis(side = 1, at = 1, labels = "APACHE", tick = FALSE)
132     }
133   }
134   colour.df <- arrange(colour.df, MLmethod)
```

```
135   legend(legend.position, inset = 0.02, bty = "n", pch = 16, legend = sort(MLmethods), col = (
          colour.df$col[colour.df$MLmethod %in% MLmethods]))
136
137   axis(side = 1, at = loc.base + ((no.x.plots - 3) / 2), labels = labs, tick = FALSE)
138
139   dev.off()
140 }
```

2.9.8 plotMLOutputGraph_vImp

```
1   # flexible function to plot the output graphs taking into account the variability of the
        imputations in the error bars and plotting the mean for each fold across all imputations
2
3   plotMLOutputGraph_vImp ← function(imputationResampleResultsALL,
4                                     MLmethods,
5                                     plotMetric = "AUROC",
6                                     plotName,
7                                     legendPosition = "topright",
8                                     title = TRUE,
9                                     legend = TRUE,
10                                    errorBarScale = 0.3,
11                                    addAPACHEday1 = FALSE,
12                                    yLabel = plotMetric,
13                                    height = 2000,
14                                    width = 4000,
15                                    vLines = FALSE,
16                                    barColour = "darkgrey",
17                                    dotSize = 0.5,
18                                    lineThickness = 1) {
19
20    # correct methodological differences in PPV/NPV/Sens/Spec
21    if ("DeepNN" %in% MLmethods) {
22      newTempSet ← imputationResampleResultsALL
23      newTempSet[newTempSet$MLmethod == "DeepNN", grepl("PosPredValue", colnames(
            imputationResampleResultsALL))] ← 1 - imputationResampleResultsALL[
            imputationResampleResultsALL$MLmethod == "DeepNN", grepl("PosPredValue", colnames(
            imputationResampleResultsALL))]
24      newTempSet[newTempSet$MLmethod == "DeepNN", grepl("NegPredValue", colnames(
            imputationResampleResultsALL))] ← 1 - imputationResampleResultsALL[
            imputationResampleResultsALL$MLmethod == "DeepNN", grepl("NegPredValue", colnames(
            imputationResampleResultsALL))]
25
26      newTempSet[newTempSet$MLmethod == "DeepNN", grepl("Sensitivity", colnames(
            imputationResampleResultsALL))] ← 1 - imputationResampleResultsALL[
            imputationResampleResultsALL$MLmethod == "DeepNN", grepl("Sensitivity", colnames(
            imputationResampleResultsALL))]
27      newTempSet[newTempSet$MLmethod == "DeepNN", grepl("Specificity", colnames(
            imputationResampleResultsALL))] ← 1 - imputationResampleResultsALL[
            imputationResampleResultsALL$MLmethod == "DeepNN", grepl("Specificity", colnames(
            imputationResampleResultsALL))]
28
29      newTempSet[newTempSet$MLmethod == "DeepNN", grepl("Accuracy.balanced", colnames(
            imputationResampleResultsALL))] ← 1 - imputationResampleResultsALL[
            imputationResampleResultsALL$MLmethod == "DeepNN", grepl("Accuracy.balanced", colnames(
            imputationResampleResultsALL))]
30
31      imputationResampleResultsALL ← newTempSet
32    }
33
34    # prepare data
35    imputation.resampleResults.ALL.pooled ← imputationResampleResultsALL %>%
36                                              group_by(MLmethod, day, cumulative, Resample) %>%
37                                                summarise(MetricMean = mean(!!sym(plotMetric), na
                                                      .rm = TRUE),
38                                                          ImputVar = sd(!!sym(plotMetric), na.rm
                                                              = TRUE)^2)
39
40    imputation.resampleResults.ALL.pooled.summarised ← imputation.resampleResults.ALL.pooled %>%
41                                                         group_by(MLmethod, day, cumulative) %>%
42                                                             summarise(MetricMeanAvg = mean(
                                                                  MetricMean, na.rm = TRUE),
```

```
43                                                                    FoldVar = sd(MetricMean,
                                                                          na.rm = TRUE)^2,
44                                                                    ImputVarAvg = mean(
                                                                          ImputVar, na.rm =
                                                                          TRUE),
45                                                                    TotalVar = FoldVar +
                                                                          ImputVarAvg,
46                                                                    TotalSD = sqrt(TotalVar),
47                                                                    TotalSEM = TotalSD / sqrt
                                                                          (n()),
48                                                                    UL95 = MetricMeanAvg + 1.
                                                                          96 * TotalSEM,
49                                                                    LL95 = MetricMeanAvg - 1.
                                                                          96 * TotalSEM,
50                                                                    UL2SD = MetricMeanAvg + 2
                                                                           * TotalSD,
51                                                                    LL2SD = MetricMeanAvg - 2
                                                                           * TotalSD)
52
53
54    # define colours
55    colour.df <- arrange(data.frame(MLmethod = c("adaboost", "avNNet", "parRF", "svmRadialWeights",
          "glm", "DeepNN", "APACHE"),
56                                    col = c("darkred", "red", "darkgreen", "pink", "blue", "orange"
                                              , "black"),
57                                    stringsAsFactors = FALSE), MLmethod)
58
59    # filter for ML methods
60    plot.df <- filter(imputation.resampleResults.ALL.pooled, MLmethod %in% MLmethods)
61    plot.limits.df <- filter(imputation.resampleResults.ALL.pooled.summarised, MLmethod %in%
          MLmethods)
62
63    # Generate labels
64    labs.1 <- c("", "", "simple", "cumul.", "simple", "cumul.", "simple", "cumul.", "simple", "cumul
          .")
65    labs.2 <- c("  \nDay 1", "  \nDay 2", "  \nDay 3", "  \nDay 4", "  \nDay 5")
66    labs.apache <- c("Day 1", "Day 2", "Day 3", "Day 4", "Day 5")
67
68    # determine how wide each day plot should be based on how many ML methods are used plus a
          visual buffer
69    no.x.plots <- length(MLmethods) + 2
70
71    # determine the "base" points from which to reference the plots, with different method if only
          APACHE is to be plotted
72    if(paste(MLmethods, collapse = "") == "APACHE") {
73      loc.base <- c(1, 100, 2, 100, 3, 100, 4, 100, 5, 100)
74      x.limits <- c(0.5,5.5)
75    } else {
76      loc.base <- c(1, 1:9 * no.x.plots - (no.x.plots - 1)) #determine start of each day, including
            space for cumulative plots
77      x.limits <- c(0.5, max(loc.base) - 1.5 + no.x.plots) # set x axis limits
78    }
79
80    # set limits on the y axis
81    y.limits <- c(min(c(min(plot.limits.df$LL2SD),
82                        min(plot.df$MetricMean))),
83                  max(c(max(plot.limits.df$UL2SD),
84                        max(plot.df$MetricMean))))
85
86    # plot day 1 cumulative outside the plot range as it never exists
87    loc.base[2] <- 1000
88
89    if (addAPACHEday1 == TRUE) loc.base <- loc.base + 1
90
91    # set up grph and plot by ML method as segments
92    plotMiscGraph("PooledImputationsMLoutput", plotName, width, height)
93
94    j <- 1
95    for(i in c(1:length(MLmethods),1)) {
96      addBool <- ifelse(j == 1, FALSE, TRUE) # is this the first graph?
97
98      plot.df.filtered <- filter(plot.df, MLmethod == MLmethods[i])
```

```
99      plot.limits.df.filtered ← filter(plot.limits.df, MLmethod == MLmethods[i])
100
101     loc.strip ← loc.base + i - 1
102
103     if (addBool == TRUE) {
104       loc.bars ← loc.strip[c(1,3:10)]
105
106       segments(x0 = c(loc.bars - errorBarScale, loc.bars - errorBarScale, loc.bars -
              errorBarScale, loc.bars),
107                x1 = c(loc.bars + errorBarScale, loc.bars + errorBarScale, loc.bars +
                     errorBarScale, loc.bars),
108                y0 = c(plot.limits.df.filtered$UL2SD, plot.limits.df.filtered$LL2SD, plot.limits.
                     df.filtered$MetricMeanAvg, plot.limits.df.filtered$UL2SD),
109                y1 = c(plot.limits.df.filtered$UL2SD, plot.limits.df.filtered$LL2SD, plot.limits.
                     df.filtered$MetricMeanAvg, plot.limits.df.filtered$LL2SD),
110                lwd = lineThickness * rep(c(1,1,2,0.8), each = length(loc.bars)),
111                #col = colour.df$col[colour.df$MLmethod == MLmethods[i]],
112                col = barColour)
113     }
114
115     stripchart(MetricMean ~ cumulative + day,
116                plot.df.filtered,
117                vertical = TRUE,
118                at = loc.strip,
119                xlim = x.limits,
120                ylim = y.limits,
121                pch = 16,
122                cex = dotSize,
123                method = "jitter",
124                las = 2,
125                col = ifelse(j == 1, "white", colour.df$col[colour.df$MLmethod == MLmethods[i]]),
126                xaxt = "n",
127                ylab = yLabel,
128                main = ifelse(title == TRUE, paste("Comparison of ML methods tuned to AUROC: ",
                     plotName, ".\nMean +/- 1.96 SD", sep = ""), NA),
129                add = addBool)
130
131     j ← j + 1
132   }
133
134   if (addAPACHEday1 == TRUE) {
135     loc.apache ← 1
136
137     apacheDay1.df ← filter(imputation.resampleResults.ALL.pooled, MLmethod == "APACHE", day == 1)
138     apacheDay1.limits.df ← filter(imputation.resampleResults.ALL.pooled.summarised, MLmethod == "
          APACHE", day == 1)
139
140     segments(x0 = c(loc.apache - errorBarScale, loc.apache - errorBarScale, loc.apache -
          errorBarScale, loc.apache),
141              x1 = c(loc.apache + errorBarScale, loc.apache + errorBarScale, loc.apache +
                   errorBarScale, loc.apache),
142              y0 = c(apacheDay1.limits.df$UL2SD, apacheDay1.limits.df$LL2SD, apacheDay1.limits.df$
                   MetricMeanAvg, apacheDay1.limits.df$UL2SD),
143              y1 = c(apacheDay1.limits.df$UL2SD, apacheDay1.limits.df$LL2SD, apacheDay1.limits.df$
                   MetricMeanAvg, apacheDay1.limits.df$LL2SD),
144              lwd = lineThickness * rep(c(1,1,2,0.8), each = length(loc.apache)),
145              #col = colour.df$col[colour.df$MLmethod == MLmethods[i]],
146              col = barColour)
147
148     stripchart(MetricMean ~ cumulative + day,
149                apacheDay1.df,
150                vertical = TRUE,
151                at = loc.apache,
152                pch = 16,
153                cex = dotSize,
154                method = "jitter",
155                las = 2,
156                col = colour.df$col[colour.df$MLmethod == "APACHE"],
157                xaxt = "n",
158                main = ifelse(title == TRUE, paste("Comparison of ML methods tuned to AUROC: ",
                     plotName, ".\nMean +/- 1.96 SD", sep = ""), NA),
159                add = TRUE)
```

```
  }

  lab.pos.1 ← loc.base + ((no.x.plots - 3) / 2)
  lab.pos.1[1] ← ifelse(addAPACHEday1 == TRUE, lab.pos.1[1] - 0.5, lab.pos.1[1])
  lab.pos.2 ← c(lab.pos.1[1],
                mean(c(lab.pos.1[3],lab.pos.1[4])),
                mean(c(lab.pos.1[5],lab.pos.1[6])),
                mean(c(lab.pos.1[7],lab.pos.1[8])),
                mean(c(lab.pos.1[9],lab.pos.1[10])))

  if(paste(MLmethods, collapse = "") == "APACHE") {
    axis(side = 1, at = loc.base[c(1,3,5,7,9)], labels = labs.apache, tick = FALSE)
  } else {
    axis(side = 1, at = lab.pos.1, labels = labs.1, tick = FALSE)
    axis(side = 1, at = lab.pos.2, labels = labs.2, tick = FALSE, line = NA, padj = 1, font = 2)
  }

  if(vLines == TRUE) {
    line.pos.main ← c(mean(c(lab.pos.2[1],lab.pos.2[2])) - 2,
                      mean(c(lab.pos.2[2],lab.pos.2[3])),
                      mean(c(lab.pos.2[3],lab.pos.2[4])),
                      mean(c(lab.pos.2[4],lab.pos.2[5])))

    segments(y0 = 0, y1 = 1,
             x0 = line.pos.main, x1 = line.pos.main, lty = 3, col = "grey")
  }

  # choose what to put in the legend
  if(addAPACHEday1== TRUE) {
    MLmethods.legend ← c("APACHE",MLmethods)
  } else {
    MLmethods.legend ← MLmethods
  }

  if (legend == TRUE) legend(legendPosition, inset = 0.02, bty = "n", pch = 16, legend = sort(
      MLmethods.legend), col = (colour.df$col[colour.df$MLmethod %in% MLmethods.legend])) # make
       the legend

  dev.off()
}
```

2.9.9 plotImputationResults

```
# plots a graph of the results of the nine imputations for each day

library(dplyr)

source("functions/graph_plotting/plotMiscGraph.R")

plotImputationResults ← function(imputationResultsDF, MLmethod, title = FALSE) {
  plotMiscGraph("imputation_variation", paste("ImpVar_", MLmethod, sep = ""), 4000, 2000)

  imputationResultsDF ← imputationResultsDF %>% mutate(dayImp = paste(day, cumulative, imputation
      , sep = ""),
                                                       ROCSEM = (ROCSD / sqrt(20)),
                                                       ROCUL = ROC + 2 * ROCSD,
                                                       ROCLL = ROC - 2 * ROCSD) %>%
                                                arrange(dayImp)

  loc.strip ← c(1:9,11:19,21:29,31:39,41:49,51:59,61:69,71:79,81:89)
  ylims ← c(min(imputationResultsDF$ROCLL),
            max(imputationResultsDF$ROCUL))

  stripchart(ROC ~ dayImp,
             imputationResultsDF,
             vertical = TRUE,
             pch = "-",
             cex = 2,
             las = 2,
             ylab = "AUC",
             at = loc.strip,
```

```
28              xaxt = "n",
29              xlim = c(0,90),
30              ylim = ylims,
31              main = ifelse(title == TRUE, paste("AUC of 20 folds of 9 ", MLmethod, " models for
                    each of 9 imputations.\nMean +/- 2SD", sep = ""), NA))
32
33    stripchart(ROCUL ~ dayImp,
34              imputationResultsDF,
35              vertical = TRUE,
36              pch = "-",
37              cex = 1,
38              at = loc.strip,
39              add = TRUE)
40
41    stripchart(ROCLL ~ dayImp,
42              imputationResultsDF,
43              vertical = TRUE,
44              pch = "-",
45              cex = 1,
46              at = loc.strip,
47              add = TRUE)
48
49    segments(x0 = loc.strip, x1 = loc.strip,
50             y0 = imputationResultsDF$ROCUL, y1 = imputationResultsDF$ROCLL,
51             lwd = 1)
52
53    # add x-axis
54    labs.1 <- c("", "simple", "cumul.", "simple", "cumul.", "simple", "cumul.", "simple", "cumul.")
55    labs.2 <- c("  \nDay 1", "  \nDay 2", "  \nDay 3", "  \nDay 4", "  \nDay 5")
56
57    lab.pos.1 <- seq(5,85,10)
58    lab.pos.2 <- c(5, seq(20,80,20))
59
60    axis(side = 1, at = lab.pos.1, labels = labs.1, tick = FALSE)
61    axis(side = 1, at = lab.pos.2, labels = labs.2, tick = FALSE, line = NA, padj = 1, font = 2)
62
63    # add vertical lines
64    line.pos.main <- c(seq(10,70,20))
65
66    segments(y0 = 0, y1 = 1,
67             x0 = line.pos.main, x1 = line.pos.main, lty = 3, col = "grey")
68
69
70    dev.off()
71  }
72
73  plotImputationResults_dayCompare <- function(imputationResultsDF.1, imputationResultsDF.2,
        MLmethod, group1name, group2name, title = FALSE, day) {
74    plotMiscGraph("imputation_variation", paste("ImpVar_", MLmethod, sep = ""), 4000, 2000)
75
76    imputationResultsDF <- rbind(mutate(imputationResultsDF.1, group = 1),
77                                 mutate(imputationResultsDF.2, group = 2))
78
79    imputationResultsDF <- imputationResultsDF %>% mutate(dayImp = paste(day, cumulative, imputation
          , sep = ""),
80                                                          ROCSEM = (ROCSD / sqrt(20)),
81                                                          ROCUL = ROC + 2 * ROCSD,
82                                                          ROCLL = ROC - 2 * ROCSD) %>%
83                                                          arrange(group, dayImp)
84
85    loc.strip <- c(1:9,11:19,21:29,31:39)
86    ylims <- c(min(imputationResultsDF$ROCLL),
87              max(imputationResultsDF$ROCUL))
88
89    stripchart(ROC ~ dayImp + group,
90              imputationResultsDF,
91              vertical = TRUE,
92              pch = "-",
93              cex = 2,
94              las = 2,
95              ylab = "AUC",
96              at = loc.strip,
```

```
              xaxt = "n",
              xlim = c(0,40),
              ylim = ylims,
              main = ifelse(title == TRUE, paste("AUC of 20 folds of 9 ", MLmethod, " models for
                  each of 9 imputations.\nMean +/- 2SD", sep = ""), NA))

  stripchart(ROCUL ~ dayImp + group,
              imputationResultsDF,
              vertical = TRUE,
              pch = "-",
              cex = 1,
              at = loc.strip,
              add = TRUE)

  stripchart(ROCLL ~ dayImp + group,
              imputationResultsDF,
              vertical = TRUE,
              pch = "-",
              cex = 1,
              at = loc.strip,
              add = TRUE)

  segments(x0 = loc.strip, x1 = loc.strip,
           y0 = imputationResultsDF$ROCUL, y1 = imputationResultsDF$ROCLL,
           lwd = 1)

  groupMeans <- imputationResultsDF %>% group_by(group, cumulative) %>% summarise(meanROC = mean(
      ROC))

  segments(x0 = c(1,11,21,31,-10,-10,-10,-10), x1 = c(9,19,29,39,50,50,50,50),
           y0 = groupMeans$meanROC, y1 = groupMeans$meanROC,
           lwd = rep(c(2,1),each = 4), col = c("blue", "red", "blue", "red"), lty = rep(c(1,3),
               each = 4))

  # add x-axis
  labs.1 <- c("simple", "cumul.", "simple", "cumul.")
  labs.2 <- c(group1name, group2name)

  lab.pos.1 <- seq(5,35,10)
  lab.pos.2 <- seq(10,30,20)

  axis(side = 1, at = lab.pos.1, labels = labs.1, tick = FALSE)
  axis(side = 1, at = lab.pos.2, labels = labs.2, tick = FALSE, line = NA, padj = 1, font = 2)

  # add vertical lines
  line.pos.main <- mean(lab.pos.2)

  segments(y0 = 0, y1 = 1,
           x0 = line.pos.main, x1 = line.pos.main, lty = 1, col = "grey")


  dev.off()
}
```

2.9.10 plotPredictionsBeanplotPanel

```
# plots either one beanplot of a panel of beanplots showing the distributino of the named
    variable for correct/incorrect predictions of alive/deceased classifier values

plotPredictionsBeanplot <- function(inputDF, day.in, cumul.in, variableName, legend = TRUE,
    ylimits = NA, density = 3, title = TRUE, xlabel = "Actual outcome", ylabel = TRUE) {
  plot.df <- filter(inputDF, day == day.in, cumulative == cumul.in) %>% select_("obs", "correct",
      variableName)

  levels(plot.df$obs) <- c("deceased", "alive")

  colnames(plot.df)[3] <- "variable"

  plot.df.correct <- filter(plot.df, correct == TRUE)
  plot.df.incorrect <- filter(plot.df, correct == FALSE)

```

```
13    x.limits ← c(0.3,2.7)
14
15    beanplot(variable ~ obs,
16             plot.df.correct,
17             side = "first",
18             horizontal = FALSE,
19             xlim = x.limits,
20             ylim = ylimits,
21             col=c("darkseagreen2","darkseagreen2", "lightgrey"), bw = density, ll = 0.002,
22             what = c(0,1,0,0), log = "", las = 1,
23             ylab = ifelse(ylabel == TRUE, variableName, NA),
24             main = ifelse(title == TRUE, paste("Beanplots of", variableName, "for correct and
                   incorrect classifications on day", day.in, ifelse(cumul.in == TRUE, "cumul.", "
                   simple"), sep = " "), NA))
25
26    axis(1, at = 1.5, labels = xlabel, tick = FALSE, line = 1, font = 2)
27
28    beanplot(variable ~ obs, plot.df.incorrect,
29             side = "second",
30             col=c("lightpink2","lightpink2", "lightgrey"),
31             add = TRUE, bw = density, ll = 0.002,
32             what = c(0,1,0,0))
33
34    means.df ← plot.df %>% group_by(obs, correct) %>% summarise(mean = mean(variable),
35                                                                sd = sd(variable),
36                                                                upSD = mean + sd,
37                                                                downSD = mean - sd)
38    bars.list ← unlist(c(filter(means.df, obs == "deceased", correct == TRUE)[,c("mean", "upSD", "
          downSD")],
39                         filter(means.df, obs == "deceased", correct == FALSE)[,c("mean", "upSD",
                               "downSD")],
40                         filter(means.df, obs == "alive", correct == TRUE)[,c("mean", "upSD", "
                               downSD")],
41                         filter(means.df, obs == "alive", correct == FALSE)[,c("mean", "upSD", "
                               downSD")]))
42    segments(x0 = rep(c(1,2), each = 6), x1 = rep(c(0.55,1.45,1.55,2.45), each = 3),
43             y0 = bars.list, y1 = bars.list,
44             col = "black",
45             lwd = rep(c(1.5,1,1), times = 4),
46             lty = rep(c(1,2,2), times = 4))
47
48    if(legend == TRUE) legend('topright', fill=c('darkseagreen2','lightpink2'), legend= c("correct"
          , "incorrect"), bty ="n")
49  }
50
51  plotPredictionsBeanplotPanel ← function(variable, inputDF, density = 1, ylimits = NA, title =
        TRUE, footer = TRUE, MLmethod) {
52
53    plotMiscGraph("PredictionBeanplotPanels",
54                  paste("beanplot_predictions_panel", variable, MLmethod, sep = "_"),
55                  width = 4000,
56                  height = 2000)
57
58    par(mfrow = c(2,5), oma = c(ifelse(footer == TRUE, 6, 1), 2, ifelse(title == TRUE, 6, 1), 2),
          mar = c(3, 3, 1.5, 0))
59
60    day.cumul ← expand.grid(day = 1:5, cumul = c(FALSE,TRUE))
61    for (i in 1:nrow(day.cumul)) {
62      if (day.cumul$day[i] == 1 & day.cumul$cumul[i] == TRUE) {
63        plot.new()
64        legend('center', fill=c('darkseagreen2','lightpink2'), legend= c("correct", "incorrect"),
              bty ="n")
65      } else {
66        plotPredictionsBeanplot(inputDF = inputDF,
67                                day.in = day.cumul$day[i],
68                                cumul.in = day.cumul$cumul[i],
69                                variableName = variable,
70                                legend = FALSE,
71                                density = density,
72                                ylimits = ylimits,
73                                title = FALSE,
74                                xlabel = paste("Day",
```

```
75                                                  day.cumul$day[i],
76                                                  ifelse(day.cumul$cumul[i] == TRUE, "cumul.", "simple
                                                        "), sep = " "),
77                                    ylabel = ifelse(day.cumul$day[i] == 1 | (day.cumul$day[i] == 2 &
                                          day.cumul$cumul[i] == TRUE), TRUE, FALSE))
78       }
79     }
80
81     if (title == TRUE) mtext(paste("Beanplots of distributions of", variable, "for correct and
           incorrect classifications, split by actual outcome.\n", MLmethod, "classifier.", sep = " "
           ), outer = TRUE, cex = 1.2)
82     if (footer == TRUE) mtext("Thick bars represent mean and dashed bars mean +/- one standard
           deviation", outer = TRUE, cex = 0.8, side = 1, at = 0.2, line = 2)
83
84     dev.off()
85   }
```
